# Atherosclerosis quantification and cardiovascular risk: the ISCHEMIA trial

**DOI:** 10.1093/eurheartj/ehae471

**Published:** 2024-08-05

**Authors:** Nick S Nurmohamed, James K Min, Rebecca Anthopolos, Harmony R Reynolds, James P Earls, Tami Crabtree, G B John Mancini, Jonathon Leipsic, Matthew J Budoff, Cameron J Hague, Sean M O'Brien, Gregg W Stone, Jeffrey S Berger, Robert Donnino, Mandeep S Sidhu, Jonathan D Newman, William E Boden, Bernard R Chaitman, Peter H Stone, Sripal Bangalore, John A Spertus, Daniel B Mark, Leslee J Shaw, Judith S Hochman, David J Maron

**Affiliations:** Department of Cardiology, Amsterdam UMC, Vrije Universiteit Amsterdam, De Boelelaan 1117, 1081 HV Amsterdam, The Netherlands; Department of Vascular Medicine, Amsterdam UMC, University of Amsterdam, Meibergdreef 9, 1105 AZ Amsterdam, The Netherlands; Division of Cardiology, The George Washington University School of Medicine, 2150 Pennsylvania Avenue NW, Washington, DC 20037, USA; Cleerly, Inc, Denver, CO, USA; New York University Grossman School of Medicine, New York, NY, USA; New York University Grossman School of Medicine, New York, NY, USA; Cleerly, Inc, Denver, CO, USA; Department of Radiology, The George Washington University School of Medicine, Washington, DC, USA; Cleerly, Inc, Denver, CO, USA; Centre for Cardiovascular Innovation, University of British Columbia, Vancouver, British Columbia, Canada; Centre for Cardiovascular Innovation, University of British Columbia, Vancouver, British Columbia, Canada; Lundquist Institute, Torrance, CA, USA; Centre for Cardiovascular Innovation, University of British Columbia, Vancouver, British Columbia, Canada; Duke Clinical Research Institute, Durham, NC, USA; Icahn School of Medicine at Mount Sinai, New York, NY, USA; New York University Grossman School of Medicine, New York, NY, USA; New York University Grossman School of Medicine, New York, NY, USA; Albany Medical College, Albany, NY, USA; New York University Grossman School of Medicine, New York, NY, USA; VA New England Healthcare System, Boston University School of Medicine, Boston, MA, USA; St Louis University School of Medicine Center for Comprehensive Cardiovascular Care, St Louis, MO, USA; Brigham and Women’s Hospital, Boston, MA, USA; New York University Grossman School of Medicine, New York, NY, USA; University of Missouri—Kansas City’s Healthcare Institute for Innovations in Quality and Saint Luke’s Mid America Heart Institute, Kansas City, MO, USA; Duke Clinical Research Institute, Durham, NC, USA; Bronfman Department of Medicine (Cardiology), Icahn School of Medicine at Mount Sinai, New York, NY, USA; New York University Grossman School of Medicine, New York, NY, USA; Department of Medicine, Stanford University School of Medicine, Stanford, CA, USA

**Keywords:** Atherosclerosis, CCTA, Ischaemia, Artificial intelligence, Plaque, Coronary artery disease

## Abstract

**Background and Aims:**

The aim of this study was to determine the prognostic value of coronary computed tomography angiography (CCTA)–derived atherosclerotic plaque analysis in ISCHEMIA.

**Methods:**

Atherosclerosis imaging quantitative computed tomography (AI-QCT) was performed on all available baseline CCTAs to quantify plaque volume, composition, and distribution. Multivariable Cox regression was used to examine the association between baseline risk factors (age, sex, smoking, diabetes, hypertension, ejection fraction, prior coronary disease, estimated glomerular filtration rate, and statin use), number of diseased vessels, atherosclerotic plaque characteristics determined by AI-QCT, and a composite primary outcome of cardiovascular death or myocardial infarction over a median follow-up of 3.3 (interquartile range 2.2–4.4) years. The predictive value of plaque quantification over risk factors was compared in an area under the curve (AUC) analysis.

**Results:**

Analysable CCTA data were available from 3711 participants (mean age 64 years, 21% female, 79% multivessel coronary artery disease). Amongst the AI-QCT variables, total plaque volume was most strongly associated with the primary outcome (adjusted hazard ratio 1.56, 95% confidence interval 1.25–1.97 per interquartile range increase [559 mm^3^]; *P* = .001). The addition of AI-QCT plaque quantification and characterization to baseline risk factors improved the model’s predictive value for the primary outcome at 6 months (AUC 0.688 vs. 0.637; *P* = .006), at 2 years (AUC 0.660 vs. 0.617; *P* = .003), and at 4 years of follow-up (AUC 0.654 vs. 0.608; *P* = .002). The findings were similar for the other reported outcomes.

**Conclusions:**

In ISCHEMIA, total plaque volume was associated with cardiovascular death or myocardial infarction. In this highly diseased, high-risk population, enhanced assessment of atherosclerotic burden using AI-QCT-derived measures of plaque volume and composition modestly improved event prediction.

## Structured Graphical Abstract

**Structured Graphical Abstract:**
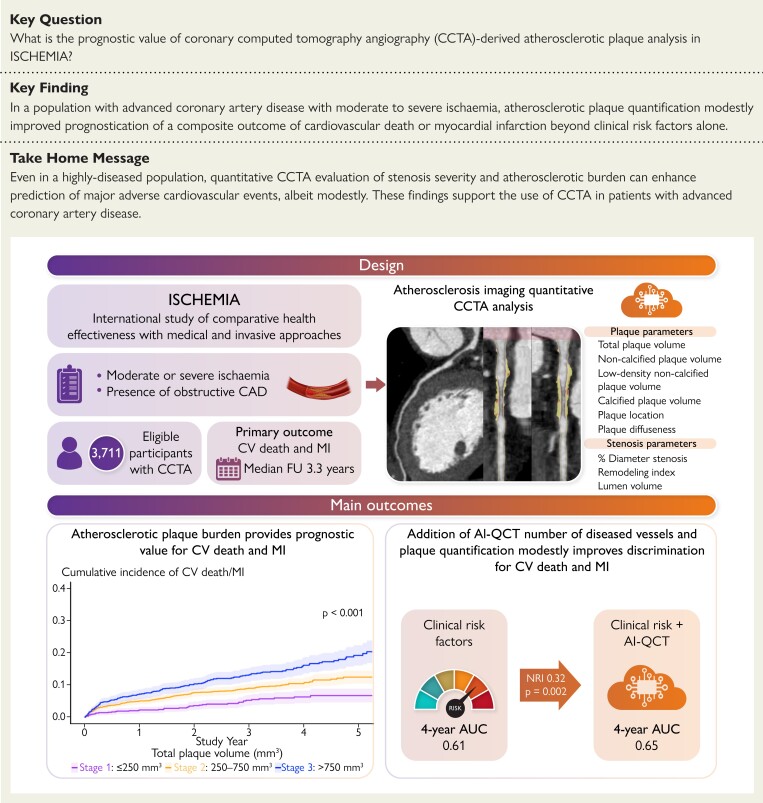
Study design and main outcomes of the current study. AI-QCT, atherosclerosis imaging quantitative computed tomography; AUC, area under the curve; CAD, coronary artery disease; CCTA, coronary computed tomography angiography; CV, cardiovascular; FU, follow-up; MI, myocardial infarction; NRI, net reclassification improvement.

**See the editorial comment for this article 'Vulnerable plaque and major adverse cardiovascular events: anatomy of a failure', by C.N.B. Merz, https://doi.org/10.1093/eurheartj/ehae553.**

## Introduction

Coronary computed tomography angiography (CCTA) has increasingly become a first-line diagnostic alternative to invasive coronary angiography for defining the presence and severity of atherosclerotic coronary artery disease (CAD). Beyond providing an accurate characterization of lumen stenosis,^1–4^ the three-dimensional volumetric nature of CCTA also permits the assessment of the extent and characteristics of atherosclerotic plaque itself.^1,2,5–8^ As such, CCTA allows for identification and quantification of calcified, non-calcified, and low-density necrotic core components, as well as measures of vascular remodelling and the identification of high-risk plaque phenotypes.^8^ The burden of atherosclerosis and presence of high-risk plaque phenotypes have previously shown to improve identification of patients at increased risk of subsequent major adverse cardiac events (MACEs), although improvement varied between studies.^9–13^

In the Scottish Computed Tomography of the HEART (SCOT-HEART) trial, death from CAD or nonfatal myocardial infarction (MI) was three times more frequent in participants with adverse plaque characteristics and low-attenuation plaque burden was the strongest predictor of MI, irrespective of cardiovascular risk score, coronary artery calcium score, or stenosis.^12^ In the Prospective Multicentre Imaging Study for Evaluation of Chest Pain (PROMISE) trial, high-risk plaque was associated with a 70% increased risk of MACE, independent of other risk factors or the presence of obstructive disease.^10^ In the Coronary CT Angiography Evaluation for Clinical Outcomes: an International Multicentre (CONFIRM) registry, amongst patients without obstructive CAD, the extent of non-obstructive CAD provided additional prognostic information for MACE beyond traditional risk factors.^14,15^ Analysis of the Incident Coronary Syndromes Identified by Computed Tomography (ICONIC) study found that, although acute coronary syndromes (ACSs) are typically associated with stenotic coronary lesions, precursors of culprit lesions were commonly non-obstructive and high-risk plaque characteristics, plaque composition, and cross-sectional plaque burden as assessed by CCTA that were predictive of ACS, independent of initial stenosis severity.^9^

The International Study of Comparative Health Effectiveness with Medical and Invasive Approaches (ISCHEMIA) randomized a very different patient population compared with the aforementioned lower risk studies in which the minority of patients had obstructive CAD and even fewer had inducible ischaemia on stress testing.^9,10,12,15–17^ In ISCHEMIA, all enrolled patients had moderate or severe myocardial ischaemia and CCTA was used to ensure the exclusion of left main disease and the presence of obstructive CAD before randomization, renal function permitting. As a result, of the 3913 participants who underwent CCTA, 79.0% had multivessel CAD and 86.8% had left anterior descending (LAD) disease as defined by ≥50% stenosis.^18^ In the randomized ISCHEMIA cohort, greater extent and severity of obstructive CAD were associated with increased risk of all-cause mortality and MI.^19^ However, investigation of the use of additional, quantitative CCTA-derived measures of atherosclerosis severity for risk stratification in such a highly diseased, high-risk population is currently lacking.

The current study investigated the incremental clinical value of coronary plaque quantification in the highly diseased ISCHEMIA population, who were treated with guideline-directed medical therapy (GDMT) and, for participants randomized to the invasive strategy, guideline-directed revascularization. The primary and secondary aims of this analysis were to determine whether CCTA-derived atherosclerotic plaque characteristics were independently associated with MI or cardiovascular death and with the composite trial primary MACE endpoint, respectively. Furthermore, we evaluated the additional prognostic value beyond clinical characteristics and CCTA stenosis severity for the prediction of MACE at varying time points during follow-up.

## Methods

The present study included participants without prior coronary artery bypass grafting (CABG) from the ISCHEMIA trial with a core lab–evaluable CCTA.^18–21^ In brief, ISCHEMIA included a highly diseased, high-risk population of patients with chronic coronary disease and moderate or severe ischaemia on stress imaging tests or severe ischaemia on non-imaging exercise tolerance tests. Patients with recent ACS, New York Heart Association class III–IV heart failure, ejection fraction < 35%, and severe or unstable angina were excluded. Participants were randomized to an initial invasive strategy including routine cardiac catheterization with revascularization when feasible plus GDMT or an initial conservative strategy of GDMT alone, with coronary angiography and revascularization reserved for refractory symptoms or a suspected acute ischaemic event. Those with left main stenosis ≥ 50% or no lesion with ≥50% stenosis by baseline CCTA were excluded from randomization (*n* = 1652). Stress tests were interpreted at dedicated core laboratories according to published guidelines,^19^ blinded to coronary anatomy and clinical characteristics.

Upon randomization, most participants underwent core lab–interpreted CCTA (*n* = 3783), the results of which were blinded to the enrolling sites. Randomized participants who underwent a prior CCTA within 1 year of enrolment in the absence of a study CCTA were also included in this analysis (*n* = 130), resulting in a total of 3913 (75.6%) patients undergoing CCTA. A total of 1266 participants did not undergo CCTA due to kidney impairment (estimated glomerular filtration rate (eGFR) < 60 mL/min) or because of known coronary anatomy. Of the 3913 randomized participants who underwent CCTA, 87 with a history of CABG were excluded from the current analysis. Of the 3826 participants without prior CABG, 65 patients had no CCTA image data available, and 50 participants were excluded due to missing CCTA vessel territories (separate ostia of LAD and circumflex artery, no left main) by atherosclerosis imaging quantitative computed tomography (AI-QCT), resulting in a final study population of 3711 patients (see Supplementary data online, *Figure S1*).

### Atherosclerosis imaging quantitative computed tomography analysis

For this report, CCTA interpretation in this study was performed using a Food and Drug Administration–cleared software service (AI-QCT; Cleerly, Inc, Denver, CO, USA) that performs semi-automated analysis of CCTA using a series of validated convolutional neural network models (including VGG19 network, 3D U-Net, and VGG Network Variant) for image quality assessment, coronary segmentation and labelling, lumen wall evaluation and vessel contour determination, and plaque characterization. Training, testing, and outputs of the algorithms have been previously reported.^22,23^

Coronary segments with a diameter ≥ 1.5 mm were included in the analysis using the modified 18-segment society of cardiovascular computed tomography model.^24^ Each segment was evaluated for the presence or absence of coronary atherosclerosis, defined as any tissue structure > 1 mm^2^ within the coronary artery wall that was differentiated from the surrounding epicardial tissue, epicardial fat, or the vessel lumen itself. The AI-QCT diameter stenosis is calculated using an interpolated reference diameter at the site of stenosis, which is different than the proximal reference diameter standard for visual interpretation and traditional quantitative coronary angiographic stenosis determination. In detail, per cent diameter stenosis within a segment was represented by 1 minus the ratio of the lumen diameter at the site of maximal obstruction divided by the estimated normal lumen diameter at this site by interpolation of the normal proximal and normal distal reference vessel ×100. This method for determining stenosis severity has been previously reported to correlate better with invasively determined fractional flow reserve than traditional quantitative methods using a proximal reference.^22^ Quantitative atherosclerosis characterization was performed for every coronary artery and its branches using the automated web-based software, resulting in the AI-QCT parameters. The coronary arteries were evaluated for the presence and number of atherosclerotic lesions, which was defined as any structure larger than 1 mm^2^ within the coronary artery wall that was differentiated from the surrounding epicardial tissue, epicardial fat, or the vessel lumen itself. Total plaque volume (cubic millimetre) was defined as the sum of all coronary plaque volume. Plaque volume was further classified using Hounsfield unit (HU) ranges with non-calcified plaque volume defined as 30–350 HU, low-density non-calcified plaque volume defined as plaques < 30 HU, and calcified plaque defined as >350 HU. Arterial remodelling was calculated by examining the outer vessel wall diameter at the lesion divided by the normal vessel reference diameter, with positive remodelling defined as a remodelling ratio ≥ 1.5, given the high prevalence of positive remodelling ≥ 1.1 and CAD in this study population.^25^ Per the software design, high-risk plaque was defined as a coronary lesion with simultaneous presence of low-density non-calcified plaque volume and positive remodelling.^10^ Plaque diffuseness was calculated as the number of lesions with ≥30% diameter stenosis. For the univariable and multivariable models, lumen volume, defined as total lumen of the coronary segments included in the analysis, was divided by vessel length to calculate average lumen area across the coronary tree. In the case of impaired image quality due to motion, beam hardening, poor opacification, or other artefacts, only the uninterpretable part of the coronary vessel was excluded from the AI-QCT analysis.

### Study outcomes

The primary clinical outcome for this analysis was a composite outcome of cardiovascular death or MI. The secondary outcome for this analysis was the trial five-component primary endpoint (cardiovascular death, MI, or hospitalization for unstable angina, heart failure, or resuscitated cardiac arrest). Endpoint definitions have been previously published.^21^

### Statistical analysis

The outcome analysis was performed using Cox proportional hazards regression modelling with assigned management strategy incorporated as a stratum variable given violation of the underlying proportional hazards assumption of the model.^20^ In this analysis, death from non-cardiovascular causes was considered a competing risk. To characterize associations of study outcomes with covariates of interest, we reported cause-specific hazard ratios (HRs) estimated from Cox regression modelling. In univariable analysis, we estimated cause-specific HRs for AI-QCT stenosis severity (number of diseased vessels with obstructive stenosis ≥ 50%) and CCTA-derived atherosclerotic plaque variables that were selected based on prior literature, variability in the ISCHEMIA population, data availability, and correlations amongst the plaque variables to avoid collinearity (see Supplementary data online, *Figure S2*). The following plaque variables were selected for model inclusion: total plaque volume (cubic millimetre), low-density non-calcified plaque volume (cubic millimetre), number of high-risk plaques (cubic millimetre), proximal non-calcified plaque volume (cubic millimetre), diffuseness (number of lesions ≥ 30% stenosis), average lumen area (square millimetre), and remodelling index ≥ 1.5 (yes/no). To mitigate the potential effect of outlying observations, we applied a square root transformation to plaque variables that exhibited skewed distributions (total plaque volume, low-density non-calcified plaque volume, and proximal non-calcified plaque volume). Continuously measured variables were scaled to represent an increase from the 25th to 75th percentile, herein ‘interquartile increase’. Hazard ratios and *P*-values from the Wald test were reported.

In multivariable Cox regression analysis, we estimated cause-specific HRs from three models to explore the sequentially added prognostic value of AI-QCT stenosis severity and the atherosclerotic plaque variables. Of the 3711 study participants, 3673 (99%) had complete clinical risk factor data available for multivariable analysis. In Model 1, we included only clinical variables, namely, age; sex; diabetes (yes/no); hypertension (yes/no); smoking (current/former/never); eGFR (mL/min/1.73 m^2^); ejection fraction; known coronary heart disease (CHD; yes/no), defined as prior MI or percutaneous coronary intervention; and high-intensity statin therapy (yes/no). Model 2 included the clinical variables plus AI-QCT-derived number of diseased vessels (defined as vessels with obstructive stenosis ≥ 50%), and Model 3 included clinical variables plus AI-QCT-derived number of diseased vessels plus atherosclerotic plaque variables. We compared nested models using the likelihood ratio test. Using Cox regression modelling, we evaluated the predictive performance of the models in the presence of the competing risk of death from non-cardiovascular causes with a cause-specific approach, which combines the cause-specific Cox models for both the event of interest and the competing event.^26^ To evaluate predictive accuracy and discrimination, we estimated the time-dependent Brier score, continuous net reclassification improvement (NRI), and the time-dependent area under the curve (AUC), respectively, at 6 months, 2 years, and 4 years.^27,28^ The time-dependent Brier score is a summary of predictive accuracy that simultaneously measures both calibration and discrimination. For a given time point, the Brier score is computed as the sum of the squared errors between the observed event status and estimated survival. For NRI, methods from Pencina *et al*.^29^ were adapted to estimate the net reclassification index in a competing risk setting. Differences in performance measures between models were assessed using methods in Blanche *et al*.^28,30^ To compare AUCs between competing models, the *P*-values were obtained from Wald tests adapted to the setting of right censored survival data with competing risks.^30^ In this exploratory analysis, performance measures were computed within-sample and may be interpreted as an upper bound for the true predictive performance of the model. A higher AUC and lower Brier score each indicate a better model. Analyses were repeated for the five-component composite trial endpoint which was used as a secondary outcome in this study. Two sensitivity analyses were performed for the primary outcome: restricted to patients in the conservative strategy and including all patients with CCTA available, including those with prior CABG. Finally, with the aim to exclude peri-procedural events, an additional sensitivity analysis was performed restricted to the outcome of spontaneous MI.

Descriptive data are presented as median with interquartile range (IQR) for continuously measured data. Categorical variables are expressed as absolute numbers and percentages. We used a non-parametric estimator of the cumulative incidence function to estimate the cumulative incidence stratified by previously reported plaque stages based on total plaque volume and tertiles based on lumen volume.^31,32^ Differences in the cumulative incidence by stages and tertiles were assessed with the Fine–Gray method. All statistical analyses were performed using R software version 4.2.1 (R Foundation, Vienna, Austria) and SAS software version 9.4 (SAS Institute Inc, Cary, NC, USA). Evaluation of model predictive performance was conducted using the R package riskRegression.^33^

## Results

### Patient population

**Figure 1:**
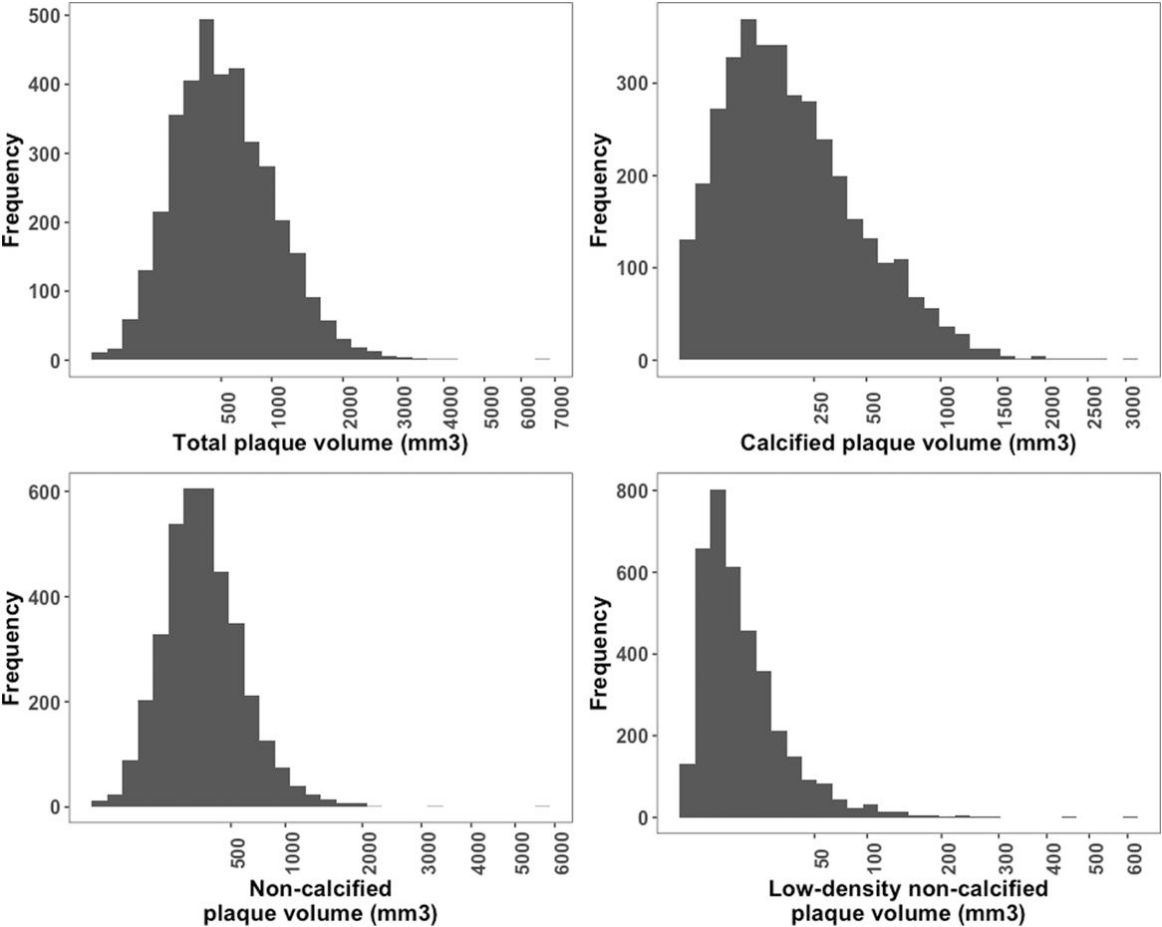
Distribution of plaque volumes in ISCHEMIA. Shown are histograms of the different plaque components in the ISCHEMIA trial population (*n* = 3711). Plaque volumes (*x*-axis) were transformed using a square root transformation

The study population (*n* = 3711) had a mean age of 64 [57–70] years, 2930 (79%) were male, 496 (13%) patients were active smokers, 1507 (41%) had diabetes, and 2575 (70%) had hypertension (*Table 1*). Median TPV was 494 [274–832] mm^3^, median NCPV was 292 [172–467] mm^3^, and median calcified plaque volume was 138 [45–328] mm^3^ (*Table 1*; *Figure 1*). Further baseline and CCTA characteristics are shown in *Table 1*. As quantitatively assessed using AI-QCT, 2865 (77%) patients had at least one vessel with ≥50% stenosis, of whom 914 (25% of study population) had two-vessel disease and 506 (14% of study population) patients had three-vessel or left main disease, which is consistent with the different definition of stenosis by AI-QCT compared with visual assessment and quantitative coronary angiography (QCA; see Supplementary data online, *Table S1*). The CCTA image quality was assessed 3 or higher on a 1–5 Likert scale in 2948 (79.4%) patients (see Supplementary data online, *Table S2*), and a total of 3.1% of the total vessel length was excluded due to artefacts. The median follow-up of the participants in this study was 3.3 [2.2–4.4] years. During this follow-up, 374 participants experienced cardiovascular death or MI, the primary outcome for this analysis, and there were 51 competing non-cardiovascular deaths.

**Table 1:** Baseline patient and quantitative coronary computed tomography angiography characteristics (*n* = 3711)

Conservative treatment strategy	1860 (50%)
Male sex	2930 (79%)
Age, years	64 [57–70]
Race	
White	2374 (65%)
Black	150 (4%)
Asian	1123 (31%)
Other or multiple race groups	31 (1%)
Ethnicity	
Not Hispanic or Latino	2897 (84%)
Hispanic or Latino	564 (16%)
Active smoking	496 (13%)
Diabetes	1507 (41%)
Hypertension	2575 (70%)
Family history of premature CHD	885 (27%)
Known CHD (prior MI or PCI)	682 (18%)
Left ventricular ejection fraction, %	60 [56–65]
eGFR, mL/min/1.73 m^2^	85 [73–101]
Coronary segment involvement score (core lab)	12 [10–13]
Total vessel volume, mm^3^	2496 [1944–3147]
Average lumen area, mm^2^	4 [3–4]
Total plaque volume, mm^3^	494 [274–832]
Per cent atheroma volume, %	20 [13–30]
Calcified plaque volume, mm^3^	138 [45–328]
Per cent calcified plaque volume, %	6 [2–12]
Non-calcified plaque volume, mm^3^	292 [172–467]
Per cent non-calcified plaque volume, %	12 [8–17]
Low-density non-calcified plaque volume, mm^3^	6 [2–16]
Presence of remodelling index > 1.5	1439 (39%)
Number of high-risk plaques (LD-NCPV > 0.5 mm^3^ and RI ≥ 1.1)	1 [0–3]
Number of lesions with ≥ 30% stenosis per vessel	4 [2–6]
Presence of at least one plaque in LM or proximal LAD	3538 (95%)
Presence of at least one proximal plaque	3683 (99%)
Proximal non-calcified plaque volume, mm^3^	197 [118–312]
Left main stenosis ≥ 50%	12 (0%)
Follow-up duration, years	3.3 [2.2–4.4]

Median [Q1–Q3]; *n* (%). Since median values were reported, the sum of non-calcified and calcified plaque volume did not exactly match the total plaque volume.

CHD, coronary heart disease; MI, myocardial infarction; PCI, percutaneous coronary intervention; eGFR, estimated glomerular filtration rate; LD-NCPV, low-density non-calcified plaque volume; RI, remodelling index; LM, left main coronary artery; LAD, left anterior descending coronary artery.

### Atherosclerosis imaging quantitative computed tomography–determined coronary computed tomography angiography characteristics and risk of outcomes

**Figure 2:**
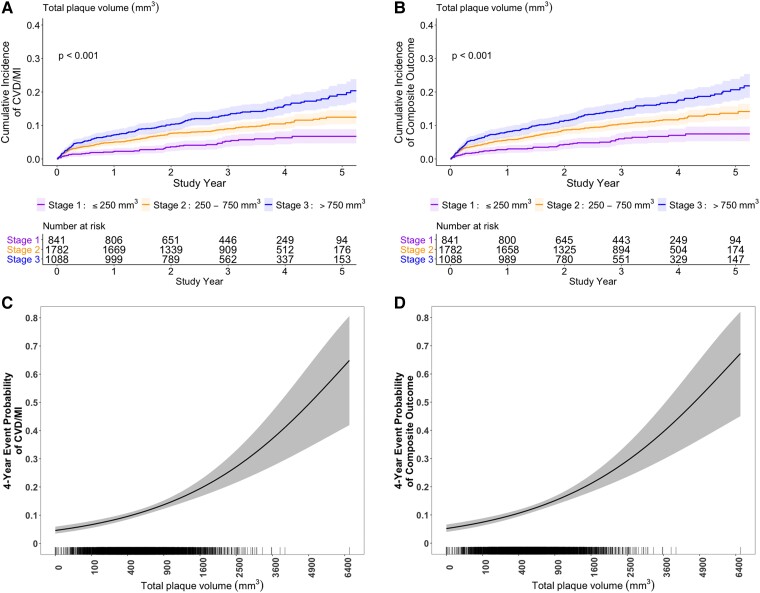
Relation between total plaque volume and cardiovascular death or myocardial infarction and the secondary composite outcome. Upper panel: shown is the cumulative incidence of cardiovascular death or myocardial infarction (*A*) and the secondary composite outcome of cardiovascular death, myocardial infarction, or hospitalization for unstable angina, heart failure, or resuscitated cardiac arrest (*B*). Patients were categorized according to total plaque volume stages [Stage 1 (≤250 mm^3^); Stage 2 (>250–750 mm^3^); Stage 3 (>750 mm^3^)]. *P*-values are from the Fine–Gray test to account for the competing risk of non-cardiovascular death. Lower panel: 4-year event probability of cardiovascular death or myocardial infarction (*C*) and the secondary composite outcome of cardiovascular death, myocardial infarction, or hospitalization for unstable angina, heart failure, or resuscitated cardiac arrest (*D*), according to total plaque volume. The vertical bars along the horizontal axis show the distribution of total plaque volume amongst the study participants. Shading refers to the 95% confidence interval

In the univariable analysis, the number of vessels with obstructive stenosis, total plaque volume, low-density non-calcified plaque volume, high-risk plaque, proximal non-calcified plaque volume, positive remodelling, and plaque diffuseness were each associated with the primary outcome of cardiovascular death or MI (*Table 2*). The effect of total plaque volume on cumulative incidence and the associated event rate is shown in *Figure 2*.

**Table 2:** Univariable atherosclerosis imaging quantitative computed tomography characteristics and risk of outcomes

Variable	CV death or MI		Secondary composite outcome^a^	
	HR (95% CI)	*P*-value	HR (95% CI)	*P*-value
AI-QCT stenosis severity				
Number of diseased vessels (≥50% stenosis)		<.001		<.001
0VD	Reference		Reference	
1VD	1.04 (0.77–1.42)		1.10 (0.83–1.47)	
2VD	1.54 (1.13–2.10)		1.52 (1.13–2.04)	
3VD/LMD	2.18 (1.57–3.02)		2.14 (1.56–2.91)	
AI-QCT atherosclerosis and lumen parameters				
Total plaque volume, per IQR increase	1.60 (1.42–1.81)	<.001	1.59 (1.41–1.78)	<.001
Low-density non-calcified plaque volume, per IQR increase	1.15 (1.05–1.27)	.004	1.14 (1.04–1.25)	.005
Proximal non-calcified plaque volume, per IQR increase	1.25 (1.11–1.41)	<.001	1.24 (1.11–1.39)	<.001
Number of high-risk plaques, per IQR increase	1.32 (1.17–1.50)	<.001	1.31 (1.17–1.48)	<.001
Diffuseness (number of lesions ≥ 30% stenosis), per IQR increase	1.47 (1.32–1.65)	<.001	1.46 (1.32–1.63)	<.001
Average lumen area, per IQR increase	0.81 (0.71–0.93)	.003	0.85 (0.75–0.96)	.012
Presence of remodelling index ≥ 1.5	1.40 (1.14–1.71)	.001	1.36 (1.12–1.65)	.002

A square root transformation was applied to plaque variables that exhibited skewed distributions (total plaque volume, low-density non-calcified plaque volume, and proximal non-calcified plaque volume). Continuously measured variables were scaled to represent an IQR increase from the 25th to 75th percentile. For total plaque volume, low-density non-calcified plaque volume, and proximal non-calcified plaque volume, these increases were from 274 to 832 (Q1–Q3) mm^3^, from 2 to 16 (Q1–Q3) mm^3^, and from 118 to 312 (Q1–Q3) mm^3^, respectively.

CV death, cardiovascular death; MI, myocardial infarction; AI-QCT, atherosclerosis imaging quantitative computed tomography.

^a^The secondary outcome was a composite of cardiovascular death, myocardial infarction, or hospitalization for unstable angina, heart failure, or resuscitated cardiac arrest (ISCHEMIA trial primary endpoint).

Adjusted for clinical characteristics, the presence of one-vessel disease defined as ≥50% stenosis as determined with AI-QCT resulted in an adjusted hazard ratio (aHR) of 1.12 (95% CI 0.83–1.53) for the primary outcome of cardiovascular death or MI, two-vessel disease had an aHR of 1.57 (1.15–2.15), and three-vessel disease had an aHR of 2.17 (1.56–3.02; *Table 3*; *P* = .001) as compared with no vessels with ≥50% stenosis on AI-QCT. For the five-component composite secondary outcome, the aHR was 1.20 [95% confidence interval (CI) 0.90–1.60] for one-vessel disease, 1.57 (95% CI 1.16–2.11) for two-vessel disease, and 2.16 (95% CI 1.57–2.96) for three-vessel disease (*P* = .001; Supplementary data online, *Table S3*). After further addition of the AI-QCT atherosclerosis parameters, the number of diseased vessels was no longer an independent predictor for the primary outcome nor the five-component composite secondary outcome (*Table 4*; Supplementary data online, *Table S3*). Of the AI-QCT atherosclerosis parameters, total plaque volume was most strongly associated with the primary outcome [aHR 1.56 per IQR increase (559 mm^3^); 95% CI 1.25–1.97; *P* = .001] and five-component composite secondary outcome [aHR 1.49 per IQR increase (559 mm^3^); 95% CI 1.20–1.85; *P* = .001]. Average lumen area was inversely related to the primary outcome (aHR 0.72 per IQR increase; 95% CI 0.61–0.85; *P* = .001; Supplementary data online, *Figure S4*) and five-component composite secondary outcome (aHR 0.77 per IQR increase; 95% CI 0.66–0.89; *P* = .001), with smaller volumes conferring higher risk.

**Table 3:** Multivariable models with atherosclerosis imaging quantitative computed tomography characteristics for prediction of cardiovascular death or myocardial infarction

Variable	Model 1 Clinical characteristics		Model 2 Clinical + number of diseased vessels		Model 3 Clinical + number of diseased vessels + atherosclerosis quantification	
	HR (95% CI)	*P*-value	HR (95% CI)	*P*-value	HR (95% CI)	*P*-value
Clinical characteristics						
Age, per IQR increase	1.29 (1.11, 1.51)	.001	1.30 (1.11, 1.52)	.001	1.16 (0.98, 1.37)	.082
Female sex	0.91 (0.70, 1.19)	.508	1.00 (0.76, 1.32)	.985	1.06 (0.80, 1.39)	.703
Active smoking	1.07 (0.85, 1.34)	.431	1.08 (0.86, 1.36)	.312	1.02 (0.81, 1.28)	.643
Diabetes	1.38 (1.12, 1.69)	.002	1.32 (1.08, 1.63)	.008	1.30 (1.05, 1.60)	.015
Hypertension	1.81 (1.37, 2.38)	.001	1.77 (1.35, 2.33)	.001	1.78 (1.35, 2.35)	.001
Ejection fraction, per IQR increase	0.86 (0.77, 0.96)	.010	0.87 (0.78, 0.98)	.017	0.88 (0.78, 0.98)	.022
Prior MI or PCI	1.05 (0.82, 1.35)	.696	1.08 (0.84, 1.39)	.557	1.09 (0.84, 1.40)	.511
eGFR, per IQR increase	0.91 (0.78, 1.05)	.206	0.91 (0.79, 1.06)	.227	0.89 (0.77, 1.04)	.135
High-intensity statin at randomization	1.05 (0.85, 1.31)	.642	1.05 (0.84, 1.30)	.675	1.06 (0.85, 1.31)	.631
AI-QCT stenosis severity						
Number of diseased vessels				.001		.124
Less than 50% stenosis			Reference		Reference	
1VD			1.12 (0.83, 1.53)		0.98 (0.72, 1.35)	
2VD			1.57 (1.15, 2.15)		1.16 (0.82, 1.64)	
3VD			2.17 (1.56, 3.02)		1.45 (0.97, 2.15)	
AI-QCT atherosclerosis and vessel parameters						
Total plaque volume					1.56 (1.25, 1.97)	.001
Low-density non-calcified plaque volume					1.08 (0.92, 1.26)	.342
Number of high-risk plaques					1.14 (0.95, 1.36)	.161
Proximal non-calcified plaque volume					0.83 (0.67, 1.03)	.096
Diffuseness					1.02 (0.86, 1.20)	.847
Average lumen area					0.72 (0.61, 0.85)	.001
Remodelling index ≥ 1.5					0.93 (0.74, 1.17)	.542

Shown are multivariable Cox regression models including clinical characteristics (Model 1), clinical and AI-QCT stenosis parameters (Model 2), and a model with clinical, AI-QCT stenosis, and AI-QCT atherosclerosis quantification (Model 3). A square root transformation was applied to plaque variables that exhibited skewed distributions (total plaque volume, low-density non-calcified plaque volume, and proximal non-calcified plaque volume). Continuously measured variables were scaled to represent an interquartile increase from the 25th to 75th percentile. For total plaque volume, low-density non-calcified plaque volume, and proximal non-calcified plaque volume, these increases were 559 (Q1–Q3) mm^3^, 14 (Q1–Q3) mm^3^, and 194 (Q1–Q3) mm^3^, respectively. The interquartile increase was from 13 (Q1–Q3) years for age, 9% (Q1–Q3) for ejection fraction, and 28 (Q1–Q3) mL/min/1.73 m^2^ for eGFR.

AI-QCT, atherosclerosis imaging quantitative computed tomography.

**Table 4:** Performance of multivariable models with atherosclerosis imaging quantitative computed tomography characteristics for prediction of cardiovascular death or myocardial infarction

		Model 1	Model 2		Model 3		
		Clinical characteristics	Model 1 + number of diseased vessels	*P*-value vs. Model 1	Model 2 + atherosclerosis quantification	*P*-value vs. Model 1	*P*-value vs. Model 2
6 months	AUC (95% CI)	0.637 (0.592–0.682)	0.670 (0.627–0.714)	.010	0.688 (0.641–0.735)	.006	.234
	Brier score	0.0360	0.0358	.047	0.0354	.005	.016
	NRI	Reference	Vs. M1: 0.38 (0.15–0.56)		Vs. M1: 0.40 (0.23–0.60) Vs. M2: 0.28 (0.10–0.49)		
2 years	AUC (95% CI)	0.617 (0.582–0.652)	0.641 (0.607–0.676)	.024	0.660 (0.624–0.696)	.003	.080
	Brier score	0.0690	0.0686	.166	0.0675	.002	.002
	NRI	Reference	Vs. M1: 0.33 (0.13–0.45)		Vs. M1: 0.31 (0.22–0.49) Vs. M2: 0.24 (0.13–0.40)		
4 years	AUC (95% CI)	0.608 (0.574–0.643)	0.622 (0.587–0.658)	.205	0.654 (0.619–0.689)	.002	.004
	Brier score	0.0985	0.0982	.464	0.0959	.004	.001
	NRI	Reference	Vs. M1: 0.25 (0.11–0.37)		Vs. M1: 0.32 (0.23–0.49) Vs. M2: 0.26 (0.17–0.42)		

Shown are multivariable Cox regression models including clinical characteristics (Model 1), clinical and AI-QCT stenosis parameters (Model 2), and a model with clinical, AI-QCT stenosis, and AI-QCT atherosclerosis quantification (Model 3).

AI-QCT, atherosclerosis imaging quantitative computed tomography; AUC, area under the curve; NRI, net reclassification improvement; M1, Model 1; M2, Model 2.

### Prognostic value of atherosclerosis imaging quantitative computed tomography–determined atherosclerosis

**Figure 3:**
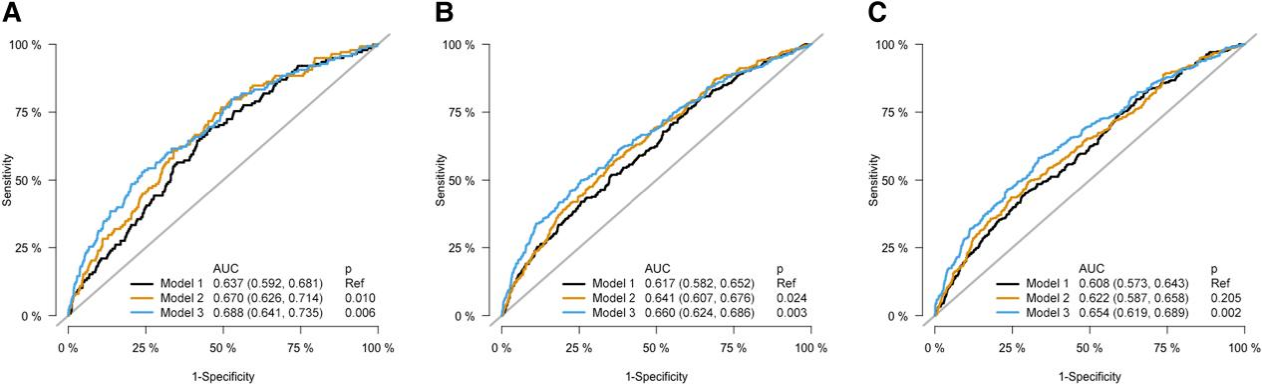
Prognostic value of different models for cardiovascular death or myocardial infarction. Discriminatory value for the primary outcome of cardiovascular death and myocardial infarction. Receiver operating characteristic curves of a model with clinical characteristics (Model 1), a model with clinical and atherosclerosis imaging quantitative computed tomography stenosis parameters (Model 2) and a model with clinical, atherosclerosis imaging quantitative computed tomography stenosis, and atherosclerosis imaging quantitative computed tomography atherosclerotic variables (Model 3). Areas under the curve were calculated at 6 months (*A*), 2 years (*B*), and 4 years (*C*) of follow-up. The 95% confidence interval is shown between brackets

Subsequently, we compared the prognostic information from the different sequential multivariable models (*Figure 3*; *Table 4*). At 6 months, the clinical model resulted in an AUC of 0.637 (95% CI 0.592–0.682), which increased to 0.670 (95% CI 0.627–0.714) with addition of AI-QCT-derived vessel involvement [ΔAUC 0.033; *P* = .010; NRI 0.38 (95% CI 0.15–0.56)]. Addition of plaque characteristics to the vessel involvement did not result in further discriminatory improvement of the model at 6 months [AUC 0.688 (95% CI 0.641–0.735); ΔAUC 0.018; *P* = .234; NRI 0.28 (95% CI 0.10–0.49)]. At the 2-year follow-up, the AUC of the clinical risk model increased from 0.617 (95% CI 0.582–0.652) to 0.641 (95% CI 0.607–0.676) after addition of AI-QCT-derived vessel involvement [ΔAUC 0.024; *P* = .024; NRI 0.33 (95% CI 0.13–0.45)]. Further addition of quantitative plaque characteristics to the model showed a non-significant increase in AUC [AUC 0.660 (95% CI 0.624–0.696); ΔAUC 0.019; *P* = .080; NRI 0.24 (95% CI 0.13–0.40)]. After 4 years of follow-up, the clinical risk model [AUC 0.608 (95% CI 0.574–0.643)] did not improve with addition of AI-QCT-derived vessel involvement [AUC 0.622 (95% CI 0.587–0.658); ΔAUC 0.014; *P* = .205; NRI 0.25 (95% CI 0.11–0.37)]. However, compared with the clinical model, further addition of quantitative plaque characteristics by AI-QCT improved the model performance [AUC 0.654 (95%CI 0.619–0.689); ΔAUC 0.046; *P* = .002; NRI 0.32 (95% CI 0.23–0.49)].

The results for the five-component composite secondary outcome were similar except that atherosclerosis variables did result in higher AUC at 6 months compared with the combination of clinical characteristics and the number of diseased vessels (see Supplementary data online, *Figure S5*; Supplementary data online, *Table S4*). A sensitivity analysis restricted to patients in the conservative strategy, although limited by a smaller sample size, found similar results but numerically higher AUCs for the outcome of cardiovascular death or MI (see Supplementary data online, *Table S5*). In this analysis, the AUCs of the model including number of diseased vessels and plaque characteristics were 0.688 (95% CI 0.614–0.762), 0.676 (95% CI 0.627–0.726), and 0.685 (95% CI 0.638–0.731), at 6 months, 2 years, and 4 years of follow-up, respectively. Another sensitivity analysis including patients with prior CABG was similar to the main analysis (see Supplementary data online, *Table S6*). The sensitivity analysis restricted to an outcome of spontaneous MI showed similar results as for the primary and secondary outcome (see Supplementary data online, *Table S7*). Finally, a sensitivity analysis with visual assessment of the number of diseased vessels also showed similar results (see Supplementary data online, *Table S8*).

## Discussion

In this post hoc analysis of the ISCHEMIA cohort CCTA data, we show that the number of diseased vessels and atherosclerotic burden were independently associated with increased risk of cardiovascular death or MI while coronary lumen area was inversely associated with risk. The features derived from quantitative CCTA provided modest improvement of discrimination and reclassification for prognosis when added to clinical characteristics. The current data suggest that the number of vessels with obstructive CAD as defined by AI-QCT using an interpolated reference diameter modestly improved discrimination of MACE in the near term up to 2 years, whereas atherosclerotic burden and high-risk plaque characteristics improved discrimination of MACE over and above the number of diseased vessels at 4 years. The results were similar when restricted to the conservative strategy group. To our knowledge, these data are the first showing the added prognostic impact of angiographic stenosis and quantitative measures of atherosclerosis derived from CCTA in such a high-risk population as ISCHEMIA, albeit the absolute AUC values and improvements in prognostic value were modest. These results, derived in a select population of patients with angiographically severe CAD and moderate or severe myocardial ischaemia, support the concept that measuring quantitative atherosclerotic plaque volume can enhance estimates of prognosis, even in patients with extensive CAD already on GDMT (*Structured Graphical Abstract*).

Angiographic CAD severity—whether assessed by invasive coronary angiography or by CCTA—is associated with MACE.^34–39^ Using CCTA core lab stenosis grading, our group previously reported a graded association between CAD severity (based on stenosis) and adverse outcomes.^19^ We evaluated the extent and severity of CAD in a subgroup of ISCHEMIA patients who had fully interpretable CCTAs for the Duke Prognostic Index (*n* = 2475, 48%). The most severe CAD subgroup had a higher risk of all-cause death (HR 2.27, 95% CI 1.37–3.75) and MI (HR 1.69, 95% CI 1.17–2.45) compared with the lowest risk subgroup, after adjustment for other clinical variables including ischaemia on stress testing. Importantly, the Duke Prognostic Index subgroups are categorizations that integrate a combination of non-obstructive and obstructive angiographic CAD. In the same study, ischaemia severity was not associated with the primary and major secondary endpoints. The entirety of these findings may reflect the inherent relationship between the burden of atherosclerosis and the likelihood of plaque instability causing fatal and nonfatal events, which is not necessarily determined by the grade of myocardial ischaemia. The current study adds to these findings that beyond CAD severity assessed by CCTA stenosis grading, quantitative atherosclerotic plaque characteristics can improve risk stratification for MACE, even amongst a highly enriched population of patients with at least moderate stress-induced ischaemia and severe angiographic CAD.

Numerous multicentre trials have demonstrated atherosclerosis to be a strong predictor of future MACEs in populations with low to moderate obstructive disease severity prevalence and plaque burden.^9–13^ It was previously unknown whether atherosclerotic features and burden remained associated with MACE in those with both high CAD severity and ischaemia. The ISCHEMIA trial has a unique selection of patients with obstructive CAD (99.9% with visual ≥50% stenosis), including 44% with CCTA core lab–determined three-vessel disease in the current analysis, in contrast to other large multicentre CCTA studies in which the percentage of patients with obstructive disease was much lower. In comparison, the PROMISE trial reported 14% of patients having obstructive disease (≥50% stenosis), with only 6% having either ≥70% stenosis or ≥50% left main disease.^10^ In SCOT-HEART, 26% of patients had obstructive disease (either ≥70% stenosis or ≥50% left main disease),^12^ as had 23% of the patients in CONFIRM (≥50% stenosis).^35^ Hence, clinical use of CCTA and guideline implementation have been predominantly focused on low-to-intermediate risk patients with relatively low atherosclerotic burden compared with the current study. In comparison with a recent cohort study using AI-QCT in symptomatic patients suspected of CAD, both median per cent atheroma volume and non-calcified plaque volume were four times higher in the current study (20% vs. 5% and 12% vs. 3%), underlining the high disease severity in the ISCHEMIA trial population.^40^ Leveraging data from 3711 patients who underwent a pre-randomization CCTA, we here show that atherosclerotic plaque features were independently associated with MACE during a 4-year follow-up in a highly diseased population.

After adjustment for covariates including other plaque characteristics, total plaque volume was the only atherosclerotic parameter that demonstrated a positive association with the composite of cardiovascular death or MI. Therefore, a staging system based on total plaque volume or burden, as recently described,^31,32^ could also be of important value for risk prognostication in a high-risk population. Approximately one-third of patients in the ISCHEMIA trial would be in the highest Stage 3 (severe plaque), who could remain at high residual risk after implementing GDMT with or without revascularization, and thus might benefit from additional interventions such as anti-inflammatory agents, antithrombotic agents, or other emerging therapies. Beyond total plaque volume, we found that proximal non-calcified plaque, plaque diffuseness, and high-risk plaque characteristics were associated with the primary and secondary outcomes; however, these associations were no longer found after adjustment for clinical covariates and other AI-QCT parameters. Adjusted for total plaque volume and other covariates, participants with greater average lumen area were at lower risk for MACE. Previous studies have found that lower lumen volumes were associated with abnormal fractional flow reserve and myocardial blood flow.^41–43^ Independent of plaque volume, participants with more compensatory mechanisms following severe atherosclerotic coronary disease resulting in increased lumen volume might subsequently be at lower risk of cardiovascular events, and in the case of plaque rupture with thrombosis, greater lumen volume may prevent coronary occlusion.

Although we found a modest association between quantitative plaque characteristics and MACE in this study, most of the prior CCTA studies in cohorts with lower CAD severity reported stronger associations. Though plaque parameters have consistently been shown to predict adverse outcomes, different studies have identified different plaque parameters as most important. In PROMISE, the presence of high-risk plaque was associated with a higher MACE rate after adjustment for atherosclerotic cardiovascular disease (ASCVD) risk score and significant stenosis (aHR 1.72; 95% CI, 1.13–2.62); and this led to a significant continuous NRI (0.34; 95% CI 0.02–0.51).^10^ Taron *et al*.^44^ reported that composite events were independently predicted by ASCVD risk with the presence of ≥2 high-risk plaque features (HR 2.40, 95% CI 1.25–4.58; *P* = .008). In SCOT-HEART, low-attenuation plaque burden was the strongest predictor of MI (aHR 1.60, 95% CI 1.10–2.34; *P* = .014), irrespective of cardiovascular risk score, coronary artery calcium score, or coronary artery stenosis. In CONFIRM, when evaluating patients without significant stenosis, the magnitude of risk was higher for CCTA-defined atherosclerotic extent; the aHR for segment involvement score (SIS) > 5 was 3.4 (95% CI 2.3–4.9).^14^ It is important to recognize that in the overall population, most cardiac events occur in people without obstructive stenosis, and those events are most frequently caused by rupture of vulnerable plaques. Therefore, the presence of high-risk plaque characteristics provides important prognostic value for MACE, as shown in previous CCTA studies with a lower disease prevalence.^9–13^ However, in ISCHEMIA, the high prevalence of high-risk plaques as defined by CCTA and the highly diseased coronary arteries of the study population might have reduced the discriminatory value of having vulnerable plaque. Hence, the observed prognostic value of high-risk plaque for MACE in the univariable analysis may have been attenuated by the inclusion of overall burden of disease (total plaque volume and average lumen area) in the adjusted multivariable analysis.

In the current study, we identified potentially time-dependent effects on the relationship between angiographic stenosis, atherosclerosis, and outcomes. In the first 6 months of follow-up, atherosclerosis parameters did not provide discriminatory value beyond AI-QCT-determined stenosis. This may imply that CAD severity in terms of stenosis might be the most important predictor of prognosis in the near term. However, for the longer-term—especially at 4 years of follow-up—addition of atherosclerotic quantification and high-risk plaque features to the risk model seemed to provide stronger discriminatory benefit, whereas the stenosis severity seemed less important. It is therefore tempting to speculate that, in patients with moderate or severe ischaemia, the degree of coronary stenosis and plaque volume reflects the near-term risk of acute ischaemic events. On the contrary, atherosclerotic burden and high-risk plaque characteristics may be a marker for longer-term events, similar to what was observed in studies in patients with lower frequencies of obstructive disease and ASCVD risk, such as the SCOT-HEART and CONFIRM.^10,12,35^ Nevertheless, differences in AUCs were overall relatively small in this study, requiring caution while interpreting these results. Future longitudinal studies evaluating a greater distribution of angiographic and ischaemic CAD may be informative in this regard.

### Limitations

There was a relatively short duration of follow-up with a median of 3.3 years, which limits the current analysis. Extended follow-up of the ISCHEMIA trial cohort for mortality is currently ongoing. The ISCHEMIA population was enriched for patients with CT angiographic stenosis but no left main stenosis. Coronary computed tomography angiography was not performed in all participants, mostly because of renal dysfunction. These patients, as well as patients with prior CABG, were excluded from the primary analysis. The number of participants with obstructive disease according the AI-QCT interpretation was lower (77.2%) compared with the original ISCHEMIA CT core lab interpretation (99.9%) and angiographic core laboratory interpretation (92.2%), which can be explained by a different stenosis definition by AI-QCT compared with these techniques. Nevertheless, both the core lab interpretation in ISCHEMIA and the AI-QCT algorithm have previously illustrated high agreement with invasive QCA.^22,45^ While some CCTA studies had uninterpretable segments, the automated AI-QCT algorithm found fewer uninterpretable scans than true expert core lab consensus (1.2% vs. 23.6%), consistent with prior reports.^22^ The plaque volumes reported in the current study may not be comparable with other studies due to between-platform differences. For most cardiovascular risk factors included in the multivariable model, only presence, but not severity, was assessed. Strong emphasis was placed on getting ISCHEMIA participants on GDMT, which may have altered the prognostic impact of some of the plaque features defined on the baseline CCTA. Finally, ISCHEMIA was a strategy trial, in which cardiac catheterization and revascularization were permitted in participants in the initial conservative strategy who had refractory angina or an acute ischaemic event in follow-up. Invasive procedures in conservative strategy participants could have influenced the natural history of study participants and thus could have affected our findings.

## Conclusions

Quantitative CCTA parameters of stenosis severity and atherosclerotic burden were independently associated with cardiovascular death and MI in a patient population with advanced CAD and moderate or severe myocardial ischaemia. However, these parameters offered only modest improvement in prognostic value beyond clinical risk characteristics.

## Supplementary Material

ehae471_Supplementary_Data

## Declarations

### Data Availability

The data underlying this article will be shared on reasonable request to the corresponding author.

### Pre-registered Clinical Trial Number

NCT01471522 (https://clinicaltrials.gov/ct2/show/NCT01471522).
