# Supplementary material for: Atherosclerosis quantification and cardiovascular risk: the ISCHEMIA trial
Source: Eur Heart J. 2024 Aug 5;45(36):3735–47. doi: 10.1093/eurheartj/ehae471 (PMC11439108; doi:10.1093/eurheartj/ehae471)
Supplement: ehae471_Supplementary_Data [file ehae471_supplementary_data.docx]

**ONLINE-ONLY SUPPLEMENTALS**

**Association Between Atherosclerosis Quantification and Characterization and Risk of Major Adverse Cardiovascular Events: The ISCHEMIA Trial**

**Table of Contents**

[Supplementary Methods 2](#_Toc169711045)

[Supplementary Figure 1. Flowchart of patient inclusion 3](#_Toc169711046)

[Supplementary Figure 2. Pairwise age and sex-adjusted Spearman correlations for the AI-QCT variables 4](#_Toc169711047)

[Supplementary Figure 3. Distribution of average lumen area in ISCHEMIA 5](#_Toc169711048)

[Supplementary Figure 4. Relation between average lumen area and CV death or MI, and the secondary composite outcome 6](#_Toc169711049)

[Supplementary Figure 5. Prognostic value of different models for the 5-component secondary outcome of CV death, MI or hospitalization for unstable angina, heart failure or resuscitated cardiac arrest 7](#_Toc169711050)

[Supplementary Table 1. Interpretability and grading of AI-QCT and core lab visual assessment 8](#_Toc169711051)

[Supplementary Table 2. CCTA image quality in the study population 9](#_Toc169711052)

[Supplementary Table 3. Multivariable models with AI-QCT characteristics for prediction of the 5-component secondary outcome of CV death, MI or hospitalization for unstable angina, heart failure or resuscitated cardiac arrest 10](#_Toc169711053)

[Supplementary Table 4. Performance of multivariable models with AI-QCT characteristics for the 5-component secondary outcome of CV death, MI or hospitalization for unstable angina, heart failure or resuscitated cardiac arrest 12](#_Toc169711054)

[Supplementary Table 5. Sensitivity analysis: Performance of multivariable models with AI-QCT characteristics for prediction of the primary outcome of CV death or MI restricted to the conservative treatment group (n=1,839) 13](#_Toc169711055)

[Supplementary Table 6. Sensitivity analysis: Performance of multivariable models with AI-QCT characteristics for prediction of the primary outcome of CV death or MI including patients with prior CABG (n=3,759) 14](#_Toc169711056)

[Supplementary Table 7. Sensitivity analysis: Performance of multivariable models with AI-QCT characteristics for prediction of spontaneous MI 15](#_Toc169711057)

[Supplementary Table 8. Sensitivity analysis: Performance of multivariable models with visual stenosis assessment of number of diseased vessels. 16](#_Toc169711058)

[ISCHEMIA Committee, CCC, Trial-Related Personnel 17](#_Toc169711059)

[ISCHEMIA Site Investigators and Coordinators 26](#_Toc169711060)

# Supplementary Methods

**Core laboratory CCTA analysis**

As previously reported, segmental interpretation of the CCTA was carried out according to Society of Cardiovascular Computed Tomography (SCCT) guidelines, with each segment coded as demonstrating no stenosis, 1–24% stenosis, 25–49% stenosis, 50–69% stenosis or 70–100% stenosis, if the segment was interpretable based on visual assessment (“visual stenosis”).^18^ The segment involvement score was calculated as the total number of coronary artery segments exhibiting plaque of any severity, as previously described.^19^ CCTA image quality was recorded based on assessment of overall image noise, presence of motion artifacts, poor contrast, misregistration, adequacy of field of view, calcium affecting segmental analysis or coronary stents, among other factors. All CCTA segmental assessments were determined by consensus of at least 2 independent readers, and all cases were also reviewed for left main disease by a third reader. CCTA was considered not evaluable for number of diseased vessels if certain large and proximal segments designated *a priori* by the core laboratory could not be interpreted for stenosis severity, for example, the mid RCA, due to cardiac motion artifact.

# Supplementary Figure 1. Flowchart of patient inclusion

**
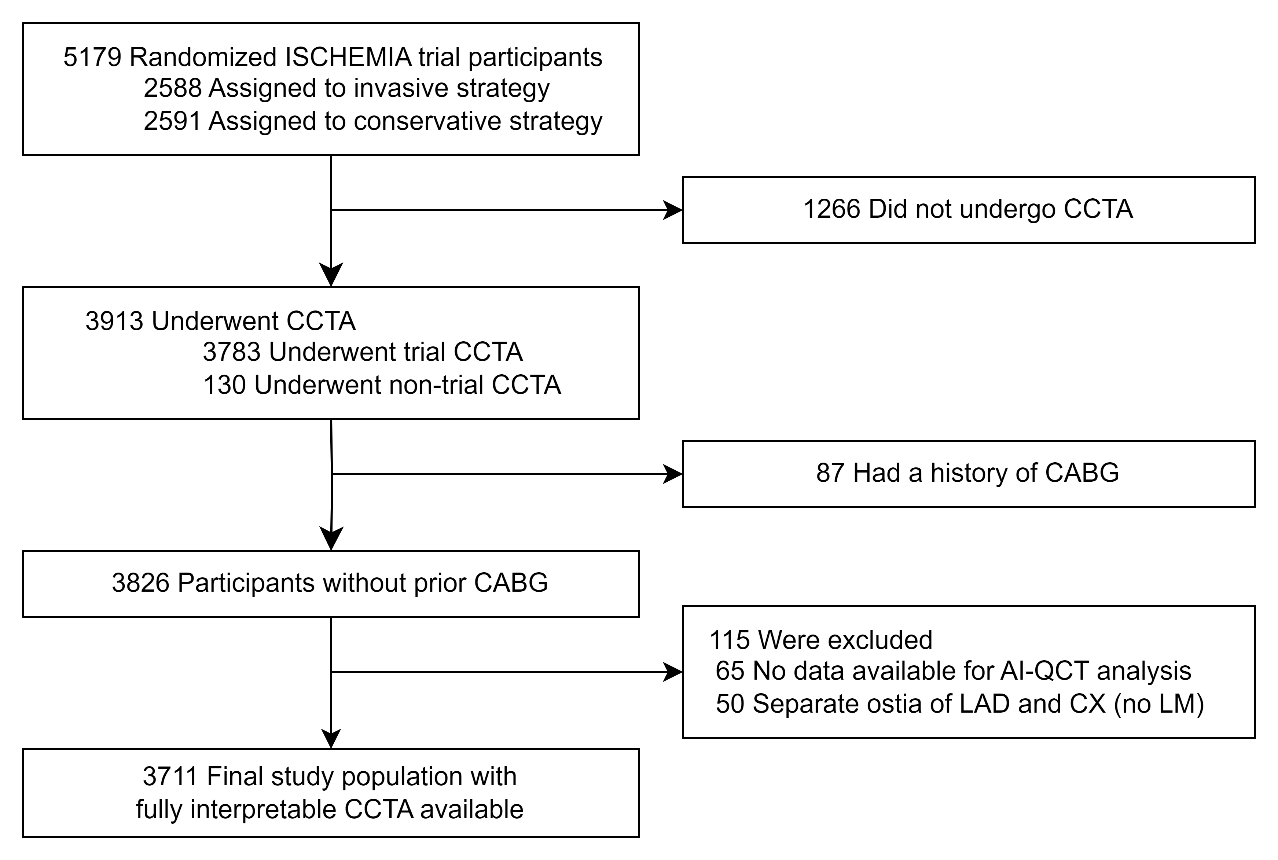
**

Flowchart of patient inclusion. Patients with a history of CABG and without LM were excluded. ISCHEMIA, The International Study of Comparative Health Effectiveness With Medical and Invasive Approaches; CCTA, coronary CT angiography; CABG, coronary artery bypass grafting; AI-QCT, Atherosclerosis Imaging-Quantitative Computed Tomography analysis. LAD, left anterior descending coronary artery; CX, circumflex coronary artery; LM, left main coronary artery.

# Supplementary Figure 2. Pairwise age and sex-adjusted Spearman correlations for the AI-QCT variables

**
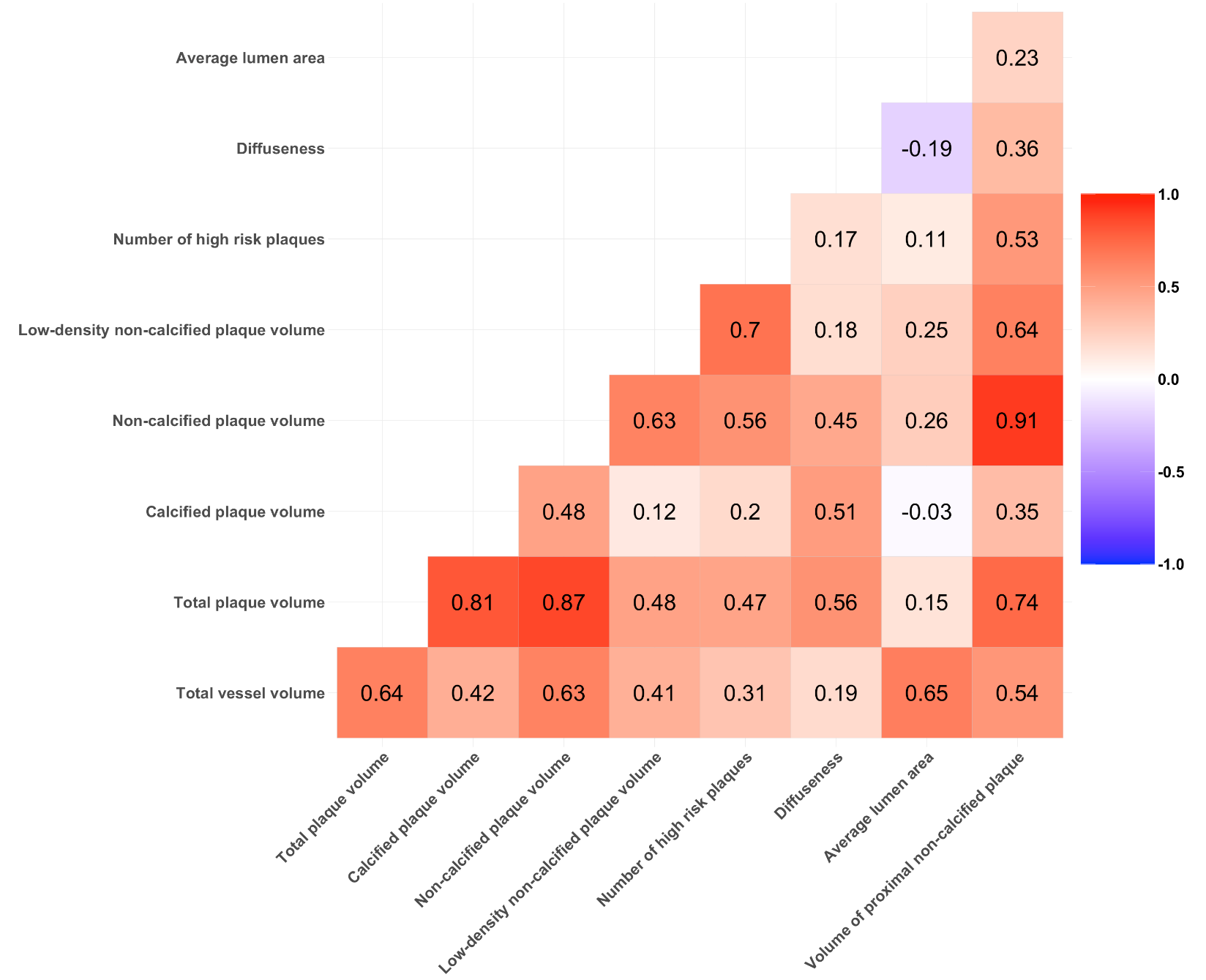
**

Age and sex-adjusted Spearman correlation coefficients among the continuously measured plaque variables which were used to select the variables in the Cox regression models.

# Supplementary Figure 3. Distribution of average lumen area in ISCHEMIA

**
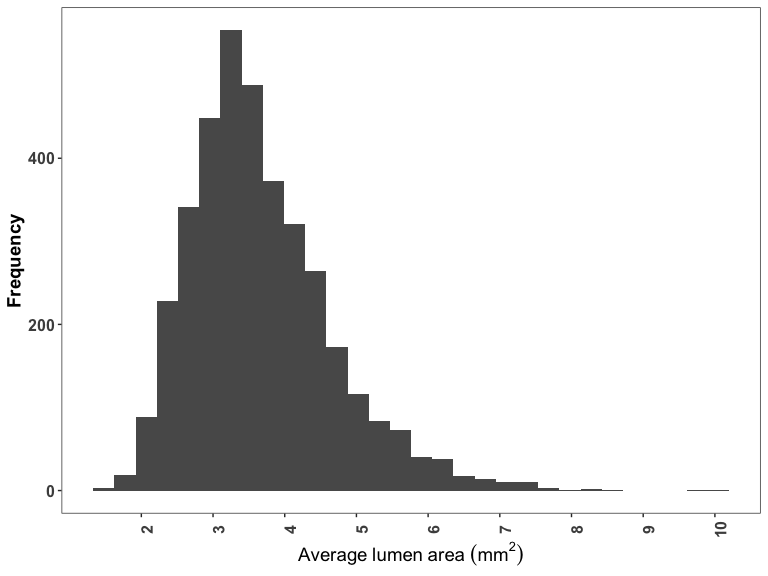
**

Shown is the histogram of average lumen area in the ISCHEMIA trial population (n=3,711). To calculate average lumen area across the coronary tree, the total lumen of the coronary segments included in the analysis was divided by the total vessel length.

# Supplementary Figure 4. Relation between average lumen area and CV death or MI, and the secondary composite outcome

**
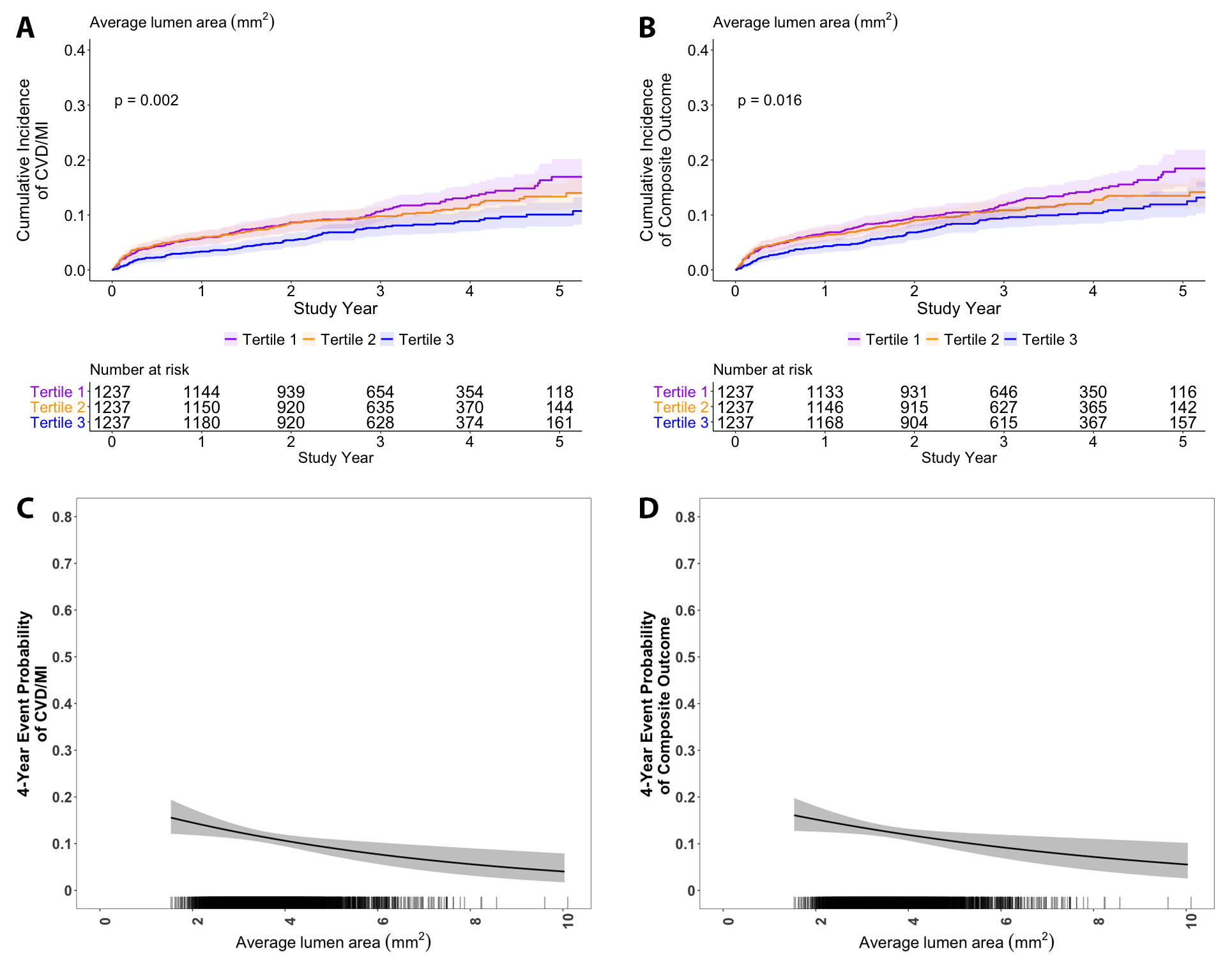
**

Upper panel: shown is the cumulative incidence of cardiovascular death or myocardial infarction (A) and the secondary composite outcome of cardiovascular death, myocardial infarction, or hospitalization for unstable angina, heart failure, or resuscitated cardiac arrest (B). Patients were categorized according to tertiles of average lumen area (Tertile 1 [≤3.2 mm^2^]; Tertile 2 [3.2-3.9 mm^2^]; Tertile 3 [>3.9 mm^2^]). P-values are from the Fine-Gray test to account for the competing risk of non-cardiovascular related death. Lower panel: 4-year event probability of cardiovascular death or myocardial infarction (C) and the secondary composite outcome of cardiovascular death, myocardial infarction, or hospitalization for unstable angina, heart failure, or resuscitated cardiac arrest (D), according to average lumen area. The vertical bars along the horizontal axis show the distribution of average lumen area among the study participants. Shading refers to the 95 percent confidence interval. CV death, cardiovascular death; MI, myocardial infarction.

# Supplementary Figure 5. Prognostic value of different models for the 5-component secondary outcome of CV death, MI or hospitalization for unstable angina, heart failure or resuscitated cardiac arrest


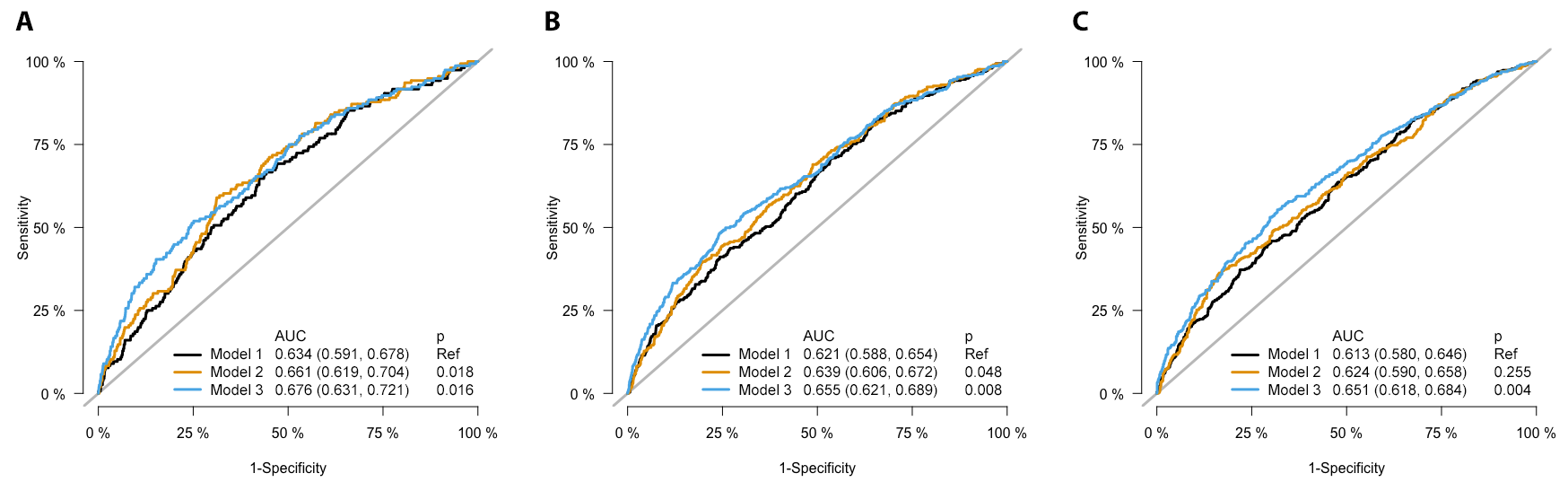


Discriminatory value for the secondary outcome (ISCHEMIA trial primary endpoint: cardiovascular death, myocardial infarction, or hospitalization for unstable angina, heart failure, or resuscitated cardiac arrest). There were a total of 417 secondary outcome events. Receiver operating characteristic curves of a model with clinical characteristics (Model 1), a model with clinical and AI-QCT stenosis parameters (Model 2) and a model with clinical, AI-QCT stenosis and AI-QCT atherosclerotic variables (Model 3). AUCs were calculated at 6 months (A), 2 years (B) and 4 years (C) of follow-up. The 95% confidence interval is shown between brackets. AUC, area under the curve; CV death, cardiovascular death; MI, myocardial infarction.

# Supplementary Table 1. Interpretability and grading of AI-QCT and core lab visual assessment

|  | **AI-QCT** | **Visual stenosis grading** | **p-value** |
| --- | --- | --- | --- |
| *Interpretability of CCTA* |  |  |  |
| Interpretable for number of diseased vessels | 3,711/3,761 (98.8%) | 2,874/3,761 (76.4%) | <0.001 |
| *Number of diseased vessels per patient (≥50% stenosis)* |  |  | <0.001 |
| Less than 50% stenosis | 846 (22.8%) | 3 (0.1%) |  |
| 1VD | 1,445 (39.0%) | 689 (24.0%) |  |
| 2VD | 914 (24.6%) | 913 (31.8%) |  |
| 3VD/LMD | 506 (13.6%) | 1,269 (44.2%) |  |
| *Total number of diseased vessels (≥50% stenosis)* | 4,839/14,992 (32.3%) | 7,634/14,047 (54.4%) | <0.001 |

AI-QCT, Atherosclerosis Imaging-Quantitative Computed Tomography analysis; CCTA, coronary CT angiography; AUC, area under the curve.

# Supplementary Table 2. CCTA image quality in the study population

| **Image quality Likert score** | **Study population** (n=3,711) |
| --- | --- |
| 1 – Poor | 95 (2.6%) |
| 2 – Fair | 668 (18.0%) |
| 3 – Good | 1,155 (31.1%) |
| 4 – Very good | 1,415 (38.1%) |
| 5 – Excellent | 378 (10.2%) |

# Supplementary Table 3. Multivariable models with AI-QCT characteristics for prediction of the 5-component secondary outcome of CV death, MI or hospitalization for unstable angina, heart failure or resuscitated cardiac arrest

|  | **MODEL 1**  **CLINICAL CHARACTERISTICS** | | **MODEL 2**  **CLINICAL + NUMBER OF DISEASED VESSELS** | | **MODEL 3**  **CLINICAL + NUMBER OF DISEASED VESSELS + ATHEROSCLEROSIS QUANTIFICATION** | |
| --- | --- | --- | --- | --- | --- | --- |
| **Multivariable model parameters** | **HR (95% CI)** | **p-value** | **HR (95% CI)** | **p-value** | **HR (95% CI)** | **p-value** |
| *Clinical characteristics* |  |  |  |  |  |  |
| Age | 1.36 (1.17, 1.57) | 0.001 | 1.37 (1.18, 1.59) | 0.001 | 1.23 (1.05, 1.44) | 0.010 |
| Female sex | 1.00 (0.78, 1.29) | 0.976 | 1.10 (0.85, 1.41) | 0.470 | 1.17 (0.90, 1.51) | 0.244 |
| Active smoking | 1.14 (0.92, 1.41) | 0.185 | 1.15 (0.93, 1.43) | 0.125 | 1.09 (0.88, 1.36) | 0.385 |
| Diabetes | 1.31 (1.07, 1.59) | 0.007 | 1.26 (1.04, 1.53) | 0.021 | 1.23 (1.01, 1.50) | 0.041 |
| Hypertension | 1.86 (1.43, 2.41) | 0.001 | 1.83 (1.41, 2.38) | 0.001 | 1.82 (1.40, 2.37) | 0.001 |
| Ejection fraction | 0.86 (0.77, 0.95) | 0.004 | 0.86 (0.78, 0.96) | 0.007 | 0.87 (0.78, 0.97) | 0.012 |
| Known CAD | 1.03 (0.81, 1.31) | 0.778 | 1.06 (0.83, 1.35) | 0.641 | 1.07 (0.84, 1.36) | 0.573 |
| eGFR | 0.94 (0.81, 1.08) | 0.362 | 0.94 (0.82, 1.08) | 0.404 | 0.92 (0.80, 1.06) | 0.259 |
| High intensity statin at randomization | 1.07 (0.87, 1.32) | 0.492 | 1.07 (0.87, 1.32) | 0.505 | 1.08 (0.88, 1.33) | 0.445 |
| **AI-QCT stenosis severity** |  |  |  |  |  |  |
| *AI-QCT number of diseased vessels* |  |  |  | 0.001 |  | 0.165 |
| Less than 50% stenosis |  |  | Reference |  | Reference |  |
| 1VD |  |  | 1.20 (0.90, 1.60) |  | 1.06 (0.78, 1.42) |  |
| 2VD |  |  | 1.57 (1.16, 2.11) |  | 1.17 (0.84, 1.63) |  |
| 3VD/LMD |  |  | 2.16 (1.57, 2.96) |  | 1.46 (1.00, 2.13) |  |
| **AI-QCT atherosclerosis parameters** |  |  |  |  |  |  |
| Total plaque volume |  |  |  |  | 1.49 (1.20, 1.85) | 0.001 |
| Low-density non-calcified plaque volume |  |  |  |  | 1.05 (0.91, 1.22) | 0.494 |
| Number of high-risk plaques |  |  |  |  | 1.15 (0.97, 1.36) | 0.116 |
| Proximal non-calcified plaque volume |  |  |  |  | 0.85 (0.69, 1.05) | 0.134 |
| Diffuseness |  |  |  |  | 1.05 (0.89, 1.23) | 0.567 |
| Average lumen area |  |  |  |  | 0.77 (0.66, 0.89) | 0.001 |
| Remodeling index ≥1.5 |  |  |  |  | 0.93 (0.75, 1.16) | 0.524 |

Shown are multivariable Cox regression models including clinical characteristics (Model 1), clinical and AI-QCT stenosis parameters (Model 2) and a model with clinical, AI-QCT stenosis and AI-QCT atherosclerotic variables (Model 3). A square root transformation was applied to plaque variables that exhibited skewed distributions (total plaque volume, low density non-calcified plaque volume, proximal non-calcified plaque volume). Continuously measured variables were scaled to represent an interquartile (IQR) increase from the 25^th^ to 75^th^ percentile. For total plaque volume, low-density non-calcified plaque volume and proximal non-calcified plaque volume, these increases were from 274 to 832 (Q1-Q3) mm^3^, from 2 to 16 (Q1-Q3) mm^3^ and from 118 to 312 (Q1-Q3) mm^3^, respectively. For age, the interquartile increase was from 57 to 70 (Q1-Q3) years, for eGFR, this was from 73 to 101 (Q1-Q3) ml/min/1.73m^2^. For total plaque volume, low-density non-calcified plaque volume and proximal non-calcified plaque volume, these increases were 559 mm^3^, 14 mm^3^ and 194 mm^3^, respectively. AI-QCT, Atherosclerosis Imaging-Quantitative Computed Tomography analysis.

# Supplementary Table 4. Performance of multivariable models with AI-QCT characteristics for the 5-component secondary outcome of CV death, MI or hospitalization for unstable angina, heart failure or resuscitated cardiac arrest

|  | | **MODEL 1** | **MODEL 2** | | **MODEL 3** | | |
| --- | --- | --- | --- | --- | --- | --- | --- |
|  | | **Clinical characteristics** | **Model 1 + number of diseased vessels** | **p-value vs model 1** | **Model 2 + atherosclerosis**  **quantification** | **p-value vs model 1** | **p-value vs model 2** |
| **6 MONTHS** | **AUC** | 0.634 (0.591-0.678) | 0.661 (0.619-0.704) | 0.018 | 0.676 (0.631-0.721) | 0.016 | 0.270 |
|  | **Brier score** | 0.0405 | 0.0403 | 0.060 | 0.0399 | 0.006 | 0.014 |
|  | **NRI** | Reference | vs M1: 0.31 (0.11-0.48) | | vs M1: 0.40 (0.23-0.57)  vs M2: 0.30 (0.11-0.47) | | |
| **2 YEARS** | **AUC** | 0.621 (0.588-0.654) | 0.639 (0.606-0.672) | 0.048 | 0.655 (0.621-0.689) | 0.008 | 0.091 |
|  | **Brier score** | 0.0768 | 0.0765 | 0.301 | 0.0754 | 0.004 | 0.002 |
|  | **NRI** | Reference | vs M1: 0.28 (0.11-0.40) | | vs M1: 0.31 (0.20-0.45)  vs M2: 0.24 (0.13-0.39) | | |
| **4 YEARS** | **AUC** | 0.613 (0.580-0.646) | 0.624 (0.590-0.658) | 0.255 | 0.651 (0.618-0.684) | 0.004 | 0.007 |
|  | **Brier score** | 0.1070 | 0.1067 | 0.579 | 0.1045 | 0.007 | 0.002 |
|  | **NRI** | Reference | vs M1: 0.22 (0.09-0.33) | | vs M1: 0.30 (0.21-0.45)  vs M2: 0.24 (0.15-0.40) | | |

AI-QCT, Atherosclerosis Imaging-Quantitative Computed Tomography analysis; AUC, area under the curve; CCTA, coronary CT angiography; CV death, cardiovascular death; MI, myocardial infarction; NRI, net reclassification improvement; M1, model 1; M2, model 2.

# Supplementary Table 5. Sensitivity analysis: Performance of multivariable models with AI-QCT characteristics for prediction of the primary outcome of CV death or MI restricted to the conservative treatment group (n=1,839)

|  | | **MODEL 1** | **MODEL 2** | | **MODEL 3** | | |
| --- | --- | --- | --- | --- | --- | --- | --- |
|  | | **Clinical characteristics** | **Model 1 + number of diseased vessels** | **p-value vs model 1** | **Model 2 + atherosclerosis**  **quantification** | **p-value vs model 1** | **p-value vs model 2** |
| **6 MONTHS** | **AUC (95% CI)** | 0.632 (0.557-0.708) | 0.666 (0.597-0.735) | 0.221 | 0.688 (0.614-0.762) | 0.075 | 0.251 |
|  | **Brier score** | 0.0274 | 0.0273 | 0.364 | 0.0272 | 0.311 | 0.544 |
|  | **NRI** | Reference | vs M1: 0.41 (0.06-0.67) | | vs M1: 0.32 (0.11-0.73)  vs M2: 0.22 (-0.06-0.59) | | |
| **2 YEARS** | **AUC (95% CI)** | 0.627 (0.577-0.677) | 0.666 (0.618-0.714) | 0.021 | 0.676 (0.627-0.726) | 0.011 | 0.359 |
|  | **Brier score** | 0.0684 | 0.0677 | 0.121 | 0.0669 | 0.033 | 0.076 |
|  | **NRI** | Reference | vs M1: 0.42 (0.20-0.59) | | vs M1: 0.34 (0.19-0.61)  vs M2: 0.23 (0.08-0.46) | | |
| **4 YEARS** | **AUC (95% CI)** | 0.622 (0.573-0.671) | 0.660 (0.611-0.709) | 0.018 | 0.685 (0.638-0.731) | 0.002 | 0.080 |
|  | **Brier score** | 0.1026 | 0.1012 | 0.125 | 0.0987 | 0.010 | 0.016 |
|  | **NRI** | Reference | vs M1: 0.35 (0.16-0.50) | | vs M1: 0.39 (0.23-0.59)  vs M2: 0.25 (0.13-0.51) | | |

For this population, the primary outcome of CV death or MI occurred in 194 patients. AI-QCT, Atherosclerosis Imaging-Quantitative Computed Tomography analysis; AUC, area under the curve; CCTA, coronary CT angiography; CV death, cardiovascular death; MI, myocardial infarction; NRI, net reclassification improvement; M1, model 1; M2, model 2.

# Supplementary Table 6. Sensitivity analysis: Performance of multivariable models with AI-QCT characteristics for prediction of the primary outcome of CV death or MI including patients with prior CABG (n=3,759)

|  | | **MODEL 1** | **MODEL 2** | | **MODEL 3** | | |
| --- | --- | --- | --- | --- | --- | --- | --- |
|  | | **Clinical characteristics** | **Model 1 + number of diseased vessels** | **p-value vs model 1** | **Model 2 + atherosclerosis**  **quantification** | **p-value vs model 1** | **p-value vs model 2** |
| **6 MONTHS** | **AUC (95% CI)** | 0.637 (0.593-0.681) | 0.668 (0.625-0.711) | 0.013 | 0.687 (0.641-0.733) | 0.005 | 0.170 |
|  | **Brier score** | 0.0362 | 0.0360 | 0.056 | 0.0357 | 0.005 | 0.017 |
|  | **NRI** | Reference | vs M1: 0.38 (0.16-0.56) | | vs M1: 0.40 (0.23-0.59)  vs M2: 0.21 (0.07-0.46) | | |
| **2 YEARS** | **AUC (95% CI)** | 0.617 (0.583-0.652) | 0.640 (0.606-0.674) | 0.025 | 0.657 (0.621-0.692) | 0.004 | 0.092 |
|  | **Brier score** | 0.0696 | 0.0692 | 0.202 | 0.0683 | 0.006 | 0.008 |
|  | **NRI** | Reference | vs M1: 0.33 (0.14-0.45) | | vs M1: 0.31 (0.20-0.48)  vs M2: 0.20 (0.08-0.37) | | |
| **4 YEARS** | **AUC (95% CI)** | 0.606 (0.572-0.640) | 0.616 (0.581-0.650) | 0.344 | 0.646 (0.612-0.680) | 0.003 | 0.003 |
|  | **Brier score** | 0.1000 | 0.1000 | 0.963 | 0.0981 | 0.030 | 0.002 |
|  | **NRI** | Reference | vs M1: 0.25 (0.10-0.36) | | vs M1: 0.31 (0.22-0.47)  vs M2: 0.24 (0.16-0.40) | | |

For this population, the primary outcome of CV death or MI occurred in 392 patients. AI-QCT, Atherosclerosis Imaging-Quantitative Computed Tomography analysis; AUC, area under the curve; CABG, coronary artery bypass grafting; CCTA, coronary CT angiography; NRI, net reclassification improvement; M1, model 1; M2, model 2.

# Supplementary Table 7. Sensitivity analysis: Performance of multivariable models with AI-QCT characteristics for prediction of spontaneous MI

|  | | **MODEL 1** | **MODEL 2** | | **MODEL 3** | | |
| --- | --- | --- | --- | --- | --- | --- | --- |
|  | | **Clinical characteristics** | **Model 1 + number of diseased vessels** | **p-value vs model 1** | **Model 2 + atherosclerosis**  **quantification** | **p-value vs model 1** | **p-value vs model 2** |
| **6 MONTHS** | **AUC (95% CI)** | 0.629 (0.567-0.692) | 0.664 (0.605-0.723) | 0.039 | 0.676 (0.613-0.739) | 0.033 | 0.519 |
|  | **Brier score** | 0.0190 | 0.0189 | 0.115 | 0.0189 | 0.156 | 0.017 |
|  | **NRI** | Reference | vs M1: 0.40 (0.09-0.63) | | vs M1: 0.38 (0.12-0.68)  vs M2: 0.18 (-0.02-0.51) | | |
| **2 YEARS** | **AUC (95% CI)** | 0.642 (0.599-0.685) | 0.655 (0.611-0.699) | 0.201 | 0.672 (0.626-0.718) | 0.028 | 0.117 |
|  | **Brier score** | 0.0397 | 0.0395 | 0.074 | 0.0392 | 0.013 | 0.057 |
|  | **NRI** | Reference | vs M1: 0.29 (0.09-0.47) | | vs M1: 0.34 (0.23-0.57)  vs M2: 0.26 (0.11-0.48) | | |
| **4 YEARS** | **AUC (95% CI)** | 0.646 (0.604-0.686) | 0.643 (0.599-0.687) | 0.799 | 0.669 (0.626-0.713) | 0.116 | 0.032 |
|  | **Brier score** | 0.0587 | 0.0586 | 0.723 | 0.0579 | 0.049 | 0.028 |
|  | **NRI** | Reference | vs M1: 0.16 (0.01-0.32) | | vs M1: 0.34 (0.20-0.51)  vs M2: 0.26 (0.13-0.47) | | |

Spontaneous MI occurred in 205 patients. AI-QCT, Atherosclerosis Imaging-Quantitative Computed Tomography analysis; AUC, area under the curve; CCTA, coronary CT angiography; NRI, net reclassification improvement; MI, myocardial infarction; M1, model 1; M2, model 2.

# Supplementary Table 8. Sensitivity analysis: Performance of multivariable models with visual stenosis assessment of number of diseased vessels.

|  | | **MODEL 1** | **MODEL 2** | | **MODEL 3** | | |
| --- | --- | --- | --- | --- | --- | --- | --- |
|  | | **Clinical characteristics** | **Model 1 + number of diseased vessels by visual assessment** | **p-value vs model 1** | **Model 2 + atherosclerosis**  **quantification** | **p-value vs model 1** | **p-value vs model 2** |
| **6 MONTHS** | **AUC (95% CI)** | 0.637 (0.592-0.682) | 0.703 (0.661-0.744) | <0.001 | 0.704 (0.659-0.750) | <0.001 | 0.888 |
|  | **Brier score** | 0.0360 | 0.0356 | 0.002 | 0.0353 | <0.001 | 0.022 |
|  | **NRI** | Reference | vs M1: 0.52 (0.30-0.68) | | vs M1: 0.50 (0.28-0.66)  vs M2: 0.25 (0.08-0.46) | | |
| **2 YEARS** | **AUC (95% CI)** | 0.617 (0.582-0.652) | 0.666 (0.633-0.699) | <0.001 | 0.671 (0.636-0.706) | <0.001 | 0.523 |
|  | **Brier score** | 0.0690 | 0.0681 | 0.003 | 0.0671 | <0.001 | 0.003 |
|  | **NRI** | Reference | vs M1: 0.40 (0.23-0.52) | | vs M1: 0.41 (0.25-0.55)  vs M2: 0.19 (0.10-0.38) | | |
| **4 YEARS** | **AUC (95% CI)** | 0.608 (0.574-0.643) | 0.648 (0.615-0.682) | 0.002 | 0.667 (0.634-0.701) | <0.001 | 0.043 |
|  | **Brier score** | 0.0985 | 0.0974 | 0.070 | 0.0957 | 0.001 | 0.008 |
|  | **NRI** | Reference | vs M1: 0.32 (0.17-0.44) | | vs M1: 0.42 (0.27-0.53)  vs M2: 0.21 (0.13-0.38) | | |

Patients (n=876) with unevaluable vessels according to the visual stenosis assessment were considered as a separate category (stenosis categories: none, 1VD, 2VD, 3VD and non-evaluable). AI-QCT, Atherosclerosis Imaging-Quantitative Computed Tomography analysis; AUC, area under the curve; CCTA, coronary CT angiography; NRI, net reclassification improvement; MI, myocardial infarction; M1, model 1; M2, model 2.

# ISCHEMIA Committee, CCC, Trial-Related Personnel

| **Past and Current Committee Members** |
| --- |
| **Leadership Committee** |
| Judith S. Hochman (Chair) |
| David J. Maron (Co-Chair) |
| William Boden (Co-Principal Investigator) |
| Robert Harrington (Co-Principal Investigator) |
| Gregg W. Stone (Co-Principal Investigator) |
| David Williams (Co-Principal Investigator) |
|  |
| **Executive Committee** |
| Judith S. Hochman (Chair) |
| David J. Maron (Co-Chair) |
| Karen P. Alexander |
| Sripal Bangalore |
| Jeffrey Berger |
| William Boden |
| Robert Harrington |
| Daniel Mark |
| Sean M. O'Brien |
| Harmony R. Reynolds |
| Yves Rosenberg |
| Leslee J. Shaw |
| John Spertus |
| Gregg W. Stone |
|  |
| **Steering Committee** |
| Judith S. Hochman (Chair) |
| David J. Maron (Co-Chair) |
| *Members of Executive Committee* |
| Christie Ballantyne*** |
| Daniel Berman |
| Rafael Beyar*** |
| Balram Bhargava |
| Chris Buller*** |
| Antonio (Tony) Carvalho** |
| Bernard R. Chaitman |
| Rafael Diaz*** |
| Rolf Doerr |
| Vladimir Dzavik |
| Shaun Goodman |
| Gilbert Gosselin |
| Rory Hachamovitch*** |
| Christian Hamm*** |
| Claes Held |
| Malte Helm*** |
| Kurt Huber*** |
| Lixin Jiang |
| Matyas Keltai |
| Shun Kohsaka |
| Irene Lang*** |
| Renato Lopes |
| Jose Lopez-Sendon |
| Aldo Maggioni |
| John Mancini |
| C. Noel Bairey Merz |
| James Min |
| Eric Peterson*** |
| Michael H. Picard |
| Witold Ruzyllo |
| Joseph Selvanayagam |
| Roxy Senior |
| Tali Sharir |
| Gabriel Steg |
| Hanna Szwed |
| Frans Van de Werf*** |
| William Weintraub |
| Harvey White |
| David Williams |
|  |
| **Optimal Medical Therapy Committee** |
| William Boden (Co-Chair) |
| David J. Maron (Co-Chair) |
| Christie Ballantyne |
| Sripal Bangalore |
| Karen Calfas **** |
| Bernard R. Chaitman |
| Mary Ann Champagne |
| Michael Davidson |
| Jerome Fleg |
| Peter A. McCullough |
| Jonathan Newman |
| Peter Stone |
|  |
| **Optimal Revascularization Therapy Planning Committee** |
| Gregg W. Stone (Chair) |
| **Subcommittee: CABG** |
| Philippe Menasche (Co-Chair) |
| Sripal Bangalore |
| Michael Davidson**** |
| Stephen Fremes |
| Robert Guyton |
| Michael Mack |
| Fred Mohr |
| Anupama Rao |
| Joe Sabik |
| Oz Shapira |
| David Taggart |
| James Tatoulis |
|  |
| **Subcommittee: PCI** |
| David Williams (Co-Chair) |
| Sripal Bangalore |
| Jim Blankenship |
| Sorin Brener |
| Chris Buller |
| Antonio Colombo |
| Bernard de Bruyne |
| Philippe Généreux |
| Robert Harrington |
| Dean Kereiakes |
| Thierry Lefevre |
| Jeffrey Moses |
|  |
| **Clinical Events** |
| **Endpoint Definition Panel** |
| Bernard R. Chaitman (Chair) |
| Karen P. Alexander |
| Judith S. Hochman |
| Ken Mahaffey |
| David J. Maron |
| Gregg W. Stone |
| Harvey White |
| **Clinical Event Review Committee** |
| Bernard R. Chaitman (Chair) |
| Salvador Cruz-Flores |
| Nicholas Danchin |
| Eli Feen |
| Mario J. Garcia |
| Paul Hauptman |
| Abhay A. Laddu |
| Eugene Passamani |
| Ileana L. Pina |
| Maarten Simoons |
| Hicham Skali |
| Kristian Thygesen |
| David Waters |
| **CEC Administrative Group** |
| Karen P. Alexander |
| Patricia Endsley*** |
| Gerard Esposito |
| Jeffrey Kanters |
| John Pownall |
| Dimitrios Stournaras |
|  |
| **ISCHEMIA Imaging Committee** |
| Leslee J. Shaw (Chair) |
| Daniel Berman |
| Matthias Friedrich |
| Rory Hachamovitch |
| Raymond Kwong |
| John Mancini |
| James Min |
| Dana Oliver |
| Michael H. Picard |
| Harmony R. Reynolds |
|  |
| **Biostatistics Planning Committee** |
| Frank Harrell (Chair) |
| Jeffrey Blume |
| Kerry Lee |
| Sean M. O'Brien |
|  |
| **BioRepository Committee** |
| Jeffrey Berger (Chair) |
| Claes Held |
| Iftikhar Kullo |
| Bruce McManus |
| Kristin Newby |
|  |
| **EQOL Committee** |
| Daniel Mark (Co-Chair) |
| John Spertus (Co-Chair) |
| David Cohen |
| William Weintraub |
|  |
| **Recruitment for Women & Minorities** |
| C. Noel Bairey Merz (Chair) |
| Raffaele Bugiardini |
| Jelena Celutkiene |
| Jorge Escobedo |
| Angela Hoye |
| Radmila Lyubarova |
| Deirdre Mattina |
| Jesus Peteiro |
| Harmony R. Reynolds |
| Paola Smanio |
|  |
| **Publications** |
| David J. Maron (Chair) |
| Karen P. Alexander |
| Sripal Bangalore |
| Jeffrey Berger |
| William Boden |
| Robert Harrington |
| Judith S. Hochman |
| Sean M. O'Brien |
| Harmony R. Reynolds |
| Yves Rosenberg |
| Gregg W. Stone |
| **Publication Subcommittees** |
| **Economics** |
| Daniel Mark (Chair) |
| John Spertus |
| **QOL** |
| John Spertus (Chair) |
| Daniel Mark |
| **Stress Testing** |
| Leslee J. Shaw (Chair) |
| Dan Berman |
| Bernard R. Chaitman |
| Jerome Fleg |
| Raymond Kwong |
| Michael H. Picard |
| Harmony R. Reynolds |
| Roxy Senior |
| **CCTA** |
| James Min (Chair) |
| Jonathan Leipsic |
| John Mancini |
| **Angiography/Optimal Revascularization Therapy** |
| Gregg W. Stone (Chair) |
| Ziad Ali (Co-chair) |
| Sripal Bangalore |
| David Williams |
| (Philippe Genereux, former Chair, Angiography Subcommittee)* |
| **Optimal Medical Therapy** |
| William Boden (Co-Chair) |
| David J. Maron (Co-Chair) |
| Jerome Fleg |
| Jonathan Newman |
| **Biorepository** |
| Jeffrey Berger (Chair) |
| **CEC** |
| Bernard R. Chaitman (Chair) |
| Karen P. Alexander |
| **CKD** |
| Sripal Bangalore (Chair) |
| Karen P. Alexander |
| Jerome Fleg |
| Judith S. Hochman |
| David J. Maron |
| Roy Mathew |
| Sean M. O'Brien |
| Harmony R. Reynolds |
| Mandeep Sidhu |
| **CIAO** |
| Harmony R. Reynolds (Chair) |
|  |
|  |
| **DSMB Members** |
| Lawrence Friedman (Chair) |
| Jeffrey Anderson |
| Jessica Berg *** |
| David DeMets |
| C. Michael Gibson |
| Gervasio Lamas |
| Nicole Deming |
| Jonathan Himmelfarb |
| Pamela Ouyang |
| Pamela Woodard |
|  |
| **Independent Statistical Analysis Center for DSMB Reporting** |
| Frank Harrell |
| Samuel Nwosu |
|  |
| **NHLBI Program Staff** |
| **Project Office** |
| Yves Rosenberg (Project Officer) |
| Jerome Fleg |
| Ruth Kirby |
| **Statisticians** |
| Neal Jeffries |
|  |
| **ISCHEMIA Clinical Coordinating Center (CCC)** |
| **Study Leadership** |
| Judith S. Hochman (Study Chair, Director of CCC) |
| David J. Maron (Study Co-Chair, Co-Director of CCC, US Country Leader) |
| **CCC Faculty** |
| Sripal Bangalore (Optimal Revascularization Therapy CCC Director, Regional Leader) |
| Jeffrey Berger (Director of the Biorepository, Regional Leader) |
| William Boden (US-VA Regional Leader) |
| Jonathan Newman (Optimal Medical Therapy CCC Director, Regional Leader) |
| Harmony R. Reynolds (Associate Director of CCC, CCC Imaging Lead, Regional Leader) |
| Mandeep Sidhu (US-VA Regional Co-Leader) |
| ***Program Directors*** |
| Jean E. Denaro**** |
| Stephanie Mavromichalis |
| ***Project Managers*** |
| Kevin Chan |
| Gia Cobb* |
| Aira Contreras |
| Diana Cukali* |
| Stephanie Ferket*** |
| Andre Gabriel*** |
| Antonietta Hansen* |
| Arline Roberts |
| ***Clinical Research Associates*** |
| Michelle Chang |
| Sharder Islam* |
| Graceanne Wayser* |
| Solomon Yakubov*** |
| Michelle Yee |
| ***Clinical Trial Assistants*** |
| Caroline Callison |
| Isabelle Hogan |
| Albertina Qelaj* |
| Charlotte Pirro* |
| Kerrie Van Loo |
| Brianna Wisniewski* |
| ***Grants and Finance Administration*** |
| Margaret Gilsenan (Grants Manager) |
| Bevin Lang |
| Samaa Mohamed |
| ***Publications Team*** |
| Shari Esquenazi-Karonika (Publications Manager) |
| Patenne D. Mathews |
| Anna Naumova |
| Jihyun Lyo* |
| ***Data Analyst*** |
| Vincent Setang* |
| Mark Xavier* |
|  |
| **Statistical and Data Coordinating Center (SDCC)** |
| Sean M. O’Brien (Principal Investigator) |
| Karen P. Alexander (Co-Principal Investigator) |
|  |
| **Economics and Quality of Life Coordinating Center (EQOL CC)** |
| *Duke Clinical Research Institute, Durham, NC* |
| Daniel B. Mark (Principal Investigator) |
| Kevin Anstrom |
| Khaula Baloch |
| Janet Blount |
| Patricia Cowper |
| Linda Davidson-Ray |
| Laura Drew |
| Tina Harding |
| J David Knight |
| Diane Minshall Liu |
| Betsy O’Neal |
| Thomas Redick |
|  |
| *Saint Luke’s Mid America Heart Institute, Kansas City, MO* |
| John Spertus (Principal Investigator) |
| Philip Jones |
| Karen Nugent |
| Grace Jingyan Wang |
|  |
| **ISCHEMIA Imaging Coordinating Center (ICC)** |
| Leslee J. Shaw (Principal Investigator) |
| Lawrence Phillips |
| Abhinav Goyal |
| Holly Hetrick |
| Dana Oliver |
| *Nuclear Core Lab* |
| Daniel Berman (Director) |
| Sean W. Hayes (Co-Director) |
| John D. Friedman |
| R. James Gerlach |
| Mark Hyun |
| Romalisa Miranda-Peats |
| Piotr Slomka |
| Louise Thomson |
| *CMR Core Lab* |
| Raymond Y. Kwong (Director) |
| Matthias Friedrich (Director)*** |
| Francois Pierre Mongeon (Co-Director) |
| Steven Michael |
| *Echo Core Lab* |
| Michael H. Picard (Director) |
| Judy Hung |
| Marielle Scherrer-Crosbie |
| Xin Zeng |
|  |
| **ECG/ETT CoreLab** |
| Bernard R. Chaitman (Director) |
| Jane Eckstein |
| Bandula Guruge |
| Mary Streif |
|  |
| **Angiographic Core Lab** |
| Ziad Ali (Director) |
| Philippe Genereux (Director)*** |
| Maria A. Alfonso |
| Maria P. Corral |
| Javier J. Garcia |
| Jennifer Horst |
| Ivana Jankovic |
| Maayan Konigstein |
| Mitchel B. Lustre* |
| Yolayfi Peralta |
| Raquel Sanchez |
|  |
| **CCTA Core Lab** |
| James Min (Director) |
| Reza Arsanjani |
| Matthew Budoff |
| Kimberly Elmore |
| Millie Gomez |
| Cameron Hague |
| Niree Hindoyan |
| Jonathan Leipsic |
| GB John Mancini |
| Rine Nakanishi |
| M. Barbara Srichai-Parsia |
| Eunice Yeoh |
| Tricia Youn |
|  |
| **Academic Research Organizations (AROs)** |
| *Associazione Nazionale Medici Cardiologi Ospedalieri (ANMCO) -Italy & Switzerland* |
| Aldo P. Maggioni (Country Leader) |
| Francesca Bianchini |
| Martina Ceseri |
| Andrea Lorimer |
| Marco Magnoni |
| Francesco Orso |
| Laura Sarti |
| Martinia Tricoli* |
| *Brazilian Clinical Research Institute (BCRI) - Brazil* |
| Antonio Carvalho (Country Leader)** |
| Renato Lopes (Country Leader) |
| Lilian Mazza Barbosa |
| Tauane Bello Duarte |
| Tamara Colaiácovo Soares |
| Julia de Aveiro Morata |
| Pedro Carvalho |
| Natalia de Carvalho Maffei |
| Flávia Egydio* |
| Anelise Kawakami* |
| Janaina Oliveira* |
| Elissa Restelli Piloto* |
| Jaqueline Pozzibon*** |
| *Canadian Heart Research Centre (CHRC) - Canada* |
| Shaun Goodman (Country Leader) |
| Diane Camara |
| Neamat Mowafy |
| Caroline Spindler |
| *China Oxford Centre for International Health Research - China* |
| Lixin Jiang (Country Leader) |
| Hao Dai |
| Fang Feng |
| Jia Li |
| Li Li* |
| Jiamin Liu |
| Qiulan Xie |
| Haibo Zhang |
| Jianxin Zhang |
| Lihua Zhang |
| Liping Zhang |
| Ning Zhang |
| Hui Zhong |
| *Estudios Clínicos Latino America (ECLA) - Argentina* |
| Rafael Diaz*** |
| Claudia Escobar |
| Maria Eugenia Martin* |
| Andrea Pascual* |
| *Foundation for Biomedical Research of La Paz University Hospital (FIBHULP) - Spain* |
| José Lopez-Sendon (Country Leader) |
| Paloma Moraga |
| Victoria Hernandez |
| Almudena Castro |
| Maria Posada* |
| Sara Fernandez |
| José Luis Narro Villanueva |
| Rafael Selgas |
| *French Alliance for Cardiovascular Trials (FACT) - France* |
| Gabriel Steg (Country Leader) |
| Helene Abergel |
| Jean Michel Juliard |
| *Green Lane Coordinating Centre Ltd. (GLCC) -Malaysia, New Zealand, Singapore, Taiwan, Thailand* |
| Harvey White (Country Leader) |
| Caroline Alsweiler |
| *KU Leuven Research & Development - Belgium** |
| Frans Van de Werf (Country Leader) |
| Kathleen Claes |
| Kaatje Goetschalckx |
| Ann Luyten |
| Valerie Robesyn |
| *South Australian Health and Medical Research Institute Ltd (SAHMRI) - Australia* |
| Joseph B. Selvanayagam (Country Leader) |
| Deirdre Murphy |
|  |
| **Contract Research Organizations (CROs) for ISCHEMIA Trial** |
| *FOCUS Clinical Research Center d.o.o. Belgrade - Serbia* |
| Nevena Garcevic |
| Jelena Stojkovic |
| *iProcess Global Research Inc. - India* |
| Asker Ahmed |
| Richa Bhatt |
| Nitika Chadha* |
| Vijay Kumar* |
| Sadath Lubna*** |
| Pushpa Naik |
| Shruti Pandey* |
| Karthik Ramasamy* |
| Mohammed Saleem |
| Pratiksha Sharma |
| Hemalata Siddaram* |
|  |
| **past members / past organizations* |
| ***deceased* |

# ISCHEMIA Site Investigators and Coordinators

| Country (No.Randomizations) | Investigator(s) | Study Coordinator(s) | City & State | Institution (No. Randomizations) |
| --- | --- | --- | --- | --- |
|  |  |  | (if applicable) |  |
| *United States (853) |  |  |  |  |
| Country Leader |  |  |  |  |
| David J. Maron, MD |  |  |  |  |
| Regional Leader for VA Sites | |  |  |  |
| William E. Boden, MD |  |  |  |  |
|  | Kreton Mavromatis, MD | John Doan, MD | Decatur, GA | Atlanta VA Medical Center (139) |
|  | Jason Linefsky, MD | Raven Lee, CCRP |  |  |
|  |  | Risha Patel |  |  |
|  | Todd Miller, MD | So Yang Cho | Rochester, MN | Mayo Clinic (50) |
|  |  | Susan Milbrandt |  |  |
|  |  | Dawn Shelstad |  |  |
|  | Subhash Banerjee, MD | Preeti Kamath, BDS, MHA, CCRP | Dallas, TX | V.A. North Texas Health Care System (35) |
|  |  | Ishita Tejani, BDS, MS, MSPH |  |  |
|  | Harmony R. Reynolds, MD | Stanley E. Cobos, BA | New York, NY | NYU Langone Medical Center-Bellevue Hospital (26) |
|  | Jonathan D. Newman, MD, MPH | Kirsten J. Quiles, MS |  |  |
|  | Sripal Bangalore, MD | Raven R. Dwyer, MPH |  |  |
|  | Robert  M. Donnino, MD | Dalisa Espinosa, MBS |  |  |
|  | Lawrence M. Phillips, MD |  |  |  |
|  | Muhamed Saric, MD, PhD |  |  |  |
|  | Khaled Abdul-Nour, MD | Allison Schley, BS | Detroit, MI | Henry Ford Health System (21) |
|  |  | Heather Golden |  |  |
|  | Peter H. Stone, MD | Hermine Osseni, MS | Boston, MA | Brigham & Women's Hospital, Harvard Medical School (21) |
|  |  | Charlene Wiyarand |  |  |
|  |  | Peter Douglass, BA |  |  |
|  |  | Hayley Pomeroy, BA |  |  |
|  |  | Alexandra Craft, BA |  |  |
|  |  | Bethany Harvey, BA |  |  |
|  | James J. Jang, MD | Olivia Anaya | San Jose, CA | Kaiser Permanente San Jose (18) |
|  | Gennie Yee, MD | Phoebe Goold, RN |  |  |
|  | Steven Weitz, MD | Steven Giovannone | Schenectady, NY | Cardiology Associates of Schenectady P.C. (17) |
|  |  | Lori Pritchard, RN |  |  |
|  | Suzanne Arnold, MD | Rosann Gans, RN | Kansas City, MO | Saint Luke's Hospital (17) |
|  | James Henry O’Keefe, Jr, MD (PI from 2012-2016) | Paul Kennedy, RN |  |  |
|  | Michael D. Shapiro, DO | Shobana Ganesan, PhD | Portland, OR | Oregon Health & Science University (17) |
|  |  | David Schlichting, LPN |  |  |
|  |  | Aynun Naher |  |  |
|  | Mohammad El-Hajjar, MD |  | Albany, NY | Albany Medical Center Hospital (16) |
|  | Mandeep S. Sidhu, MD, MBA |  |  |  |
|  | Steven A. Fein, MD | Wendy L. Stewart, MS |  |  |
|  | Mikhail T. Torosoff, MD, PhD | Kristin M. Salmi, BS |  |  |
|  | Radmila Lyubarova, MD |  |  |  |
|  | Sulagna Mookherjee, MD |  |  |  |
|  | Krzysztof Drzymalski, MD |  |  |  |
|  | Edward O. McFalls, MD, PhD |  | Minneapolis, MN | Minneapolis VAMC (15) |
|  | Santiago A. Garcia, MD |  |  |  |
|  | Stefan C. Bertog, MD | Debra K. Johnson, RN |  |  |
|  | Rizwan A. Siddiqui, MD | Rebekah R. Herrmann, RN |  |  |
|  | Areef Ishani, MD |  |  |  |
|  | Ronnell A. Hansen, MD |  |  |  |
|  | Michel Georges Khouri, MD | Kristine Arges | Durham, NC | Duke University Medical Center (15) |
|  |  | Melissa LeFevre |  |  |
|  |  | Jennifer Tomfohr |  |  |
|  | Jonathan L. Goldberg, MS, MD | Kimberly Ann Byrne | Cleveland, OH | Louis Stokes Cleveland Veterans Affairs Medical Center (14) |
|  |  | Taissa Zappernick |  |  |
|  | Richard Goldweit, MD | Sallie Canada | Englewood, NJ | Englewood Hospital and Medical Center (13) |
|  |  | Meghana Kakade |  |  |
|  |  | Patricia Mieses |  |  |
|  |  | Stanley E. Cobos, BA | Brooklyn, NY | NYU-HHC Woodhull Hospital (12) |
|  |  | Raven R. Dwyer, MPH |  |  |
|  | Ronny A. Cohen, MD | Dalisa Espinosa, MBS |  |  |
|  | Brooks Mirrer, MD | Kirsten J. Quiles, MS |  |  |
|  | Victor Navarro, MD | Magdalena Rantinella, BS |  |  |
|  |  | Jessica Rodriguez, BS |  |  |
|  |  | Olivia Mancilla, BS |  |  |
|  | David E. Winchester, MD, MS | Susan Stinson, RN | Gainesville, FL | Malcom Randall VAMC (11) |
|  | Marvin Kronenberg, MD | Terry Weyand | Nashville, TN | Vanderbilt University Medical Center (11) |
|  | Philip Rogal, MD | Sherron C. Crook |  |  |
|  | Christopher McFarren, MD |  |  |  |
|  | John F. Heitner, MD | Jean Ho | Brooklyn, NY | New York -Presbyterian/Brooklyn Methodist Hospital (10) |
|  |  | Saadat Khan |  |  |
|  |  | Mahmoud Mohamed |  |  |
|  | Ira M. Dauber, MD | Mary R. Soltau, RN | Littleton, CO | South Denver Cardiology Associates, P.C. (10) |
|  |  | Delsa K. Rose, RN |  |  |
|  |  | Rebecca J. Wimmer, RN |  |  |
|  |  | Kathy E. Siegel, RN |  |  |
|  |  | Susan Derbyshire |  |  |
|  | Charles Cannan, MD | Michelle Dixon | Portland, OR | Providence Heart and Vascular Institute (10) |
|  |  | Gerald Leonard |  |  |
|  | Sriram Sudarshan, MD | Ciarra Heard, LVN | Wichita Falls, TX | Wichita Falls Heart Clinic (9) |
|  |  | Viviana Gabriel, LVN |  |  |
|  |  | Sukie Desire |  |  |
|  | Puja K. Mehta, MD |  | Atlanta, GA | Emory University (9) |
|  | Michael McDaniel, MD | Fauzia Rashid, PhD |  |  |
|  | Stamatios Lerakis, MD | Senait Asier |  |  |
|  | Arshed Quyyumi, MD | Keyur Patel |  |  |
|  | Nanette K. Wenger, MD |  |  |  |
|  | Chester M. Hedgepeth, MD, PhD | Jennifer Gillis, APRN | Warwick, RI | Kent Hospital (9) |
|  | Heather Hurlburt, MD | Megan Manocchia, RN |  |  |
|  | Alan Rosen, MD | Susan Moore, RN |  |  |
|  |  | Elizabeth Congdon |  |  |
|  | Zakir Sahul, MD | Gail Brandt | Ypsilanti, MI | Michigan Heart, PC (9) |
|  |  | Nora Marchelletta |  |  |
|  |  | Kristina Wippler |  |  |
|  | David Booth, MD | Yvonne Taul, RN | Lexington, KY | University of Kentucky (8) |
|  | Steve Leung, MD | Jennifer Isaacs, MS |  |  |
|  | Ahmed Abdel-Latif, MD, PhD | Viktoria Bulkley, RN |  |  |
|  | Hassan Reda, MD | Caroline Rodgers |  |  |
|  | Khaled Ziada, MD |  |  |  |
|  | Sampoornima Setty, MD | Kimberly E. Halverson, RHIT | La Crosse, WI | Gundersen Lutheran Medical Center (8) |
|  |  | Christine Roraff, RN |  |  |
|  |  | Jonean Thorsen, RN |  |  |
|  | Rajat S. Barua, MD, PhD | Amarachi Ojajuni | Kansas City, MO | Kansas City VA Medical Center (8) |
|  |  | Oni Olurinde |  |  |
|  |  | Kamalakar Surineni |  |  |
|  | Fadi Hage, MD | Badhma Valaiyapathi, MD | Birmingham, AL | UAB Vascular Biology and Hypertension Program (8) |
|  | Christiano Caldeira, MD |  |  |  |
|  | James E. Davies, MD |  |  |  |
|  | Massoud Leesar, MD |  |  |  |
|  | Jaekyeong Heo, MD |  |  |  |
|  | Amy Iskandrian, MD |  |  |  |
|  | Firas Al Solaiman, MD |  |  |  |
|  | Satinder Singh, MD |  |  |  |
|  | Khaled Dajani, MD | Carol M. Kartje, BSN | Maywood, IL | Loyola University Medical Center (8) |
|  | Mohammad El-Hajjar, MD |  | Albany, NY | Samuel Stratton VA Medical Center of Albany NY (7) |
|  | Paul Der Mesropian, MD |  |  |  |
|  | Joseph Sacco, MD | Michele Rawlins, NP |  |  |
|  | Brian McCandless, MD | Jennifer Thomson, MA |  |  |
|  | Marisa Orgera, MD |  |  |  |
|  | Mandeep S. Sidhu, MD, MBA (2012-2016 ) |  |  |  |
|  |  | Mary Colleen Rogge, RN | Cincinnati, OH | Cincinnati VA Medical Center (7) |
|  | Imran Arif, MD | Julie Bunke , BA |  |  |
|  | Hanan Kerr, MD | Kendra Unterbrink , PA |  |  |
|  |  | Jacqueline Fannon, RN |  |  |
|  |  | Cynthia Burman, NP |  |  |
|  | Jorge F. Trejo (Gutierrez), MD | Marcia F. Dubin, CCRP | Jacksonville, FL | Mayo Clinic Florida (7) |
|  | Gerald Fletcher, MD |  |  |  |
|  | Gary E. Lane, MD |  |  |  |
|  | Lynn M. Neeson, DNP |  |  |  |
|  | Pragnesh P. Parikh, MD |  |  |  |
|  | Peter M. Pollak, MD |  |  |  |
|  | Brian P. Shapiro, MD |  |  |  |
|  | Kevin Landolfo, MD |  |  |  |
|  | Anthony Gemignani, MD | Sarah Beaudry, RN | White River Junction, VT | VAMC-White River Junction (7) |
|  | Daniel O'Rourke, MD |  |  |  |
|  | Judith L. Meadows, MD | Stephanie A. Tirado, RN | West Haven, CT | VA Connecticut Healthcare System (7) |
|  |  | Janet Halliday |  |  |
|  |  | Pamela Julian |  |  |
|  | Jason T. Call, MD | Stephanie, M. Lane, RN, BSN, CCRN | Winchester, VA | Winchester Cardiology and Vascular Medicine, PC (7) |
|  |  | Jennifer L. Stanford, RN, MSN |  |  |
|  | Joseph Hannan, MD |  | Worcester, MA | Saint Vincent Hospital at Worcester Medical Center (7) |
|  | Robert Bojar, MD | Patricia Arsenault, RN |  |  |
|  | Deepti Kumar, MD | Pamela Sigel, RN |  |  |
|  | John Mukai, MD |  |  |  |
|  | Edward T. Martin, MS, MD | Miriam Brooks | Tulsa, OK | Oklahoma Heart Institute (7) |
|  | Gabriel Vorobiof, MD | Ladda Douangvila | Los Angeles, CA | Ronald Reagan UCLA Medical Center (7) |
|  |  | Rubine Gevorgyan |  |  |
|  | Alec Moorman, MD | Fatima Ranjbaran, RN | Seattle, WA | University of Washington Medical Center (7) |
|  |  | Bryn Smith, BS |  |  |
|  |  | Carly Ohmart |  |  |
|  | Scott Kinlay, MBBS, PhD |  | West Roxbury, MA | VA Boston Healthcare System (6) |
|  | Robert J. Hamburger, MD |  |  |  |
|  | Thomas P. Rocco, MD | Samantha Ly, MA |  |  |
|  | Deepak L. Bhatt, MD, MPH | Margot C. Quinn, BA |  |  |
|  | Kevin Croce, MD, PhD | Sara Temiyasathit, PhD |  |  |
|  | Jacquelyn A Quin, MD | Jacquelyn Do, MPH |  |  |
|  | Jati Anumpa, MD | Desiree Tobin, MPH |  |  |
|  | Marco Zenati, MD, MSc |  |  |  |
|  | David P Faxon, MD |  |  |  |
|  | Glenn Rayos, MD | Jennifer Langdon | Daytona Beach, FL | Daytona Heart Group (6) |
|  |  | Marcia Werner Bayer |  |  |
|  | Ashraf Seedhom, MD | Amanda O'Malley | Albany, NY | Capital Cardiology Associates (6) |
|  | Lance Sullenberger, MD | Erin Orvis |  |  |
|  | Gregory Kumkumian, MD | Mandy Murphy, RN | Bethesda, MD | NIH Heart Center at Suburban Hospital (6) |
|  |  | Ann Greenberg, RN |  |  |
|  |  | Margaret Iraola, RN |  |  |
|  | Steven P. Sedlis, MD | Leandro C.Maranan, CCRC | New York, NY | VA New York Harbor Health Care System (6) |
|  | Robert M. Donnino, MD |  |  |  |
|  | Jeffrey Lorin, MD |  |  |  |
|  | Jacqueline E. Tamis-Holland, MD | Ammy Malinay, RN | Ridgewood, NJ | Mount Sinai Saint Luke's Hospital (6) |
|  | Robert Kornberg, MD |  |  |  |
|  | Robert Leber, MD |  |  |  |
|  | Souheil Saba, MD | Candice P. Edillo, RN | Southfield, MI | Providence - Providence Park Hospital (6) |
|  | Michael W. Lee, MD |  |  |  |
|  | Delano R. Small, MD |  |  |  |
|  | Wassim Nona, MD |  |  |  |
|  | Patrick B. Alexander, MD |  |  |  |
|  | Iram Rehman, MD |  |  |  |
|  | Umesh Badami, MD | Ann Ostrander, RN | Saginaw, MI | Covenant Medical Center, Inc. (5) |
|  |  | Stephanie Wasmiller, RN |  |  |
|  | Kevin Marzo, MD | Wendy Drewes, RN | Mineola, NY | NYU Winthrop (5) |
|  |  | Dipti Patel, RN |  |  |
|  | Inga H. Robbins, MD |  | Pomona, NJ | AtlantiCare Regional Medical Center (5) |
|  | Howard A. Levite, MD | Jackie M White, RN, BSN CCRC |  |  |
|  | Sanjay Shetty, MD | Alison Hallam |  |  |
|  | Mayuri Patel, MD |  |  |  |
|  | Glenn S. Hamroff, MD | Benjamin J Spooner, RPA-C | Cortlandt Manor, NY | NYP Medical Medical Group Hudson Valley Cardiology (5) |
|  |  | Linda M Hollenweger, LPN,CCRC |  |  |
|  | Raymond W. Little, MD | Holly Little | Houston, TX | Houston Heart & Vascular Associates (5) |
|  | Brandi D. Zimbelman, FNP-C | Tiffany Little |  |  |
|  | Charles Y. Lui, MD | Nona A Eskelson, RN | Salt Lake City, UT | Salt Lake City VA Medical Center (4) |
|  | Brigham R. Smith, MD |  |  |  |
|  | Daniel P. Vezina, MD, MSC |  |  |  |
|  | Lillian L. Khor, MBBCh, MSc |  |  |  |
|  | Josephine D. Abraham, MD, MPH |  |  |  |
|  | David A. Bull, MD |  |  |  |
|  | Stephen H. McKellar, MD, MSc |  |  |  |
|  | David Booth, MD | Yvonne Taul, RN | Lexington, KY | Lexington VA Medical Center (4) |
|  | John Kotter, MD | Caroline Rodgers, RN |  |  |
|  | Ahmed Abdel-Latif, MD, PhD | Jennifer Isaacs, MS |  |  |
|  |  | Viktoria Bulkley |  |  |
|  | Bob Hu, MD | Renee Kaneshiro | Palo Alto, CA | Palo Alto Medical Foundation Research Institute (4) |
|  | Arthur J. Labovitz, MD |  | Tampa, FL | University of South Florida (4) |
|  | Michael Berlowitz, MD | Bonnie J. Kirby, RN, MSN |  |  |
|  | Philip Rogal, MD | Nhi N. Tran, MS |  |  |
|  | Christopher McFarren, MD | Catherine Jahrsdorfer, RN, BSN |  |  |
|  | Fadi Matar, MD |  |  |  |
|  | Christiano Caldeira, MD |  |  |  |
|  | David J. Maron, MD |  | Stanford, CA | Stanford University School of Medicine (4) |
|  | Fatima Rodriguez, MD, MPH | Reem Yunis, PhD |  |  |
|  | Ingela Schnittger, MD | Jhina Patro |  |  |
|  | William F. Fearon, MD |  |  |  |
|  | Prakash Deedwania, MD | Antonia Vega | Fresno, CA | UCSF - Fresno Community Regional Medical Center (4) |
|  | Kiran Reddy, MD |  |  |  |
|  | Joseph Sweeny, MD | Hugo Bloise-Adames | New York, NY | Icahn School of Medicine at Mount Sinai (4) |
|  |  | Santa Jimenez |  |  |
|  |  | Nicole Saint Vrestil |  |  |
|  |  | Reyna Bhandari |  |  |
|  | Christopher Spizzieri, MD | Danielle Schade | Camp Hill, PA | Holy Spirit Hospital Cardiovascular Institute (4) |
|  |  | Roxanne Yost |  |  |
|  | Claudia P Hochberg, MD | Paula Beardsley | Boston, MA | Boston Medical Center (4) |
|  |  | Denise Fine |  |  |
|  | William D. Salerno, MD | Jana Tancredi, RN, MA/MSN, CCRN | Saddle Brook, NJ | Hackensack University Medical Center (4) |
|  |  | Patricia Arakelian |  |  |
|  |  | Susan Mathus |  |  |
|  |  | Deborah O'Neill |  |  |
|  | Ray Wyman, MD | Joy Burkhardt, CCRP | Torrance, CA | Torrance Memorial Medical Center (4) |
|  |  | Suellen Hosino, RN, BSN, CCRP |  |  |
|  |  |  |  |  |
|  |  | Oksana A. Lubyanaya, BA | Santa Ana, CA | Coastal Heart Medical Group (4) |
|  |  | Jose D. Salas, BS |  |  |
|  | Amer Zarka, MD | Maria Aguirre |  |  |
|  | Anil V. Shah, MD | Manu Dhawan |  |  |
|  |  | Diana Parra |  |  |
|  |  | Tri Tran |  |  |
|  | Thomas Haldis, DO | Catherine Weick, BSRT(R)(VI) | Fargo, ND | Sanford Health (4) |
|  |  | Katie Fowler-Lehman, BSN |  |  |
|  |  | Natalie Spitzer, BSN |  |  |
|  |  | Casey Riedberger |  |  |
|  |  | Catherine Weick |  |  |
|  | Jeffrey A. Kohn, MD | Stanley E. Cobos, BA | New York, NY | NYU New York Medical Associates (4) |
|  |  | Raven R. Dwyer, MPH |  |  |
|  |  | Dalisa Espinosa, MBS |  |  |
|  |  | Kirsten J. Quiles, MS |  |  |
|  | Saket Girotra, MD | Carrie Drum, RN | Iowa City, IA | University of Iowa Hospitals and Clinics (4) |
|  |  | Kimberly Miller-Cox, RN |  |  |
|  |  | Amy Ollinger, RN |  |  |
|  | Omar Almousalli, MD | Elizabeth Capasso-Gulve | Fairview Heights, IL | Advanced Heart Care Group (4) |
|  |  | Alaine Melanie Loehr |  |  |
|  |  | Marlowe Mosley |  |  |
|  | Mayil S. Krishnam, MD | Shirin Heydari, MS | Orange, CA | University of California Irvine Medical Center (3) |
|  | Jeffrey C. Milliken, MD | Andrea M. Lundeen, MA |  |  |
|  | Pranav M. Patel, MD | Edgar Karanjah, MD |  |  |
|  | Arnold H. Seto, MD | Wanda C. Marfori, MD |  |  |
|  | Kevin T. Harley, MD | Eduardo Hernandez-Rangel, MD |  |  |
|  | Michael A. Gibson, MD | Pam Singh |  |  |
|  | Byron J. Allen, MD |  |  |  |
|  | Rita Coram, MD | Anne Marie Webb, BSN | Louisville, KY | University of Louisville (3) |
|  |  | Ellie Fridell, BS |  |  |
|  |  | Heidi Wilson, BS |  |  |
|  | Sabu Thomas, MD, MSc | Angela Kim, BS | Rochester, NY | University of Rochester (3) |
|  | Ronald G Schwartz, MD, MS | Patrick Wilmot, BS |  |  |
|  | Wei Chen, MD, MS |  |  |  |
|  | Mahfouz El Shahawy, MD | Ramona Stevens | Sarasota, FL | Cardiovascular Center of Sarasota (3) |
|  | James Stafford, MD | Loriane Black | Baltimore, MD | University of Maryland Medical Center (3) |
|  | William B. Abernethy, MD | Amber B. Hull, RN | Asheville, NC | Asheville Cardiology Associates (3) |
|  |  | Olivia J. Lim, RN |  |  |
|  |  | Helen C. Tucker |  |  |
|  |  | Natasha C. Putnam, RN |  |  |
|  |  | Linda L. Hall |  |  |
|  |  | Tia Cauthren |  |  |
|  |  | Trish Tucker |  |  |
|  | Andrew Zurick, MD | Hollie Horton | Nashville, TN | Saint Thomas Hospital (3) |
|  |  | Jan Orga |  |  |
|  | Thomas M. Meyer, MD | Joyce R. White, MSN NP-C | Lynchburg, VA | Stroobants Cardiovascular Center (3) |
|  | Ronald G. Morford, MD | Cynthia Baumann, RN |  |  |
|  | Bruce Rutkin, MD | Vidya Seeratan | Manhasset, NY | Northwell Health - Manhasset (3) |
|  | Sabahat Bokhari, MD | Magnolia Jimenez | New York, NY | Columbia University Medical Center (3) |
|  | Seth I. Sokol, MD | Cidney Schultz, RN | Bronx, NY | Jacobi Medical Center (3) |
|  | Jay Meisner, MD | Jeanne Russo, RN |  |  |
|  | Ihab Hamzeh, MD |  | Houston, TX | Baylor College of Medicine (3) |
|  | Arunima Misra, MD | Zohra Huda, RN, BSN, CCRP |  |  |
|  | Matthew Wall Jr., MD | Araceli Boan |  |  |
|  | Veronica Lenges De Rosen, MD |  |  |  |
|  | Mahboob Alam, MD |  |  |  |
|  | Michael C. Turner, MD | Christine R Hinton | Lake Charles, LA | Cardiovascular Specialists of Southwest Louisiana (3) |
|  | Thomas J. Mulhearn, MD |  |  |  |
|  | Arnold P. Good, MD | Beth A. Archer, BSN, RN | Columbus, OH | Ohio Health Grant Medical Center (3) |
|  |  | Julia S. Dionne, BA |  |  |
|  |  | Cheryl A. Allardyce, BSN, RN |  |  |
|  |  | Lindsey N. Sikora, BSN, RN |  |  |
|  |  | Jennifer H. Czerniak, RN |  |  |
|  |  | Jennifer A. Mull, MSN, RN |  |  |
|  |  | Elizabeth Ferguson |  |  |
|  |  | Frances Laube |  |  |
|  | Nicolas W. Shammas, MD, MS | Gail A Shammas, BSN, RN | Davenport, IA | Midwest Cardiovascular Research Foundation (3) |
|  |  | Lori Christensen |  |  |
|  |  | Holly Park |  |  |
|  | Robert Chilton, MD | Joan Hecht | San Antonio, TX | Audie Murphy V.A. (2) |
|  | Patricia K. Nguyen, MD | Davis Vo, BS | Palo Alto, CA | VA Palo Alto Healthcare System (2) |
|  |  | James Hirsch |  |  |
|  | Matthew Jezior, MD | Jody Bindeman | Bethesda, MD | Walter Reed National Military Medical Center (2) |
|  |  | Sara Salkind |  |  |
|  |  | Dalisa Espinosa, MBS | Providence, RI | Miriam Hospital (2) |
|  |  | Lori-Ann Desimone, BSN |  |  |
|  | Paul C. Gordon, MD | Lina Felix-Stern |  |  |
|  | Thomas Crain, MD | Jassira Gomes |  |  |
|  |  | Catherine Gordon, BSN |  |  |
|  | Robert Stenberg, MD | Aimee Mann | Johnstown, PA | Conemaugh Valley Memorial Hospital (2) |
|  |  | Theresa McCreary |  |  |
|  | Ronald P. Pedalino, MD | Stanley E. Cobos, BA | Brooklyn, NY | NYU-HHC Kings County Hospital Center (2) |
|  |  | Raven R. Dwyer, MPH |  |  |
|  |  | Dalisa Espinosa, MBS |  |  |
|  |  | Kirsten J. Quiles, MS |  |  |
|  | Joseph Wiesel, MD | Stanley E. Cobos, BA | Flushing, NY | New York University - Langone Cardiovascular Associates (2) |
|  |  | Raven R. Dwyer, MPH |  |  |
|  |  | Dalisa Espinosa, MBS |  |  |
|  |  | Kirsten J. Quiles, MS |  |  |
|  | George J. Juang, MD | Candace Gopaul, BS | Brooklyn, NY | Coney Island Hospital (2) |
|  |  | Karen Hultberg |  |  |
|  |  | Tauqir Huk |  |  |
|  |  | Afshan Hussain |  |  |
|  | Mohammed Al-Amoodi, MD | Yesenia Zambrano, BS | Yuma, AZ | Yuma Regional Medical Center (2) |
|  |  | Sarah Medina Rodriguez |  |  |
|  |  | Trudie Milner |  |  |
|  | David Wohns, MD | Abbey Mulder, RN | Grand Rapids, MI | Spectrum Health (2) |
|  |  | Stacie Van Oosterhout, MEd |  |  |
|  | Ellis W. Lader, MD | Martha Meyer, RN, MSN | Kingston, NY | Mid Valley Cardiology (1) |
|  | Michael Mumma, MD | Nancy L. Clapp, RN, BA, CCRC | Sarasota, FL | Sarasota Memorial Hospital (1) |
|  |  | Heather Barrentine |  |  |
|  | Lekshmi Dharmarajan , MD | Jenne M. Jose, PA | Bronx, NY | NYU-HHC Lincoln Medical and Mental Health Center (1) |
|  |  | Stanley E. Cobos, BA |  |  |
|  |  | Raven R. Dwyer, MPH |  |  |
|  |  | Dalisa Espinosa, MBS |  |  |
|  |  | Kirsten J. Quiles, MS |  |  |
|  |  | Jenne Manchery |  |  |
|  | Joseph F.X. McGarvey Jr, MD | Vera McKinney, RN | Doylestown, PA | Doylestown Health Cardiology (1) |
|  |  | Linda Schwarz, RN |  |  |
|  | Thomas R. Downes, MD (till Dec. 2016) | Scott M. Kaczkowski | Loveland, CO | Medical Center of the Rockies (1) |
|  | Gary J. Luckasen, MD (from Dec. 2016) | Adam J. Jaskowiak |  |  |
|  |  | Joel Klitch |  |  |
|  | Benjamin Cheong, MD | Debra Dees | Houston, TX | Baylor St. Luke's Medical Center (1) |
|  | Srinivasa Potluri, MD | Precilia Vasquez | Plano, TX | Baylor Research Institute at Legacy Heart Center (1) ** |
|  | Ronald A. Mastouri, MD |  | Indianapolis, IN | Indiana University/Krannert Institute of Cardiology (1) |
|  | Jeffery A. Breall, MD, PhD | Elise L. Hannemann, RN,CCRC |  |  |
|  | George E. Revtyak, MD | Judy Mae Foltz, RN,CCRC |  |  |
|  | Jonathan W. Bazeley, MD |  |  |  |
|  | Dayuan Li, MD | Emily DeRosa | St. Paul, MN | HealthEast Saint Joseph's Hospital (1) |
|  |  | Beth Jorgenson |  |  |
|  |  | Joyce Riestenberg-Smith |  |  |
|  | Kenneth Giedd, MD |  | New York, NY | Beth Israel Medical Center (1) |
|  | Wayne Old, MD | Rebecca Bariciano | Chesapeake, VA | Cardiovascular Associates, Ltd. (1) |
|  | Francis Burt, MD |  | Bethlehem, PA | Saint Luke's Hospital and Health Network (1) |
|  | Kozhaya Sokhon, MD | Jessica Waldron | Sugar land, TX | Medicus Alliance Clinical Research Org., Inc. (1) |
|  |  | Michelle Mayon |  |  |
|  | Deepika Gopal, MD |  | Plano, TX | The Heart Hospital Baylor (1) |
|  | Uma S. Valeti, MD | Gretchen Ann Peichel, RN | Minneapolis, MN | University of Minnesota (1) |
|  | Jon Kobashigawa, MD | Brandy Starks | Beverly Hills, CA | Cedars Sinai Medical Center (1) |
|  |  | Lucilla Garcia |  |  |
|  |  | Maria Thottam |  |  |
| India (941) |  |  |  |  |
| Country Leader |  |  |  |  |
| Balram Bhargava, DM |  |  |  |  |
|  |  | Anjali Anand, MSc | Calicut | Government Medical College (208) |
|  | Sajeev Chakanalil Govindan, MD, DNB, DM, PhD | Janitha Raj, B.Tech |  |  |
|  | Rajesh Gopalan Nair, MD, DNB, DM | Reshma Ravindran, MSc |  |  |
|  |  | Rajalekshmi VS, MSc, MScCRRA |  |  |
|  | Cholenahally Nanjappa Manjunath, MD, DM | Nandita Nataraj, BE(Biotech) PGDICRCDM | Bengaluru | Sri Jayadeva Institute of Cardiovascular Sciences and Research (149) |
|  | Nagaraja Moorthy, MD, DM | Soundarya Nayak, BE(Biotech) PGDICRCDM | |  |
|  | Satvic Cholenahally Manjunath, MD,DM | Mahevamma Mylarappa, GNM (General Nursing) | |  |
|  | Suryaprakash Narayanappa, MBBS |  |  |  |
|  | Neeraj Pandit, MD, DM | Sheromani Bajaj | New Delhi | Dr Ram Manohar Lohia Hospital (101) |
|  | Ranjit Kumar Nath, MD, DM | Vandana Yadav, Msc,PGDACR |  |  |
|  |  | Girish Mishra, Msc, PGDACR |  |  |
|  | S.K. Dwivedi, DM | Roma Tewari, PG | Lucknow | King George's Medical University, Department of Cardiology (100) |
|  | V.S. Narain, DM | Meenakshi Mishra, PG |  |  |
|  | Sharad Chandra, DM | Shivali Patel |  |  |
|  |  | Suman Singh, PG |  |  |
|  | Gurpreet S. Wander, DM |  | Ludhiana | Hero DMC Heart Institute, Dayanand Medical College and Hospital (83) |
|  | Rohit Tandon, MD |  |  |  |
|  | Sarju Ralhan, M.Ch (CTVS) | Baljeet Kaur, MSc (Biotechnology) |  |  |
|  | Naved Aslam, DM | Sonika Gupta , MBA, B. Pharmacy |  |  |
|  | Abhishek Goyal, DM |  |  |  |
|  | Balram Bhargava, DM | Chandini Suvarna, BDS | New Delhi | All India Institute Of Medical Sciences (67) |
|  | G.Karthikeyan, DM |  |  |  |
|  | S.Ramakrishnan, DM |  |  |  |
|  | Sandeep Seth, DM |  |  |  |
|  | Rakesh Yadav, DM |  |  |  |
|  | Sandeep Singh, DM |  |  |  |
|  | Ambuj Roy, DM |  |  |  |
|  | Neeraj Parakh, DM |  |  |  |
|  | Sunil Kumar Verma, DM |  |  |  |
|  | Rajiv Narang, DM |  |  |  |
|  | Sundeep Mishra, DM |  |  |  |
|  | Nitish Naik, DM |  |  |  |
|  | Gautam Sharma, DM |  |  |  |
|  | Shiv Kumar Choudhary, M.Ch |  |  |  |
|  | Chetan Patel, DNB |  |  |  |
|  | Gurpreet Gulati, MD |  |  |  |
|  | Sanjeev Sharma, MD |  |  |  |
|  | V K Bahl, DM |  |  |  |
|  | Anoop Mathew, MD | Binoy Mannekkattukudy Kurian | Kolenchery | MOSC Medical College Hospital (39) |
|  | Eapen Punnoose, MD |  |  |  |
|  | Milind Avdhoot Gadkari, MD | Sheetal Rupesh Karwa, BHMS | Pune | KEM Hospital Pune (35) |
|  | Siddharth Gadage, MD DNB | Suvarna Kolhe, MSc |  |  |
|  | Tapan Umesh Pillay, BHMS MSc |  |  |  |
|  | Santhosh Satheesh, MBBS, MD, DM | R. J. Vindhya, B.Sc. (Bio-Technology), MSc(Bio-Informatics) | Pondicherry | Jawaharlal Institute of Postgraduate Medical Education & Research (JIPMER) (31) |
|  |  | Peeyush Jain, MD | New Delhi | Fortis Escort Heart Institute |
|  |  | Ashok Seth, MD |  | -31 |
|  |  | Zile Singh Meharwal, MD |  |  |
|  | Atul Mathur, MD | Atul Verma, MD |  |  |
|  | Upendra Kaul, MD | Mona Bhatia, MD |  |  |
|  |  | Ankush Sachdeva, MD |  |  |
|  |  | Thounaojam Indira Devi, RN |  |  |
|  |  | Nungshi Jungla, RN |  |  |
|  | Johann Christopher, MD, DNB | K. Manjula Rani, MSc. | Hyderabad | Gurunanak CARE Hospital (27) |
|  | Rajeev Menon, MD, DNB | M. Sowjanya Reddy, BSc |  |  |
|  | Nirmal Kumar, MD, DNB | K. Preethi, BSc |  |  |
|  | Abraham Oomman, MD,DM,DNB | Rinu R sidh, MSc(Clinical Research) | Chennai | Apollo Research and Innovation (23) |
|  | Robert Mao, MD, DM | Ramakrishnan T., B.Tech(Biotechnology) | |  |
|  | Hilda Solomon, PhD | Rajesh Francis, MSc(Clinical Research) | |  |
|  | Sudhir Naik, MD, DM | Vamshi Priya P., MSc | Hyderabad | Apollo Research & Innovations (13) |
|  | Sajeeda Parveen Khan, MBBS, (Dip.Card) |  |  |  |
|  | Johann Christopher, MD | Kotiboinna Preethi | Hyderabad | CARE Nampally (11) |
|  | Nirmal Kumar, MD |  |  |  |
|  | Purvez Grant, MD | Shweta Hande, BHMS, PGDCR | Pune | Ruby Hall Clinic,Grant Medical Foundation (10) |
|  |  | Poonam Sonawane, B.ScMicrobiology, ACCR | |  |
|  | Ranjan Kachru, MD | Abhishek Dubey | New Delhi | Fortis Healthcare Fl.t Lt. Rajan Dhall Hospital (4) |
|  |  | Kavita Rawat |  |  |
|  | Ajit Kumar VK, MD, DM |  | Trivandrum | Sree Chitra Tirunal Institute for Medical Sciences and Technology (3) |
|  | Sanjay Ganapathi, MD, DM |  |  |  |
|  | Jayakumar K, MS, M.Ch | Vineeth CP |  |  |
|  | Harikrishnan Sivadasanpillai, MD, DM | Manas Chacko, RN |  |  |
|  | Bijulal Sasidharan, MD, DM | Suresh Babu |  |  |
|  | Kapilamoorthy TR, MD |  |  |  |
|  | Johann Christopher, MD | Sowjanya Reddy | Hyderabad | CARE Hospital (3) |
|  | Praneeth Polamuri, MD | Manjula Rani |  |  |
|  | Upendra Kaul, MD | Priyadarshani Arambam | New Delhi | Batra Hospital and Medical Research Centre (BHMRC) (3) |
|  |  | Bebek Singh |  |  |
| United Kingdom (539) |  |  |  |  |
| Country Leaders |  |  |  |  |
| Roxy Senior, MBBS, MD, DM |  |  |  |  |
| Keith AA Fox, MBChB *(past)* |  |  |  |  |
| Country Coordinators |  |  |  |  |
| Grace M. Young , MSc, BSc (Hons) | |  |  |  |
| Kathryn Carruthers *(past)* |  |  |  |  |
|  | Roxy Senior, MBBS, MD, DM |  | Harrow | Northwick Park Hospital Harrow/ Royal Brompton Hospital London (202) |
|  | Ahmed Elghamaz, MB BCh |  |  |  |
|  | Sothinathan Gurunathan, MBChB |  |  |  |
|  | Nikolaos Karogiannis, MBBS | Grace M. Young , MSc, BSc (Hons) |  |  |
|  | Benoy N Shah, MD, MBBS, BSc (Hons) | Christopher Kinsey |  |  |
|  | Richard HJ Trimlett, MBBS, CCST | Raisa Kavalakkat, MSc, BSc, RN |  |  |
|  | Michael B Rubens, LRCP, MRCS, MBBS, DMRD | Jo Evans, RN |  |  |
|  | Edward D Nicol, MD, BMedSci, MBBS, DTM&H | Ikraam Hassan, RN |  |  |
|  | Tarun K Mittal, MD |  |  |  |
|  | Reinette Hampson, BSc (Hons), BA (Hons) | |  |  |
|  | Reto Andreas Gamma, MBBS | Sarah Williams, RN | Chelmsford | Broomfield Hospital (39) |
|  |  | Kim Holland, RN |  |  |
|  |  | Karen Swan, RN |  |  |
|  | Mark A de Belder, MD | Bev Atkinson, RN | Middlesbrough | The James Cook University Hospital, Middlesbrough (37) |
|  | Jeet Thambyrajah, MD |  |  |  |
|  | Thuraia Nageh, BSc(Hons) MBBS MD MRCP | Swapna Kunhunny, MRes Clin Res, BSc (N), RN | Westcliffe on Sea | Southend University Hospital (34) |
|  | John R Davies, MBBS, PhD |  |  |  |
|  | Steven J. Lindsay, MD | Craig Atkinson, RN | Bradford | Bradford Royal Infirmary (20) |
|  | John Kurian, MD | Carita Krannila, RN |  |  |
|  | Haqeel Jamil, MD | Manitha Vinod, RN |  |  |
|  | Osama Raheem, MD |  |  |  |
|  | Angela Hoye, MD | Lisa Chaytor | Cottingham | The University of Hull/Castle Hill Hospital (19) |
|  |  | Leanne Cox |  |  |
|  |  | Julie Morrow |  |  |
|  |  | Kay Rowe |  |  |
|  | Patrick Donnelly, MD | Stephanie Kelly, RN | Belfast | South Eastern Health and Social Care (17) |
|  | Bernardas Valecka, MD | Susan Regan, RN |  |  |
|  |  | Dawn Turnbull |  |  |
|  | Anoop Chauhan, MD | Catherine Fleming | Blackpool | Blackpool Teaching Hospitals (16) |
|  |  | Arijit Ghosh |  |  |
|  |  | Karen Gratrix |  |  |
|  |  | Stephen Preston |  |  |
|  | Craig Barr, MD | Anne Cartwright | Dudley | Russells Hall Hospital (15) |
|  | Khaled Alfakih, MBBS, MD | Abigail Knighton, BSc., PG Dip. | London | King's College NHS Foundation Hospital (14) |
|  | Jonathan Byrne, PhD | Katherine Martin, RGN, Dip. N, MSc |  |  |
|  | Ian Webb, PhD, MA |  |  |  |
|  | Peter Henriksen, PhD, MB ChB, BSc(Hons) | Laura Flint, RGN | Edinburgh | Royal Infirmary of Edinburgh (13) |
|  |  | James Harrison, BSc(Hons), PG dip |  |  |
|  | Peter OKane, MD | Nicki Lakeman | Bourneouth | Royal Bournemouth Hospital (13) |
|  |  | Anja Ljubez |  |  |
|  | Ramesh de Silva, MB ChB, MD |  | Bedford | Bedford Hospital NHS Trust (11) |
|  | Dwayne S. G. Conway, MD | Judith Wright | Wakefield | Pinderfields Hospital (11) |
|  |  | Donna Exley |  |  |
|  | Alexander A Sirker, MB BChir, PhD |  | London |  |
|  |  | Mervyn Andiapen, RN |  | University College London Hospitals NHS Foundation Trust |
|  |  | Amy J. Richards, BSc |  | BartsHealth NHS Trust |
|  |  |  |  |  |
|  |  |  |  | -11 |
|  | Stephen P Hoole, MD | Lisa Wong, MSc | Cambridge | Papworth Hospital (10) |
|  | Fraser N. Witherow, MD | Melanie J. Munro, RGN | Dorchester | Dorset County Hospital (8) |
|  | Nicola Johnston, MB, Bch BAO, MRCP, MD | | Belfast | Belfast Trust (7) |
|  | Mark Harbinson, MB, Bch BAO, MRCP, MD | Michelle McEvoy, RN |  |  |
|  | Simon Walsh, MB, Bch BAO, MD | Caroline Brown, RN |  |  |
|  | Hanna Douglas, MB, Bch BAO, MRCP, MD | |  |  |
|  | Matthew Luckie, MD | Thabitha Charles | Manchester | Central Manchester University Hospital (7) |
|  |  | Laurel Kolakaluri |  |  |
|  |  | Hannah Phillips |  |  |
|  | Jolanta Sobolewska, MD | Louise Morby, RN | Oldham | The Pennine Acute Hospitals NHS Trust (6) |
|  |  | Karen Hallett, RN |  |  |
|  |  | Carolyn Corbett, RN |  |  |
|  |  | Lynne Winstanley |  |  |
|  | Paramjit Jeetley, MD | Angelique Smit, RN | London | Royal Free London NHS Foundation Trust (6) |
|  | Niket Patel, MD |  |  |  |
|  | Tushar Kotecha, MBChB, Mpharm |  |  |  |
|  | Christopher Travill, MBBS, MD | Susan Gent, SRN RGN | Luton | Luton and Dunstable University Hospital NHS FT (5) |
|  | Iqbal Karimullah, MBBS | Nafisa Hussain, BSc |  |  |
|  | Mahmud Al-Bustami, MBBS |  |  |  |
|  | Denise Braganza, MD | Fiona Haines | Peterborough | Peterborough City Hospital (5) |
|  |  | Joanne Taaffe |  |  |
|  | Robert Henderson, MD | Jane Burton | Nottingham | Nottingham University Hospitals (4) |
|  | Kate Pointon, MBBS | Maria Colton |  |  |
|  | Surendra Naik, PhD | Rachel King |  |  |
|  | Thomas Mathew, MBBS, MD, DM |  |  |  |
|  |  | Ammani Brown, MSc BA RN | Clydebank | University of Glasgow (4) |
|  |  | Andrew Docherty, RN |  |  |
|  | Colin Berry, BSc MB ChB, PhD | Lisa McCloy, RN |  |  |
|  | Damien Collison, MB ChB | Kate Robb, RN |  |  |
|  | Giles Roditi, MB ChB | Craig Paterson, PhD |  |  |
|  |  | Wenda Crawford, RN |  |  |
|  |  | Joanne Kelly, RN |  |  |
|  |  | Lorraine McGregor, RN |  |  |
|  | Andrew J Moriarty, BSc MB PhD | Anne Mackin, RN, BSc | Craigavon | Cardiovascular Research Unit, Craigavon Area Hospital (2) |
|  | Jason D. Glover, MBBS | Janet P Knight, RN | Basingstoke | Hampshire Hospitals NHS Foundation Trust (2) |
|  | Jiwan Pradhan, MBBS |  |  |  |
|  | Ghada Mikhail, MD | Tuhina Bose | London | Imperial College Healthcare NHS Trust (1) |
|  | Darrel P. Francis, MD, MA |  |  |  |
| *Canada (447) |  |  |  |  |
| Country Leaders |  |  |  |  |
| Vladimir Dzavik, MD |  |  |  |  |
| Shaun Goodman, MD, MSc |  |  |  |  |
| Gilbert Gosselin, MD |  |  |  |  |
|  | Gilbert Gosselin, MD | Anna Proietti, RN | Montreal, QC | Montreal Heart Institute (90) |
|  |  | Myriam Brousseau, RN |  |  |
|  |  | Magalie Corfias, RN |  |  |
|  |  | Patricia Blaise |  |  |
|  |  | Luc Harvey |  |  |
|  | Ariel Diaz, MD |  | Trois-Rivieres, QC | Centre Hospitalier de Regional Trois-Rivieres (71) |
|  | Philippe Rheault, MD |  |  |  |
|  | Miguel Barrero, MD |  |  |  |
|  | Carl-Éric Gagné, MD | Patricia Alarie |  |  |
|  | Yanek Pépin-Dubois, MD | Linda Arcand |  |  |
|  | Ricardo Costa, MD | Isabelle Roy |  |  |
|  | Ying Tung Sia, MD | Estelle Montpetit |  |  |
|  | Catherine Lemay, MD |  |  |  |
|  | Alejandro Gisbert, MD |  |  |  |
|  | Pierre Gervais, MD |  |  |  |
|  | Alain Rheault, MD |  |  |  |
|  |  | Katia Drouin, RN | Terrebonne, QC | CISSSL - Hopital Pierre-Le Gardeur (42) |
|  | Denis Carl Phaneuf, MD | Christine Bergeron, RN |  |  |
|  | Gilbert Gosselin, MD | Christine Shelley |  |  |
|  |  | Christine Masson |  |  |
|  | Pallav Garg, MBBS, MSc | Sandy Carr, RN | London, ON | London Health Sciences Centre (35) |
|  |  | Catherine Bone, RN |  |  |
|  | Benjamin J.W. Chow, MD | Ermina Moga | Ottawa, ON | University of Ottawa Heart Institute (29) |
|  | Renee C. Hessian, MD | Janetta Kourzenkova |  |  |
|  | Rob S. Beanlands, MD | Olga Walter |  |  |
|  | Richard F. Davies, MD |  |  |  |
|  | Kevin R. Bainey, MD, MSc | Norma Hogg, RN | Edmonton, AB | University of Alberta (28) |
|  |  | Suzanne Welsh, RN |  |  |
|  | Asim N. Cheema, MD, PhD |  | Toronto, ON | St. Michael's Hospital (27) |
|  | Akshay Bagai, MD, MHS |  |  |  |
|  | Ron Wald, MDCM, MPH |  |  |  |
|  | Shaun Goodman, MD, MSc | Khrystyna Kushniriuk, HBSc, MD |  |  |
|  | John Joseph Graham, MRCP, MB ChB, BSc | Mohammed Hussain |  |  |
|  | Mark Peterson, MD, FRCSC, PhD | Olugbenga Bello |  |  |
|  | Chi-Ming Chow, MD, CM, MSc |  |  |  |
|  | Beth Abramson, MD, MSc |  |  |  |
|  | Asim Nazir Cheema, MD | Ishba Syed, MBBS | Mississauga, ON | Dixie Medical Group (24) |
|  | Mohammad Tariq Vakani, MD | Mohammed Hussain, BSc(H) |  |  |
|  |  | Khrystyna Kushniriuk, MBBS |  |  |
|  | James Cha, MD | Judy Otis, CRC | Oshawa, ON | Dr. James Cha (21) |
|  |  | Rebecca Otis, CRC |  |  |
|  | Andrew G Howarth, MD, PhD | Michelle M Seib, RN | Calgary, AB | University of Calgary (15) |
|  |  | Sandra M Rivest, RN |  |  |
|  |  | Rosa Sandonato, BSCN |  |  |
|  | Graham Wong, MD | Jackie Chow | Vancouver, BC | Vancouver General Hospital (15) |
|  |  | Andrew Starovoytov |  |  |
|  |  | Naomi Uchida |  |  |
|  |  | Ngaire Meadows |  |  |
|  | Amar Uxa, MD | Nadia Asif | Toronto, ON | University Health Network (14) |
|  |  | Suzana Tavares |  |  |
|  | Paul Galiwango, MD | Bev Bozek, RN, CCRC | Scarborough, ON | Scarborough Cardiology Research (9) |
|  | Saleem Kassam, MD | Maria Shier |  |  |
|  | Ashok Mukherjee, MD | Lori-Ann Larmand |  |  |
|  | A. Joseph Ricci, MD | Amir Janmohamed |  |  |
|  |  | Brenda Hart |  |  |
|  | Andy Lam, MD | Jane Marucci | East Grimsby, ON | West Lincoln Memorial Hospital (8) |
|  |  | Sharon Tai |  |  |
|  | Shamir Mehta, MD | Sonya Brons, RN | Hamilton, ON | Hamilton General Hospital (7) |
|  |  | Chris Beck, RN |  |  |
|  |  | Glenda Wong, RN |  |  |
|  |  | Krystal Etherington |  |  |
|  |  | Thippeekaa Arumairajah |  |  |
|  | Jacob Udell, MD | Maria Aprile | Toronto, ON | Women's College Hospital (7) |
|  |  | Sara Karlsson |  |  |
|  |  | Susan Webber |  |  |
|  | Philippe Généreux, MD | Chantale Mercure | Montréal, QC | Centre Intégré Universitaire de Santé et de Services Sociaux du Montréal (2) |
|  | Adnan Hameed, MD | Nancy Aedy | St. Catharines, ON | Saint Catharines General Hospital (2) |
|  |  |  |  |  |
|  | Ledjalem Daba, MD | Fran Farquharson | Vaughan, ON | Northwest GTA Cardiovascular and Heart Rhythm Program (1) |
|  |  | Anam Siddiqui |  |  |
| Brazil (399) |  |  |  |  |
| Country Leaders |  |  |  |  |
| Antonio Carlos Carvalho, MD, PhD | |  |  |  |
| Renato D. Lopes, MD, PhD |  |  |  |  |
|  | Whady Hueb, MD | Myrthes Emy Takiuti, RN | Sao Paulo | Heart Institute (InCor) University of São Paulo (127) |
|  | Paulo Cury Rezende, MD |  |  |  |
|  | Expedito Eustáquio Ribeiro Silva, MD |  |  |  |
|  | Alexandre Ciappina Hueb, MD |  |  |  |
|  | Paola Emanuela Poggio Smanio, MD, PhD | Leonardo Pizzol Caetano, PhD | São Paulo | Instituto Dante Pazzanese de Cardiologia (98) |
|  | Alexandre Schaan de Quadros, MD |  | Porto Alegre | Instituto de Cardiologia de Porto Alegre (41) |
|  | Renato Abdala Karam Kalil, MD | Aline Peixoto Deiro |  |  |
|  | José Luiz da Costa Vieira, MD | Alice Manica Muller |  |  |
|  | Gabriel Grossmann , MD | Maria Antonieta Pereira de Moraes |  |  |
|  | Pedro Píccaro de Oliveira, MD | Bruna Maria Ascoli |  |  |
|  | Leonardo Bridi, MD | Sílvia Zottis Poletti |  |  |
|  | Simone Savaris, MD |  |  |  |
|  | João V Vitola, MD, PhD |  | Curitiba | Quanta Diagnostico & Terapia (33) |
|  | Rodrigo J Cerci, MD, Msc | Sandra S. Zier, BSc |  |  |
|  | Fabio R Farias, MD, Msc | Vilmar Veiga Jr, BSc |  |  |
|  | Miguel M Fernandes, MD, PhD |  |  |  |
|  | José Antonio Marin-Neto, MD, PhD |  | Ribeirao Preto | Hospital das Clinicas da Faculdade de Medicina de Ribeirão Preto da Universidade de São Paulo (31) |
|  | André Schmidt, MD, PhD |  |  |  |
|  | Moysés de Oliveira Lima Filho, MD, PhD | Diego Franca da Cunha |  |  |
|  | Ricardo Mendes Oliveira, MD |  |  |  |
|  | João Reynaldo Abbud Chierice, MD |  |  |  |
|  | Carísi A. Polanczyk, MD | Guilherme G Rucatti, PsyD | Porto Alegre | Hospital de Clínicas de Porto Alegre |
|  | Mariana V. Furtado, MD | Fernanda Igansi, BSc |  | -12 |
|  | Luis F. Smidt, MD | Mauren P Haeffner, BSc |  |  |
|  | Antonio Carlos Carvalho, MD | Viviane Almeida | Sao Paulo | Unifesp - Hospital Sao Paulo (9) |
|  | Gustavo Pucci, MD | Gabriela Sanchez de Souza |  |  |
|  | Flavio Lyra, MD |  |  |  |
|  | Alvaro Rabelo Alves Junior, MD | Mayana Almeida | Salvador | Fundacao Bahiana de Cardilogia (9) |
|  |  | Viviane dos Santos |  |  |
|  | Marianna D. A. Dracoulakis, MD, PhD | Natalia S Oliveira, RN | Salvador | Hospital da Bahia (8) |
|  | Rodolfo G. S. D Lima, MD |  |  |  |
|  | Estevao Figueiredo, MD | Bruna Edilena Paulino Azevedo | Belo Horizonte | Hospital Lifecenter (8) |
|  | Paulo Ricardo Caramori, MD | Marco Bizzaro Santos | Porto Alegre | Hospital Sao Lucas da Pontificia Universidade Catolica do Rio Grande do Sol (7) |
|  |  | Amanda Germann |  |  |
|  |  | Vitor Gomes |  |  |
|  |  | Rosa Homem |  |  |
|  |  | Ellen Magedanz |  |  |
|  | Rogerio Tumelero, MD | Rosane Laimer | Fundo | Hospital Sao Vicente de Paulo (5) |
|  |  | Alexandre Tognon |  |  |
|  | Frederico Dall’Orto, MD |  | Pocos de Caldas | Hospital Maternidade e Pronto Socorro Santa Lucia (4) |
|  | Claudio T. Mesquita, MD | Roberta P Santos, RN | Botafogo | Hospital Pró-Cardíaco (3) |
|  | Alexandre S. Colafranseschi, MD |  |  |  |
|  |  |  |  |  |
|  | Amarino C. Oliveira Jr., MD |  |  |  |
|  | Luiz A. Carvalho, MD |  |  |  |
|  | Isabella C. Palazzo, MD |  |  |  |
|  | Andre S. Sousa, MD |  |  |  |
|  | Expedito Eustáquio Ribeiro da Silva, MD, PhD | | Sao Paulo | Hospital TotalCor (2) |
|  | Pedro Gabriel Melo de Barros e Silva, MD, PhD | Mariana Yumi Okada, RN |  |  |
|  | Luciana de Pádua Silva Baptista, MD, PhD | Ana Paula Batista, RN |  |  |
|  | Marcelo Jamus Rodrigues, MD | Aline Nogueira Rabaça, BS |  |  |
|  | Marcos Valério Coimbra de Resende, MD, PhD | |  |  |
|  | Jose Francisco Saraiva, MD | Larissa Miranda Trama | Sao Paulo | Hospital Celso Pierro (1) |
|  |  | Talita Silva |  |  |
|  |  | Camila Thais de Souza Ormundo |  |  |
|  |  | Carla Vicente |  |  |
|  | Costantino Costantini, MD, PhD | Caroline Pinheiro | Curitiba | Hospital Cardiologico Costantini (1) |
|  |  | Daniele Komar |  |  |
| Poland (333) |  |  |  |  |
| Country Leaders |  |  |  |  |
| Witold Ruzyllo, MD |  |  |  |  |
| Hanna Szwed, MD, PhD |  |  |  |  |
| Country Coordinator |  |  |  |  |
| Radoslaw Pracon, MD, PhD |  |  |  |  |
|  | Marcin Demkow, MD, PhD |  | Warsaw | Coronary and Structural Heart Diseases Department, Institute of Cardiology (127) |
|  | Radoslaw Pracon, MD, PhD |  |  |  |
|  | Cezary Kepka, MD PhD |  |  |  |
|  | Anna Teresinska, MD PhD | Olga Walesiak |  |  |
|  | Karolina Kryczka, MD PhD | Katarzyna Malinowska |  |  |
|  | Jan Henzel, MD PhD |  |  |  |
|  | Mateusz Solecki, MD PhD |  |  |  |
|  | Edyta Kaczmarska, MD PhD |  |  |  |
|  | Tomasz Mazurek, MD, PhD | Jakub Maksym, MD | Warszawa | Medical University of Warsaw (48) |
|  |  | Karolina Wojtera, MD |  |  |
|  |  | Anna Fojt, MD |  |  |
|  |  | Ewa Szczerba, MD |  |  |
|  | Jaroslaw Drozdz, PhD |  | Lodz | Cardiology Clinic, Medical University in Lodz (43) |
|  | Bartosz Czarniak, MD |  |  |  |
|  | Malgorzata Frach (formerly Stasiak), MD |  |  |  |
|  | Konrad Szymczyk, MD |  |  |  |
|  | Iwona Niedzwiecka, MD |  |  |  |
|  | Sebastian Sobczak, MD |  |  |  |
|  | Tomasz Ciurus, MD |  |  |  |
|  | Piotr Jakubowski, MD |  |  |  |
|  | Magdalena Misztal-Teodorczyk, MD |  |  |  |
|  | Dawid Teodorczyk, MD | Marta Swiderek, MA |  |  |
|  | Aleksandra Fratczak, MD | Ewelina Wojtala, MA |  |  |
|  | Marcin Szkopiak, MD |  |  |  |
|  | Patrycja Lebioda, MD |  |  |  |
|  | Michal Wlodarczyk, MD |  |  |  |
|  | Anna Plachcinska, MD |  |  |  |
|  | Jacek Kusmierek, MD |  |  |  |
|  | Magdalena Miller, MD |  |  |  |
|  | Halina Marciniak, MD |  |  |  |
|  | Karolina Wojtczak-Soska, MD |  |  |  |
|  | Katarzyna Łuczak, MD |  |  |  |
|  | Tomasz Tarchalski, MD |  |  |  |
|  | Anna Cichocka-Radwan, MD |  |  |  |
|  | Hanna Szwed, MD, PhD | Jaroslaw Karwowski, MD | Warsaw | National Institute of Cardiology, Warsaw (35) |
|  | Grazyna Anna Szulczyk, MD |  |  |  |
|  | Adam Witkowski, MD, PhD |  | Warsaw | Department of Interventional Cardiology & Angiology, Institute of Cardiology (20) |
|  | Krzysztof Kukuła, MD, PhD |  |  |  |
|  | Małgorzta Celińska-Spodar, MD |  |  |  |
|  | Joanna Zalewska, MD |  |  |  |
|  | Grzegorz Gajos, MD, PhD |  | Krakow | Department of Coronary Disease, John Paul II Hospital, Jagiellonian University Medical College  (16) |
|  | Krzysztof Bury, MD, PhD |  |  |  |
|  | Piotr Pruszczyk, MD, PhD | Andrzej Łabyk, MD | Warszawa | Department of Internal Medicine and Cardiology, Infant Jesus Teaching Hospital, Medical University of Warsaw (15) |
|  | Marek Roik, MD, PhD | Agnieszka Szramowska, MD |  |  |
|  |  | Olga Zdończyk, MD |  |  |
|  | Krystyna Łoboz-Grudzień, MD, PhD | Joanna Jaroch, MD, PhD | Wrocław | T.Marciniak Hospital (11) |
|  | Leszek Sokalski, MD, PhD |  |  |  |
|  | Barbara Brzezińska, MD, PhD |  |  |  |
|  | Maciej Lesiak, Professor, MD |  | Poznan | Szpital Kliniczny Przemienienia Pańskiego (10) |
|  | Magdalena Łanocha, MD |  |  |  |
|  | Krzysztof W. Reczuch, MD | Adam Kolodziej, MD | Wroclaw | Military Hospital / Medical University (4) |
|  | Zbigniew Kalarus, MD |  | Zabrze | Medical University of Silesia, School of Medicine with the Division of Dentistry, Department of Cardiology, Congenital Heart Diseases and Electrotherapy, Silesian Center for Heart Diseases (3) |
|  | Andrzej Swiatkowski, MD |  |  |  |
|  | Mariola Szulik, MD |  |  |  |
|  | Wlodzimierz J. Musial, MD | Marta Marcinkiewicz-Siemion, MD | Bialystok | University Hospital in Bialystok (1) |
| Russia (303) |  |  |  |  |
| Country Coordinator |  |  |  |  |
| Olga Bockeria, MD, PhD |  |  |  |  |
|  | Leo Bockeria, MD, PhD | Olga Bockeria, MD, PhD | Moscow | National Medical Research Center for Cardiovascuar Surgery (113) |
|  | Karen Petrosyan, MD, PhD | Zalina Kudzoeva, MD |  |  |
|  | Tatiana Trifonova, MD | Nodira Aripova, MD |  |  |
|  | Alexander M. Chernyavskiy, MD, PhD | Ivan A. Naryshkin, MD | Novosibirsk | E.Meshalkin National Medical Research Center of the Ministry of Health of the Russian Federation (101) |
|  | Evgeniy I. Kretov, MD | Alena Kuleshova, MD |  |  |
|  | Igor O. Grazhdankin, MD | Dastan Malaev, MD |  |  |
|  | Leonid L. Bershtein, MD, PhD |  | Saint Petersburg | North-Western State Medical University (50) |
|  | Sergey A. Sayganov, MD, PhD | Irina Subbotina |  |  |
|  | Anastasia M. Kuzmina-Krutetskaya, MD | Victoria Gumerova |  |  |
|  | Elizaveta V. Zbyshevskaya, MD, PhD |  |  |  |
|  | Nana O. Katamadze, MD, PhD |  |  |  |
|  | Elena A. Demchenko, MD, PhD | Olga B. Nikolaeva, MD | Saint Petersburg | Federal Almazov North-West Medical Research Centre (39) |
|  | Pavel S. Kozlov, MD |  |  |  |
|  | Vikentiy Y. Kozulin, MD |  |  |  |
|  | Ekaterina I. Lubinskaya, MD |  |  |  |
| *Spain (286) |  |  |  |  |
| Country Leader |  |  |  |  |
| Jose Luis Lopez-Sendon, MD, PhD | |  |  |  |
| Country Coordinator |  |  |  |  |
| Almudena Castro, MD |  |  |  |  |
|  | Jose Lopez-Sendon, MD, PhD | Virginia Fernández-Figares, Pharm | Madrid | Hospital La Paz. IdiPaz (118) |
|  | Almudena Castro, MD |  |  |  |
|  | Elena Refoyo Salicio, MD |  |  |  |
|  | Gabriela Guzman, MD |  |  |  |
|  | Gabriel Galeote, MD |  |  |  |
|  | Silvia Valbuena, MD |  |  |  |
|  | Jesús Peteiro, MD, PhD |  | A Coruna | Complexo Hospitalario Universitario A Coruña (CHUAC) Sergas, Department of Cardiology. INIBIC A Coruña. CIBER-CV. Universidad de A Coruña, Spain (112) |
|  | María Dolores Martínez-Ruíz, MD |  |  |  |
|  | Ruth Pérez-Fernández, MD | Moisés Blanco-Calvo, PhD |  |  |
|  | José J Cuenca-Castillo, MD | Encarnación Alonso-Álvarez, BSc |  |  |
|  | Xacobe Flores-Ríos, MD | Paula García-González, BSc |  |  |
|  | Óscar Prada-Delgado, MD |  |  |  |
|  | Gonzalo Barge-Caballero, MD |  |  |  |
|  | Jose Ramon Gonzalez Juanatey, MD, PhD | Jose Seijas Amigo, Pharm | Santiago de Compostela | Hospital Clinico Universitario de Santiago (17) |
|  | Miguel Souto Bayarri, MD, PhD |  |  |  |
|  | Virginia Pubull Nuñez, MD |  |  |  |
|  | Raymundo Ocaranza Sanchez, MD, PhD |  |  |  |
|  | Belen Cid Alvarez, MD |  |  |  |
|  | Carlos Peña Gil, MD, PhD |  |  |  |
|  | Amparo Martinez Monzonis, MD |  |  |  |
|  | Alessandro Sionis, MD | Ana Fernández Martínez, RN | Barcelona | Hospital de la Santa Creu i Sant Pau (11) |
|  | Montserrat Vila Perales, MD |  |  |  |
|  | Josep Maria Padró, MD |  |  |  |
|  | Antonio Serra Peñaranda, MD |  |  |  |
|  | Joan García Picart, MD |  |  |  |
|  | Antonino Ginel Iglesias, MD |  |  |  |
|  | Xavier Garcia-Moll Marimon, MD |  |  |  |
|  | Guillem Pons Lladó, MD |  |  |  |
|  | Francesc Carreras Costa, MD |  |  |  |
|  | Vicente Miro, MD | Begoña Igual, MD | Valencia | Hospital Universitario y Politecnico La Fe (10) |
|  | Jose L Diez, MD |  |  |  |
|  | Pilar Calvillo, MD |  |  |  |
|  | F. Marin Ortuño, MD, PhD |  | Murcia | HUVA, Hospital Clínico Universitario Virgen De La Arrixaca (8) |
|  | M. Valdés Chávarri, MD, PhD | M. Quintana Giner, MD |  |  |
|  | A. Tello Montolliu, MD, PhD | A.I. Romero Aniorte, MD |  |  |
|  | E. Pinar Bermudez, MD, PhD | JM. Rivera Caravaca, MD |  |  |
|  | G. De La Morena, MD, PhD |  |  |  |
|  | Montserrat Gracida Blancas, MD | Olga Cañavate | Barcelona | Hospital De Bellvitge (4) |
|  |  | Sonia Guerrero |  |  |
|  |  | Silvia Riera |  |  |
|  | Jose Enrique Castillo Luena, MD | Jose Enrique Castillo Luena | Zaragoza | Hospital Universitario Miguel Servet (4) |
|  |  | Maria Lasala |  |  |
|  | Francisco Fernandez-Aviles, MD | Maria Lorenzo | Madrid | Hospital General Universitario Gregorio Maranon (2) |
|  |  | Olga Sobrino |  |  |
|  |  | Alexandra Vazquez |  |  |
| China (246) |  |  |  |  |
| Country Leader |  |  |  |  |
| Lixin Jiang, MD, PhD |  |  |  |  |
|  | Jiyan Chen, MD | Haojian Dong | Guangzhou | Guangdong General Hospital (102) |
|  |  | Peiyu He |  |  |
|  |  | Chunli Xia |  |  |
|  |  | Junqing Yang |  |  |
|  |  | Qi Zhong |  |  |
|  | Yongjian Wu, MD, PhD | Yanmeng Tian, MD | Beijing | Chinese Academy of Medical Sciences, Fuwai Hospital (17) |
|  |  | Dongze Li | Urumqi | First Affiliated Hospital of Xinjiang Medical University (15) |
|  | Yitong Ma, MD | Xiaomei Li |  |  |
|  | Yining Yang, MD | Xiang Ma |  |  |
|  |  | Zixiang Yu |  |  |
|  |  | Qian Zhao |  |  |
|  | Zheng Ji, MD | Chunguang Li | Tangshan | Tangshan Gongren Hospital (15) |
|  |  | Lei Zhang |  |  |
|  |  | Yu Zhao |  |  |
|  |  | Bolin Zhu |  |  |
|  | Xinchun Yang, MD | Mulei Chen | Beijing | Beijing Chao-yang Hospital, Capital Medical University (12) |
|  |  | Hongjie Chi |  |  |
|  |  | Yang Wang |  |  |
|  |  | Jing Zhang |  |  |
|  | Wenhua Lin, MD | Rui Jing | Tianjing | TEDA International Cardiovascular Hospital (12) |
|  |  | Jingjing Liu |  |  |
|  | Hesong Zeng, MD | Qiang Zhou, MD | Wuhan | Tongji Medical College (11) |
|  |  | Chang Xu, MD |  |  |
|  |  | Zhuxi Li, MD |  |  |
|  |  | Junhua Li, MD |  |  |
|  |  | Luyang Xiong, MD |  |  |
|  | Xin Fu, MD | Dan Gao | Zhengzhou | The First Affiliated Hospital of Zhengzhou University (11) |
|  |  | Dengke Jiang |  |  |
|  |  | Ran Leng |  |  |
|  |  | Xutong Wang |  |  |
|  |  | Qianqian Yuan |  |  |
|  |  | Lili Zhang |  |  |
|  | Bin Yang, MD | Ziliang Bai | Taiyuan | Shanxi Cardiovascular Hospital (10) |
|  |  | Jianhua Li |  |  |
|  |  | Jie Qi |  |  |
|  |  | Fei Wang |  |  |
|  |  | Haitao Wang |  |  |
|  |  | Bin Yang |  |  |
|  |  | Zhou Yue |  |  |
|  |  | Zhulin Zhang |  |  |
|  | Songtao Wang, MD | Yumei Dong | Qingdao | Qingdao Fuwai Hospital (8) |
|  |  | Jiajia Mao |  |  |
|  |  | Bin Zhang |  |  |
|  | Gong Cheng, MD | Xiuhong Li | Xian | Shanxi Provincial People’s Hospital (6) |
|  |  | Xiaowei Yao |  |  |
|  |  | Nier Zhong |  |  |
|  |  | Ning Zhou |  |  |
|  | Yulan Zhao, MD | Yaping Huang, MS | Zhengzhou | The Second Affiliated Hospital of Zhengzhou University (6) |
|  |  | Panpan Zhou, MS |  |  |
|  | Xuehua Fang, MD | Wei Su | Beijing | Liangxiang Hospital, Beijing Fangshan District (6) |
|  | Qiutang Zeng, MD | Yu Kunwu | Wuhan | Wuhan Union Hospital, Tongji Medical College, Huazhong Science and Tech University (3) |
|  |  | Yudong Peng |  |  |
|  |  | Xin Su |  |  |
|  | Xi Su, MD | Chen Wang | Wuhan | Wuhan Asia Heart Hospital (3) |
|  |  | Yunhai Zhao |  |  |
|  | Qingxian Li, MD | Yaming Geng | Jining | Affiliated Hospital of Jining Medical University (3) |
|  |  | Yanfu Wang |  |  |
|  | Shao-ping Nie, MD, PhD | Jing-yao Fan, MD | Beijing | Beijing Anzhen Hospital (2) |
|  |  | Si-ting Feng, MD,PhD |  |  |
|  |  | Xiao Wang, MD,PhD |  |  |
|  |  | Yan Yan, MD,PhD |  |  |
|  |  | Hui-min Zhang, MD,PhD |  |  |
|  | Qin Yu, MD | Lingping Chi | Dalian | Affiliated Zhongshan Hospital of Dalian University (2) |
|  |  | Fang Liu |  |  |
|  | Jian'an Wang, MD | Han Chen | Hangzhou | The Second Affiliated Hospital Zhejiang University School of Medicine (1) |
|  |  | Jun Jiang |  |  |
|  |  | Huajun Li |  |  |
|  |  | Jian'an Wang |  |  |
|  |  | Yechen Han, MM | Beijing | Peking Union Medical College Hospital (1) |
|  |  | Lihong Xu, RN |  |  |
|  | Shuyang Zhang, MD, PhD | Zhenyu Liu |  |  |
|  | Zhenyu Liu, MD | Gang Chen |  |  |
|  |  | Rongrong Hu |  |  |
| *Italy (139) |  |  |  |  |
| Country Leader |  |  |  |  |
| Aldo P. Maggioni, MD |  |  |  |  |
|  |  |  |  |  |
|  | Gian Piero Perna, MD | Francesca Pietrucci, PhD | Ancona | Cardiology and CCU - Ospedali Riuniti Ancona (54) |
|  | Marco Marini, MD |  |  |  |
|  | Gabriele Gabrielli, MD |  |  |  |
|  | Stefano Provasoli, MD | Anna Di Donato | Varese | Ospedale di Circolo e Fondazione Macchi (23) |
|  | Edoardo Verna, MD |  |  |  |
|  | Lorenzo Monti, MD |  | Rozzano | Humanitas Research Hospital, Rozzano (MI) (17) |
|  | Barbara Nardi, MD |  |  |  |
|  | Antonio Di Chiara, MD | Francesca Pezzetta, MD | Tolmezzo | Azienda Servizi Sanitaria n.3 Alto Friuli-Collinare-Medio Friuli (9) |
|  | Andrea Mortara, MD | Valentina Casali, MD | Monza | Policlinico di Monza, Monza MB (8) |
|  | Marcello Galvani, MD | Chiara Attanasio | Forli | Ospedale “G.B. Morgagni – L. Pierantoni” Forli (AUSL della Romagna) (8) |
|  | Filippo Ottani, MD |  |  |  |
|  | Marco Sicuro, MD | Gianpiero Leone, MD | Aosta | Ospedale Regionale Umberto Parini (5) |
|  |  | Francesco Pisano, MD |  |  |
|  |  | Cristina Bare, BSc |  |  |
|  | Paolo Calabro, MD | Fabio Fimiani | Napoli | AORN Dei Colli "V. Monaldi" UOC Cardiologia Università della Campania "L.Vanvitelli" (4) |
|  | Tiziana Formisano, MD |  |  |  |
|  | Giuseppe Tarantini, MD | Alberto Barioli, MD | Padua | University of Padua- Cardiology Clinic (3) |
|  | Umberto Cucchini, MD | Federica Ramani |  |  |
|  | Anto Luigi Andres, MD |  |  |  |
|  | Emanuela Racca, MD | Fabrizio Rolfo, MD | Cuneo | Azienda Ospedaliera S. Croce e Carle (3) |
|  |  | Cecilia Goletto |  |  |
|  | Carlo Briguori, MD | Francesca De Micco | Naples | Clinica Mediterranea (2) |
|  | Roberto Amati, MD | Stefano Di Marco, MD | Pescia | UO Cardiologia Ospedale SS Cosma e Damiano (2) |
|  | William Vergoni, MD | Martina Tricoli |  |  |
|  | Aldo Russo, MD | Massimo Villella, MD | San Giovanni Rotondo | IRCCS "Casa Sollievo della Sofferenza" (1) |
|  | Raffaele Fanelli, MD |  |  |  |
| *Singapore (61) |  |  |  |  |
| Country Leader |  |  |  |  |
| Harvey Douglas White, MD |  |  |  |  |
| Country Coordinator |  |  |  |  |
| Caroline Alsweiler |  |  |  |  |
|  | Kian-Keong Poh, MD |  | Singapore | National University Heart Center Singapore (33) |
|  | Ping Chai, MD |  |  |  |
|  | Titus Lau, MD |  |  |  |
|  | Joshua P. Loh, MD |  |  |  |
|  | Edgar L. Tay, MD |  |  |  |
|  | Kristine Teoh, MD | Sik-Yin V Tan, BSc |  |  |
|  | Lynette L. Teo, MD | Winnie C Sia, BSc |  |  |
|  | Ching-Ching Ong, MD | Audrey W Leong, BSc |  |  |
|  | Raymond C. Wong, MD |  |  |  |
|  | Poay-Huan Loh, MD |  |  |  |
|  | Theodoros Kofidis, MD |  |  |  |
|  | Wan Xian Chan, MD |  |  |  |
|  | Koo Hui Chan, MD |  |  |  |
|  | David Foo, MBBS | Li Hai Yan, RN | Singapore | Tan Tock Seng Hospital (22) |
|  | Jason Loh Kwok Kong, MD |  |  |  |
|  | Ching Min Er, MD |  |  |  |
|  | Fahim Haider Jafary, MD |  |  |  |
|  | Terrance Chua, MD | Nasrul Ismail | Singapore | National Heart Centre Singapore (6) |
|  |  | Min Tun Kyaw |  |  |
|  |  | Deborah Yip |  |  |
| Germany (54) |  |  |  |  |
| Country Leader |  |  |  |  |
| Rolf Doerr, MD |  |  |  |  |
|  | Rolf Doerr, MD |  | Dresden | Praxisklinik Herz und Gefaesse (29) |
|  | Juergen Stumpf, MD | Dorit Grahl |  |  |
|  | Klaus Matschke, MD, PhD | Franziska Guenther |  |  |
|  | Gregor Simonis, MD, PhD | Kerstin Bonin |  |  |
|  | Clemens T. Kadalie, MD |  |  |  |
|  | Udo Sechtem, MD | Ina Wenzelburger | Stuttgart | Robert-Bosch-Krankenhaus (22) |
|  | Peter Ong, MD | Susanne Gruensfelder, RN |  |  |
|  | P. Christian Schulze, MD, PhD |  | Jena | University Hospital Jena (2) |
|  | Bjoern Goebel, MD |  |  |  |
|  | Karsten Lenk, MD |  |  |  |
|  | Georg Nickenig, MD | Jan-Malte Sinning, MD | Bonn | Universitatsklinikum Bonn (1) |
|  |  | Marcel Weber, MD |  |  |
|  |  | Nikos Werner, MD |  |  |
| Austria (50) |  |  |  |  |
| Country Leaders |  |  |  |  |
| Irene Marthe Lang, MD |  |  |  |  |
| Kurt Huber, MD |  |  |  |  |
|  | Herwig Schuchlenz, MD | Gudrun Steinmaurer | Graz | LKH Graz West Austria (35) |
|  | Stefan Weikl, MD |  |  |  |
|  | Irene Marthe Lang, MD | Max-Paul Winter, MD | Vienna | Medical University of Vienna, Department of Cardiology (8) |
|  |  | Tijana, Andric, MD | Vienna | Wilhelminen Hospital Vienna (7) |
|  | Kurt Huber, MD | Maximilian, Tscharre, MD |  |  |
|  | Gabriele, Jakl-Kotauschek, MD | Claudia, Wegmayr, MSc |  |  |
|  |  | Bernhard, Jäger, MD |  |  |
|  |  | Florian, Egger, MD |  |  |
| Hungary (49) |  |  |  |  |
| Country Leader |  |  |  |  |
| Matyas Keltai, MD, PhD, DSc |  |  |  |  |
|  | Andras Vertes, MD | Judit Sebo, MD | Budapest | Eszszk- Szent Istvan Hospital (20) |
|  |  | Zoltan Davidovits, MD |  |  |
|  |  | Laszlone Matics |  |  |
|  | Albert Varga, MD, PhD | Gergely Ágoston, MD | Szeged | University of Szeged (12) |
|  | Geza Fontos, MD | Gabor Dekany, MD | Budapest | George Gottsegen National Institute of Cardiology (9) |
|  | Bela Merkely, MD, PhD, DSc | Andrea Bartykowszki, MD | Budapest | Heart and Vascular Center, Semmelweis University (8) |
|  |  | Pal Maurovich-Horvat, MD, PhD, MPH | |  |
|  | Gabor Kerecsen, MD | Agnes Jakal | Budapest | Military Hospital, Budapest (1) |
| Serbia (47) |  |  |  |  |
|  | Sasa Hinic, MD, BSc | Jelena Djokic, MD | Belgrade | University Hospital Center Bezanijska Kosa (13) |
|  | Marija Zdravkovic, MD, PhD |  |  |  |
|  | Vladan Mudrenovic, MD |  |  |  |
|  | Bogdan Crnokrak, MD |  |  |  |
|  | Branko D. Beleslin, MD, PhD |  | Belgrade | Faculty of Medicine, University of Belgrade; Cardiology Clinic, Clinical Center of Serbia (10) |
|  | Nikola N. Boskovic, MD | Ana D. Djordjevic-Dikic, MD, PhD |  |  |
|  | Marija T. Petrovic, MD | Vojislav L. Giga, MD, PhD |  |  |
|  | Milan R. Dobric, MD | Jelena J. Stepanovic, MD, PhD |  |  |
|  | Zeljko Z. Markovic, MD, PhD |  |  |  |
|  | Ana S. Mladenovic, MD, PhD |  |  |  |
|  | Nada Cemerlic-Adjic, MD | Lazar Velicki, MD | Sremska Kamenica | Institute of Cardiovascular Diseases Vojvodina, Sremska Kamenica, Serbia and Faculty of Medicine, University of Novi Sad (9) |
|  |  | Ljiljana Pupic |  |  |
|  | Goran Davidović, MD, PhD | Stefan M. Simović, MD | Kragujevac | Clinical Center Kragujevac (7) |
|  | Rada Vučić, MD |  |  |  |
|  | Milica Nikola Dekleva, MD PhD | Miroslav Stevo Martinovic, MD | Belgrade | University Clinical Hospital Zvezdara (6) |
|  |  | Gordana Stevanovic |  |  |
|  | Goran Stankovic, MD | Milan Dobric | Belgrade | Clinical Center of Serbia (1) |
|  | Svetlana Apostolovic, MD | Sonja Salinger Martinovic | Nis | Clinic for Cardiovascular Diseases, Clinical Center Nis (1) |
|  |  | Dragana Stanojevic |  |  |
| Mexico (46) |  |  |  |  |
|  | Jorge Escobedo, MD | Ramon de Jesús-Pérez, RN | Benito Juarez | Instituto Mexicano del Seguro Social (35) |
|  | Rubén Baleón-Espinosa, MD |  |  |  |
|  | Arturo S Campos-Santaolalla, MD |  |  |  |
|  | Elihú Durán-Cortés, MD |  |  |  |
|  | José M Flores-Palacios, MD |  |  |  |
|  | Andrés García-Rincón, MD |  |  |  |
|  | Moisés Jiménez-Santos, MD |  |  |  |
|  | Joaquín V Peñafiel, MD |  |  |  |
|  | José A Ortega-Ramírez, MD |  |  |  |
|  | Aquiles Valdespino-Estrada, MD |  |  |  |
|  | Erick Alexánderson Rosas, MD | María Fernanda Canales Brassetti, MD | Mexico City | Instituto Nacional de Cardiología "Ignacio Chávez" (11) |
|  |  | Diego Adrián Vences Anaya, MD |  |  |
|  |  | María Pérez García |  |  |
|  |  | Isabel Estela Carvajal Juarez , MD |  |  |
|  |  | Magdalena Madero Rovalo, MC |  |  |
|  |  | Erick Donato Morales Rodríguez, MD | |  |
| Australia (45) |  |  |  |  |
| Country Leaders |  |  |  |  |
| Joseph B. Selvanayagam, MBBS (Hons), DPhil | |  |  |  |
| Jamie Rankin, MBBS *(past)* |  |  |  |  |
| Country Coordinator |  |  |  |  |
| Deirdre Murphy |  |  |  |  |
|  | Joseph B. Selvanayagam, MBBS (Hons), DPhil | Sau Lee, PhD | Adelaide | Flinders Medical Centre (30) |
|  | Majo X. Joseph, MBBS | Prince Thomas, RN |  |  |
|  | Suku T. Thambar, MBBS | Melissa D Chaplin, RN | New Lambton Heights | John Hunter Hospital (8) |
|  |  | Stephanie C Boer, B Biotechnology (Honours) | |  |
|  | John F. Beltrame, MD | Jeanette K. Stansborough, RN | Woodville South | The Queen Elizabeth Hospital (5) |
|  |  | Marilyn Black, RN |  |  |
|  | Graham S. Hillis, PhD | Michelle M. Bonner, B. Nursing | Perth | Royal Perth Hospital (2) |
|  |  | Kim F. Ireland, RN |  |  |
|  |  | Clare Venn-Edmonds, RN |  |  |
| France (42) |  |  |  |  |
| Country Leader |  |  |  |  |
| Philippe-Gabriel Steg, MD |  |  |  |  |
| Country Coordinators |  |  |  |  |
| Helene Abergel |  |  |  |  |
| Jean-Michel Juliard |  |  |  |  |
|  |  | Corine Thobois, RN | Chartres | C.H. Louis Pasteur (21) |
|  | Christophe Thuaire, MD | Emilie Tachot, RN |  |  |
|  | Téodora Dutoiu, MD | Christophe Laure, RN |  |  |
|  |  | Christel Vassaliere, RN |  |  |
|  | Philippe Gabriel Steg, MD | Helene Abergel, MSc | Paris | Bichat Hospital (9) |
|  | Jean-Michel Juliard, MD | Axelle Fuentes, MSc |  |  |
|  | Michel S. Slama, MD | Ludivine Eliahou, MD | Clamart Cedex | Antoine-Beclere Hospital (5) |
|  | Rami El Mahmoud, MD | Olivier Dubourg, MD | Boulogne | Ambroise Pare Hospital (2) |
|  |  | Pierre Michaud, MD |  |  |
|  | Eric Nicollet, MD | Sarah Hadjih | Corbeil-Essonnes Cedex | Centre Hospitalier Sud Francilien (2) |
|  | Pascal Goube, MD | Patricia Brito |  |  |
|  | Gilles Barone-Rochette, MD | Gilles Barone-Rochette | Grenoble | Grenoble University Hospital (2) |
|  | Alain Furber, MD | Charles Cornet, MD, PhD | Angers Cedex 9 | Centre Hospitalier Universitaire d'Angers (1) |
|  | Loïc Bière, MD | Jeremy Rautureau, MD, PhD |  |  |
| Lithuania (39) |  |  |  |  |
|  |  | Agne Juceviciene, MD | Vilnius | Vilnius University Hospital Santariskes Clinic (39) |
|  |  | Irma Kalibataite-Rutkauskiene, MD |  |  |
|  |  | Laura Keinaite |  |  |
|  | Aleksandras Laucevicius, MD | Monika Laukyte |  |  |
|  | Jelena Celutkiene, MD | Gelmina Mikolaitiene |  |  |
|  |  | Akvile Smigelskaite, MD |  |  |
|  |  | Ilona Tamasauskiene, MD |  |  |
|  |  | Agne Urboniene, MD |  |  |
| *Netherlands (37) |  |  |  |  |
|  | Elvin Kedhi MD, PhD |  | Zwolle | Isala Klinieken (25) |
|  | Jorik Timmer, MD | Ilse Bouwhuis |  |  |
|  | Rik Hermanides, MD | Lia Nijmeijer |  |  |
|  | Eliza Kaplan, MD |  |  |  |
|  | Robert K. Riezebos, MD, PhD |  | Amsterdam | Cardio Research Hartcentrum OLVG (11) |
|  | Pouneh Samadi, MD | Jeannette, J. M. Schoep, RN |  |  |
|  | Elise van Dongen, MD | Elisabeth, M. Janzen, RN |  |  |
|  | Sander R. Niehe, MD |  |  |  |
|  | Harry Suryapranata, MD | Sandra Ahoud | Nijmegen | Radboudumc (1) |
|  | Stijn van Vugt, MD, PhD |  |  |  |
| Portugal (33) |  |  |  |  |
|  | Ruben Ramos, MD |  | Lisbon | Hospital de Santa Marta (25) |
|  | Duarte Cacela, MD |  |  |  |
|  | Ana Santana, MD |  |  |  |
|  | Antonio Fiarresga, MD |  |  |  |
|  | Lidia Sousa, MD |  |  |  |
|  | Hugo Marques, MD |  |  |  |
|  | Lino Patricio, MD | Mafalda Selas |  |  |
|  | Luis Bernanrdes, MD | Filipa Silva |  |  |
|  | Pedro Rio, MD | Cláudia Freixo |  |  |
|  | Ramiro Carvalho, MD |  |  |  |
|  | Rui Ferreira, MD |  |  |  |
|  | Tiago Silva, MD |  |  |  |
|  | Ines Rodrigues, MD |  |  |  |
|  | Pedro Modas, MD |  |  |  |
|  | Guilherme Portugal, MD |  |  |  |
|  | Jose Fragata, MD |  |  |  |
|  | Fausto J. Pinto, PhD | Inês Zimbarra Cabrita, PhD | Lisbon | Santa Maria University Hospital, Cardiology Department, CHLN (6) |
|  | Miguel Nobre Menezes, MD | Andreia Rocha, MSc |  |  |
|  | Guilhermina Cantinho Lopes, MD | Francisca Patuleia Figueiras, PhD |  |  |
|  | Ana Gomes Almeida, PhD | Andreia Coelho, BSc |  |  |
|  | Pedro Canas Silva, MD | Marta Capinha |  |  |
|  | Angelo Nobre, MD | Maria Inês Caetano |  |  |
|  | Ana Rita Francisco, MD | Susana Silva |  |  |
|  | Nuno Ferreira, MD |  | Vila Nova de Gaia | Centro Hospitalar de Vila Nova de Gaia/Espinho, EPE (2) |
|  | Ricardo L. Lopes, MD |  |  |  |
| Argentina (29) |  |  |  |  |
| Country Leader |  |  |  |  |
| Rafael Diaz, MD *(past)* |  |  |  |  |
|  | Luis Guzman, MD | Veronica Tinnirello | Cordoba | Instituto Medico DAMIC (11) |
|  | Julio César Figal, MD | Matías Nicolás Mungo | Ciudad Autonoma de Buenos Aires | Fundación Favaloro (10) |
|  | Oscar Méndiz, MD |  |  |  |
|  | Claudia Cortés, MD |  |  |  |
|  | Roberto René Favaloro, MD |  |  |  |
|  | Carlos Alvarez, MD | Marina Garcia | Bahia Blanca | Hospital Italiano Regional del Sur Bahia Blanca (3) |
|  | Javier Courtis, MD | Valeria Godoy | Cordoba | Clinica Romagosa and Clinica De La Familia (2) |
|  | Gabriela Zeballos, MD |  |  |  |
|  | Lilia Schiavi, MD | Maria Victoria Actis | Cordoba | Clinica Del Prado (2) |
|  | Mariano Rubio, MD | Graciela Scaro, MD | Cordoba | Clínica Privada Vélez Sarsfield (1) |
| *New Zealand (28) |  |  |  |  |
| Country Leader |  |  |  |  |
| Harvey Douglas White, MD |  |  |  |  |
| Country Coordinator |  |  |  |  |
| Caroline Alsweiler |  |  |  |  |
|  | Gerard Patrick Devlin, MD | Liz Low, RN | Hamilton | Waikato Hospital (22) |
|  | Raewyn Fisher, MD | Jayne Scales, RN |  |  |
|  |  | Kirsty Abercrombie, RN |  |  |
|  | Ralph Alan Huston Stewart, MCChB, MD | Leah Howell , RN | Auckland | Auckland City Hospital (6) |
|  | Harvey Douglas White, MD | Cathrine Patten, RN |  |  |
|  | Jocelyne Benatar, MD |  |  |  |
| *Macedonia (28) |  |  |  |  |
|  | Sasko Kedev, MD, PhD |  | Skopje | University Clinic of Cardiology (28) |
|  | Irena Peovska Mitevska, MD, PhD |  |  |  |
|  | Elizabeta Srbinovska Kostovska, MD, PhD |  |  |  |
|  | Hristo Pejkov, MD, PhD |  |  |  |
| *Sweden (23) |  |  |  |  |
| Country Leader |  |  |  |  |
| Claes Held, MD, PhD |  |  |  |  |
|  | Claes Held, MD, PhD |  | Uppsala | Uppsala University (18) |
|  | Kai Eggers, MD, PHhD |  |  |  |
|  | Gunnar Frostfelt, MD, PhD | Christina Björklund, RN |  |  |
|  | Nina Johnston, MD, PhD | Maria Andreasson, RN |  |  |
|  | Maciej Olsowka, MD | Marie Essermark, RN |  |  |
|  | Axel Åkerblom, MD, PhD |  |  |  |
|  | Inga Soveri, MD, PhD |  |  |  |
|  | Johannes Aspberg, MD | Liselotte Persson | Stockholm | Karolinska Institutet at Danderyd Hospital (5) |
| Israel (15) |  |  |  |  |
| Country Leaders |  |  |  |  |
| Rafael Beyar, MD, MD, DSc, MPH | |  |  |  |
| Tali Sharir, MD |  |  |  |  |
| Country Coordinator |  |  |  |  |
| Eugenia Nikolsky, MD |  |  |  |  |
|  | Tali Sharir, MD | Or Harel, MA | Tel-Aviv | Assuta Medical Centers (9) |
|  | Dan Elian, MD |  |  |  |
|  | Arthur Kerner, MD | Margalit Bentzvi | Haifa | Rambam Medical Center (6) |
|  | Samia Massalha, MD | Ludmila Helmer |  |  |
| Japan (14) |  |  |  |  |
| Country Leader |  |  |  |  |
| Shun Kohsaka, MD |  |  |  |  |
|  | Keiichi Fukuda, MD, PhD | Ikuko Ueda, PhD | Shinjuku-ku | Keio University (7) |
|  | Shun Kohsaka, MD | Jun Fujita, MD |  |  |
|  | Satoshi Yasuda, MD, PhD | Akemi Furukawa, RN | Suita-shi | National Cerebral and Cardiovascular Center |
|  |  | Kanae Hirase, RN |  | -4 |
|  |  | Toshiyuki Nagai, MD, PhD |  |  |
|  |  | Fumiyuki Otsuka, MD, PhD |  |  |
|  | Shigeyuki Nishimura, MD | Shintaro Nakano | Hidaka | Saitama Medical University (3) |
| *Belgium (7) |  |  |  |  |
| Country Leader |  |  |  |  |
| Frans Van de Werf, MD, PhD |  |  |  |  |
| Country Coordinator |  |  |  |  |
| Kaatje Goetschalckx, MD |  |  |  |  |
|  | Kaatje Goetschalckx, MD | Valerie Robesyn | Leuven | University Hospital Leuven (7) |
|  | Frans Van de Werf, PhD, MD |  |  |  |
|  | Kathleen Claes, PhD, MD |  |  |  |
| *Taiwan (7) |  |  |  |  |
| Country Leader |  |  |  |  |
| Harvey Douglas White, MD |  |  |  |  |
| Country Coordinator |  |  |  |  |
| Caroline Alsweiler |  |  |  |  |
|  | Chung-Lieh Hung, MD | Yi-Hsuan Yang | Taipei City | Mackay Memorial Hospital (7) |
|  | Chun-Ho Yun, MD |  |  |  |
|  | Charles Jia-Yin Hou, MD |  |  |  |
|  | Jen-Yuan Kuo, MD |  |  |  |
|  | Hung-I Yeh, MD, PhD |  |  |  |
|  | Ta-Chuan Hung, MD |  |  |  |
|  | Jiun-Yi Li , MD, PhD |  |  |  |
|  | Chen-Yen Chien, MD, PhD |  |  |  |
|  | Cheng-Ting Tsai, MD |  |  |  |
|  | Chun-Chieh Liu, MD |  |  |  |
|  | Fa-Chang Yu, MD |  |  |  |
|  | Yueh-Hung Lin, MD |  |  |  |
|  | Wei-Ren Lan, MD |  |  |  |
|  | Chih-Hsuan Yen, MD |  |  |  |
|  | Jui-Peng Tsai, MD |  |  |  |
|  | Kuo-Tzu Sung, MD |  |  |  |
| *South Africa (7) |  |  |  |  |
|  | Mpiko Ntsekhe, MD |  | Cape Town | Groote Schuur Hospital / University of Cape Town (7) |
|  | Shaheen Pandie, MD | Constance Philander (Nee Talliard), ND | |  |
|  | Charle A Viljoen, MD | Noloyiso Mtana, RN |  |  |
|  | Marianne De Andrade, MD |  |  |  |
| *Switzerland (7) |  |  |  |  |
| Country Leader |  |  |  |  |
| Aldo P. Maggioni, MD |  |  |  |  |
|  | Tiziano Moccetti, MD | Adriana Anesini, RN | Lugano | Cardiocentro (7) |
|  | M.Grazia Rossi, MD | Simona Maspoli, RN |  |  |
|  |  | Manuela Mombelli, RN |  |  |
| Egypt (6) |  |  |  |  |
|  | Magdy Abdelhamid, MD | Ahmed Talaat, MD | Cairo | Cairo University (6) |
|  | Ahmed Adel, MD |  |  |  |
|  | Ahmed Kamal, MsC |  |  |  |
|  | Hossam Mahrous, MD |  |  |  |
|  | Sameh El Kaffas, MD |  |  |  |
|  | Hussien El Fishawy, MD |  |  |  |
| Romania (5) |  |  |  |  |
|  | Calin Pop, MD, PhD |  | Bucharest | Emergency County Hospital Baia Mare (4) |
|  | Matei Claudia, MD, PhD |  |  |  |
|  | Bogdan A. Popescu, MD, PhD |  | Bucharest | Emergency Institute of Cardiovascular Diseases ''Prof. Dr. C. C. Iliescu'' (1) |
|  | Carmen Ginghina, MD, PhD | Monica Rosca, MD, PhD |  |  |
|  | Dan Deleanu, MD, PhD | Carmen C. Beladan, MD, PhD |  |  |
|  | Vlad A. Iliescu, MD, PhD |  |  |  |
| *Saudi Arabia (5) |  |  |  |  |
|  | Mouaz H. Al-Mallah, MD MSc | Sarah Zahrani, RN | Central Province | King AbdulAziz Cardiac Center (5) |
|  | Ahmed Aljzeeri, MD |  |  |  |
|  | Hani Najm, MD |  |  |  |
|  | Ali Alghamdi, MD |  |  |  |
| *Peru (4) |  |  |  |  |
|  | Walter Enrique Mogrovejo Ramos, MD | Marco Antonio Monsalve Davila, RN | Mirafloes | Instituto Neuro Cardiovascular De Las Americas (4) |
| Thailand (3) |  |  |  |  |
| Country Leader |  |  |  |  |
| Harvey Douglas White, MD |  |  |  |  |
| Country Coordinator |  |  |  |  |
| Caroline Alsweiler |  |  |  |  |
|  | Srun Kuanprasert, MD |  | Chiang Mai | Maharaj Nakorn Chiang Mai Hospital (2) |
|  | Arintaya Prommintikul, MD |  |  |  |
|  | Weerachai Nawarawong, MD | Supatchara Khwakhong, RN |  |  |
|  | Surin Woragidpoonpol, MD | Anong Chaiyasri, RN |  |  |
|  | Thitipong Tepsuwan, MD | Warangkana Mekara, RN |  |  |
|  | Noppon Taksaudom, MD | Supap Kulthawong, RN |  |  |
|  | Chataroon Rimsukcharoenchai, MD | Anong Amaritakomol, RN |  |  |
|  | Juntima Euathrongchit, MD |  |  |  |
|  | Yutthaphan Wannasopha, MD |  |  |  |
|  | Sukit Yamwong, MD | Pachara Panpunuan, RN | Bangkok | Ramathibodi Hospital (1) |
|  | Piyamitr Sritara, MD |  |  |  |
|  | Suthara Aramcharoen, MD |  |  |  |
|  | Krissada Meemuk, MD |  |  |  |
| *Malaysia (2) |  |  |  |  |
| Country Leader |  |  |  |  |
| Harvey Douglas White, MD |  |  |  |  |
| Country Coordinator |  |  |  |  |
| Caroline Alsweiler |  |  |  |  |
|  | Ahmad Khairuddin, MD | Noor Syamira Mokhtar, RN | Kuala Lumpur | Institut Jantung Negara (2) |
|  | Hafidz Abd Hadi, MD | Nor Asiah Basri, RN |  |  |
|  | Shaiful Azmi Yahaya, MD | Irni Yusnida, RN |  |  |
|  |  | Humayrah Hashim |  |  |
|  |  |  |  |  |
| ** Countries participated in Economics Quality of Life (EQoL) Questionnaires* | | |  |  |
| ***This site received one participant in transfer that was randomized at another site* | | |  |  |
